# Investigating fungal diversity through metabarcoding for environmental samples: assessment of ITS1 and ITS2 Illumina sequencing using multiple defined mock communities with different classification methods and reference databases

**DOI:** 10.1186/s12864-025-11917-y

**Published:** 2025-08-06

**Authors:** Raf Winand, Elizabet D’hooge, Alexander Van Uffelen, Bert Bogaerts, Julien Van Braekel, Stefan Hoffman, Nancy H. C. J. Roosens, Pierre Becker, Sigrid C. J. De Keersmaecker, Kevin Vanneste

**Affiliations:** 1 Transversal activities in Applied Genomics, Sciensano, 1050 Brussels, Belgium; 2 BCCM/IHEM collection, Mycology and Aerobiology, Sciensano, 1050 Brussels, Belgium; 3 Department of Information Technology, Internet Technology and Data Science Lab (IDLab), Interuniversity Microelectronics Centre (IMEC), Ghent University, 9052 Ghent, Belgium

**Keywords:** Fungal identification, ITS, Targeted metagenomics, Illumina, Metabarcoding, Orientation

## Abstract

An important challenge in taxonomic classification of environmental samples is capturing the real diversity by identifying all species present in a sample. Metabarcoding approaches are often employed to identify species in complex samples. The internal transcribed spacer (ITS) region is the official, widely adopted, barcode for identifying fungal species. Metabarcoding can be done in many different ways with multiple choices at different steps of the workflow. We present a comparative evaluation of the sequenced region (ITS1 and/or ITS2), two different reference databases (UNITE versus BCCM/IHEM), two different bioinformatics software packages (BLAST versus mothur), and the considered taxonomic level (species versus genus level), to accurately capture the diversity using 37 fungal defined mock communities (DMCs). The DMCs cover a broad range of fungal diversity, including 42 Ascomycota species (26 genera), 4 Basidiomycota species (4 genera), and 5 Mucoromycota species (5 genera), all commonly found in indoor environments in Western Europe. Classification performance was first evaluated using ITS1 and ITS2 sequences of all species in the DMCs, generated by Sanger sequencing, to evaluate the discriminatory power of ITS and set a baseline for subsequent comparison with Illumina sequencing. Classification performance was found to be variable depending on all considered variables (sequencing technology, taxonomic level, ITS region, software, database) with 56–100% of species correctly assigned. Sanger sequencing showed that neither ITS1 nor ITS2 resulted in optimal performance due to its low discriminatory power within certain genera. Compared to Sanger sequencing, Illumina sequencing generally resulted in lower precision but comparable recall. Classification performance was generally good at genus but not at species level, although intermediate taxonomic levels could present adequate alternatives. ITS2 typically resulted in slightly better precision and comparable recall compared to ITS1. The employed reference database had a marked effect, with BCCM/IHEM performing better than UNITE due to the difference in number of sequences in each database. BLAST resulted in better performance, but required expert curation, whereas mothur performed better when using an automated workflow. Estimating species abundances using Illumina sequencing read counts generally performed only poorly, although read abundance filtering could increase the precision of ITS1, but not ITS2. Each approach comes with its own advantages and inconveniences and should be carefully selected based on the objectives of the analysis. Our results highlight the power of metabarcoding using Illumina sequencing for investigating fungal diversity in complex samples and can guide scientists in selecting the most appropriate setup for their own purposes.

**Supplementary Information:**

The online version contains supplementary material available at 10.1186/s12864-025-11917-y.

## Background

Fungi are a group of very diverse and omnipresent organisms fulfilling an equally wide range of roles. For instance, they play an important environmental role by providing nutrients to other organisms and by cycling organic matter. They are used in pest control to defend against agricultural losses, and in the production of medicines and food. On the other hand, fungi also represent major contaminants of the human environment and of various products and matrices. They can cause allergic reactions and be pathogenic in humans, animals and plants [1, 2]. Understanding the composition of fungal communities across various ecosystems and samples is hence of paramount importance.

Metabarcoding, i.e., employing short genetic markers to identify species by means of high-throughput sequencing (HTS) technologies to simultaneously analyse multiple organisms in a single sample, is commonly used to characterize fungal diversity in complex environmental samples. However, accurately identifying the real diversity poses a substantial challenge as several factors need to be considered. One essential aspect is the exact marker region employed. In fungal metabarcoding, the internal transcribed spacer (ITS) region is the most commonly used DNA region because of several reasons: (i) it is present in the genome of every fungal taxon; (ii) it is a well-conserved region but still contains sufficient variety in many genera to discriminate between species; (iii) it can be easily amplified; (iv) ITS became the official fungal barcode in 2012, recognized by the Consortium for the Barcode of Life [3, 4], after which the amount of ITS sequences in public databases has substantially increased and currently covers the majority of known taxa of the fungal kingdom. The main drawback of ITS is that for some ubiquitous fungal genera (e.g., *Aspergillus*, *Penicillium*), ITS does not always provide enough discriminatory power for identification at the species level [4–6]. While other commonly used ribosomal markers (e.g. RNA polymerase II largest subunit (*RPB1*) and RNA polymerase II second largest subunit (*RPB2*)) may perform better for fungal species identification, they have a much lower amplification success rate compared to ITS due to primer failure [4]. Protein-coding gene regions such as calmodulin (*CaM*) and beta-tubulin (*benA*) could be better suited for identifying closely-related taxa at the species level, but they are generally only suitable within certain groups of fungi (e.g. *Penicillium*, *Aspergillus*, *Fusarium*) and hence don’t cover the entire diversity of the fungal kingdom as well as ITS [7]. The translation elongation factor 1-α *(TEF1α*) gene region constitutes the second official barcode for fungi but public databases contain considerably less *TEF1α* sequences than ITS sequences, rendering it less useful for metabarcoding [8].

The complete ITS region, typically 500–700 bp long, does however present a challenge for sequencing platforms generating short reads such as the widely used Illumina technology with read lengths of 150–300 bp. The ITS fragment length results in decreased sequencing efficiency, since longer fragments create larger clusters, reducing the maximum number of clusters that can be sequenced on the flow cell. Furthermore, the length of entire ITS fragments can be longer than the combined length of forward and reverse reads on short-read platforms, leading to a lack of sequence overlap for merging paired reads. Also, reads on short-read platforms may have higher error rates towards their ends, making it harder to combine the forward and reverse reads into a single ITS sequence. Thus, high sequencing accuracy and coverage across the entire length of the ITS region cannot be guaranteed. Although Sanger sequencing does allow sequencing the entire ITS region, it has as drawback that it does not allow direct sequencing of a mixed community, rendering it impractical when multiple species are present in a sample and/or when they are difficult to cultivate. To address the sequence length limitations of short-read platforms, specific subregions of ITS are targeted for sequencing, such as the ITS1 or ITS2 region. The ITS1 region is located between the 18S and the 5.8S rRNA genes and is typically 150–400 bp long, while the ITS2 region is located between the 5.8S and the 28S rRNA genes and shares a similar length range with ITS1. The scientific community lacks consensus on the optimal marker for fungal metabarcoding, with some studies advocating for ITS1 and others favouring ITS2. According to Mbareche *et al.* [9], more species could be recovered from ITS1 metabarcoding compared to using ITS2. However, some taxa may be underrepresented or absent in ITS1 sequence databases, and vice versa for ITS2. Conversely, other studies found ITS2 to be the preferred marker for exploring fungal diversity, as it recovers taxonomic profiles that more closely resemble those of the entire ITS region [10, 11]. Additionally, ITS1 exhibits more variability in terms of length, GC content, and polymorphisms, has fewer universal primer sites compared to ITS2, and might lead to an overestimation of the diversity and number of species detected in a sample [10, 12].

A database containing correctly annotated reference and representative sequences is also crucial for correct identification. An issue with public repositories is that many sequences are incorrectly assigned to certain species or that information is incomplete, resulting in incorrect or inaccurate classifications [13]. Additionally, fungi have other scientifically useful subgeneric levels at which they can be classified, namely the section, series and species complex, which are not always available in databases for all genera as they are sometimes simply not included and other times because some genera cannot be subdivided into these levels as their phylogeny has not been completely resolved yet [14, 15]. Intragenomic variation occurring due to differences in ITS copy numbers further confounds this issue, as in some cases it can even exceed interspecific variation [16]. Nilsson *et al.* found that up to 20% of sequences in public repositories can be incorrectly annotated [17]. To address this drawback, a number of publicly available and curated databases were developed to provide reliable resources for fungal classification based on ITS sequences. These include the UNITE ITS database [18, 19] and the NCBI Reference Sequence Targeted Loci (RTL) ITS database [20], but also more specialized databases for pathogenic fungi such as the International Society of Human and Animal Mycology (ISHAM) database [21] or the Q-Bank database [22]. The UNITE database is the most widely used reference database, and is based on a community effort, providing reference sequences that are clustered into ‘species hypotheses’, i.e., sequences that can be interpreted as species. Beside these public databases, research groups can also create their own custom databases that are specifically tailored to their needs. For instance, Sciensano (the Belgian institute for health) hosts the BCCM/IHEM (Belgian coordinated collections of microorganisms/ IHEM Fungi collection: human and animal health) culture collection of medical and veterinary fungi containing more than 17,000 strains and species [23].

Another consideration is the employed bioinformatics package(s) for analysing sequencing data. This software can range from a simple BLAST search against any reference database to more complex software suites such as mothur [24] and QIIME [25], which are well-known tools for analysing metagenomic samples that offer extensive functionality and implement different algorithms such a naive Bayesian classifiers. These tools are generally highly configurable, allowing tweaking settings for maximal performance, with the caveat that it is impossible to determine the performance on samples of unknown composition as there is no ground truth to compare to. To address this issue, defined mock communities (DMCs) are often utilized as they provide a ground truth for which the species composition is known [26, 27]. By comparing the performance of different software, settings and databases for the classification of DMCs, it becomes possible to evaluate their strengths and weaknesses in general or specific to the DMC composition.

The purpose of this study was to provide insight into the optimal setup for metabarcoding analysis using Illumina sequencing to investigate fungal diversity in complex environmental samples. We assessed the effectiveness of various factors, such as the impact of different sequenced regions (ITS1 versus ITS2), reference databases (UNITE versus BCCM/IHEM), bioinformatics software packages (BLAST versus mothur), and taxonomic levels (species versus genus). Moreover, for thorough evaluation and optimization, we created a curated set of 37 DMCs representing a wide variety of fungal species.

## Materials and methods

### Defined mock communities

For the Illumina sequencing, 37 DMCs were constructed in vitro (Supplementary Table 1) starting from individual species for which the ITS regions were previously sequenced using Sanger sequencing and for which DNA was available. Overall, the DMCs contained 51 different fungal species, belonging to 35 different genera. The species were obtained from the BCCM/IHEM culture collection of medical and veterinary fungi [23]. This collection contains more than 17,000 public strains, consisting of clinical isolates, allergy-inducing species, mycotoxin-producing strains and environmental isolates. The identification in the BCCM/IHEM collection is manually curated by mycologists down to the strain level, while for this study all analyses were limited to the species level. The selected species frequently occur in the indoor environment in Western Europe and represent a broad coverage of the fungal kingdom belonging to the Ascomycota, Basidiomycota and Mucoromycota.

These species were cultured on Sabouraud dextrose broth at room temperature and constant agitation, for 5 to 8 days. Genomic DNA of each species was extracted with the Plant Invisorb Spin Plant Mini Kit (Invitek, Berlin, Germany) or the ZR Fungal/Bacterial DNA kit (Zymo Research, Freiburg, Germany), with the following adaptations: (i) before lysis, the fungal material was frozen at -80 °C for at least 30 min and subsequently lyophilized overnight. This was followed by (ii) a bead beating step to disrupt the fungal cell wall and (iii) a longer lysis time of 2 to 2.5 h for the Invitek kit. The DNA concentration and quality were checked with the Nanodrop 2000 (Thermo Scientific, Wilmington, USA). Each DMC consisted of five different species of which genomic DNA was added in equal quantities in order to exclude differences in abundance as a confounding factor in the analysis. This was done by mixing equal concentrations and equal volumes (20 ng/µl per species, 5 µl) of five different species, for a total volume of 25 µl. All DMCs contained species belonging to five different genera except for DMCs 1, 18, 19, and 34, which contained four different genera. The last two DMCs were technical replicates with an identical composition (DMC 36 and DMC 37).

For the analysis of the Sanger sequences, the available sequences were mixed in silico to match the composition of the different DMCs.

### IHEM database– Sanger sequencing

The full-length ITS regions of all species in the DMCs were already present in an in-house database, from here on referred to as the IHEM database, prior to this study. The IHEM database originates from the BCCM/IHEM culture collection and represents 1,308 species from 376 genera selected from sequences deposited at the European Nucleotide Archive (https://www.ebi.ac.uk/ena) under the accession numbers OW982327 to OW988803. To create this database, these ITS regions were originally amplified using the primers ITS4 and ITS5 [28]. The polymerase chain reaction (PCR) was carried out with the following settings repeated for 35 cycles: 45 s of denaturation at 94 °C, 45 s of annealing at 94 °C, and 60 s of extension at 72 °C. Subsequently, the PCR amplicons were purified with ExoSAP-IT PCR Product Cleanup (Affymetrix, Santa Clara, CA, USA), and Sanger sequencing was performed on an ABI 3130xl Genetic Analyser (Applied Biosystems, Foster City, CA, USA) for sequences created before 2016 and on an ABI 3500 Genetic Analyzer (Applied Biosystems, Foster City, CA, USA) for sequences created from 2016 onwards, following the manufacturer’s instructions. Consensus sequences were generated by aligning and assembling the forward and reverse sequences using DNASTAR lasergene 10 (DNASTAR, Madison, WI, USA). Sanger sequences of both ITS1 and ITS2 were available for all species except for *Syncephalastrum racemosum* for which only the ITS2 region sequence was successfully amplified.

### ITS metabarcoding– Illumina sequencing

The primers ITS1catta (forward, ACCWGCGGARGGATCATTA) [29] and ITS2ngs (reverse, TTYRCKRCGTTCTTCATCG) [30] were selected to amplify the ITS1 region and the primers gITS7ngs (forward, GTGARTCATCRARTYTTTG) and ITS4ngsUni (reverse, CCTSCSCTTANTDATATGC) [31] were selected to amplify the ITS2 region.

#### Primer testing

Before DNA amplicon library preparation and Illumina sequencing, the amplification efficiency of the primers for ITS1 and ITS2 was tested. The two primer pairs were tested on the DNA of the individual 51 species by using the following PCR settings: denaturation at 95 °C during 30 s, followed by annealing at 55 °C for ITS1 and 47 °C for ITS2 during 30 s, and an extension step at 72 °C for 30 s. This was repeated for 30 cycles.

In order to ensure that the amplicon sizes could be covered by the short reads of Illumina, the length of the PCR amplicons was analysed by automated gel electrophoresis, using D1000 ScreenTapes on the 4200 TapeStation System (Agilent, Santa Clara, CA, USA) for each species and each DNA region, following the manufacturer’s instructions. The average length was 280 base pairs for ITS1 (range: 193–426 bp) and 283 base pairs for ITS2 (range: 199–565 bp) (Supplementary Table 2). No ITS1 amplicon of *Geotrichum candidum* was detected by the D1000 ScreenTape analysis (Supplementary Table 2).

#### DNA amplicon library Preparation and illumina sequencing

DNA amplicon libraries were created from the DMC input DNA, each amplifying either the ITS1 or ITS2 region, using the primers and PCR settings as elaborated above. The overhang adapter sequences, as specified by Illumina, were appended to these primers for compatibility with Illumina index and sequencing adapters. PCR and sequencing was performed as described in the amplicon metagenomics Sequencing Library preparation protocol of Illumina (part number 15044223 rev A.) on an Illumina MiSeq instrument (Illumina, Inc., San Diego, CA) with a 250-bp paired-end protocol (MiSeq v3 chemistry). All libraries were pooled into one MiSeq run, with 15% PhiX as internal control.

### Bioinformatics workflow

**Fig. 1:**
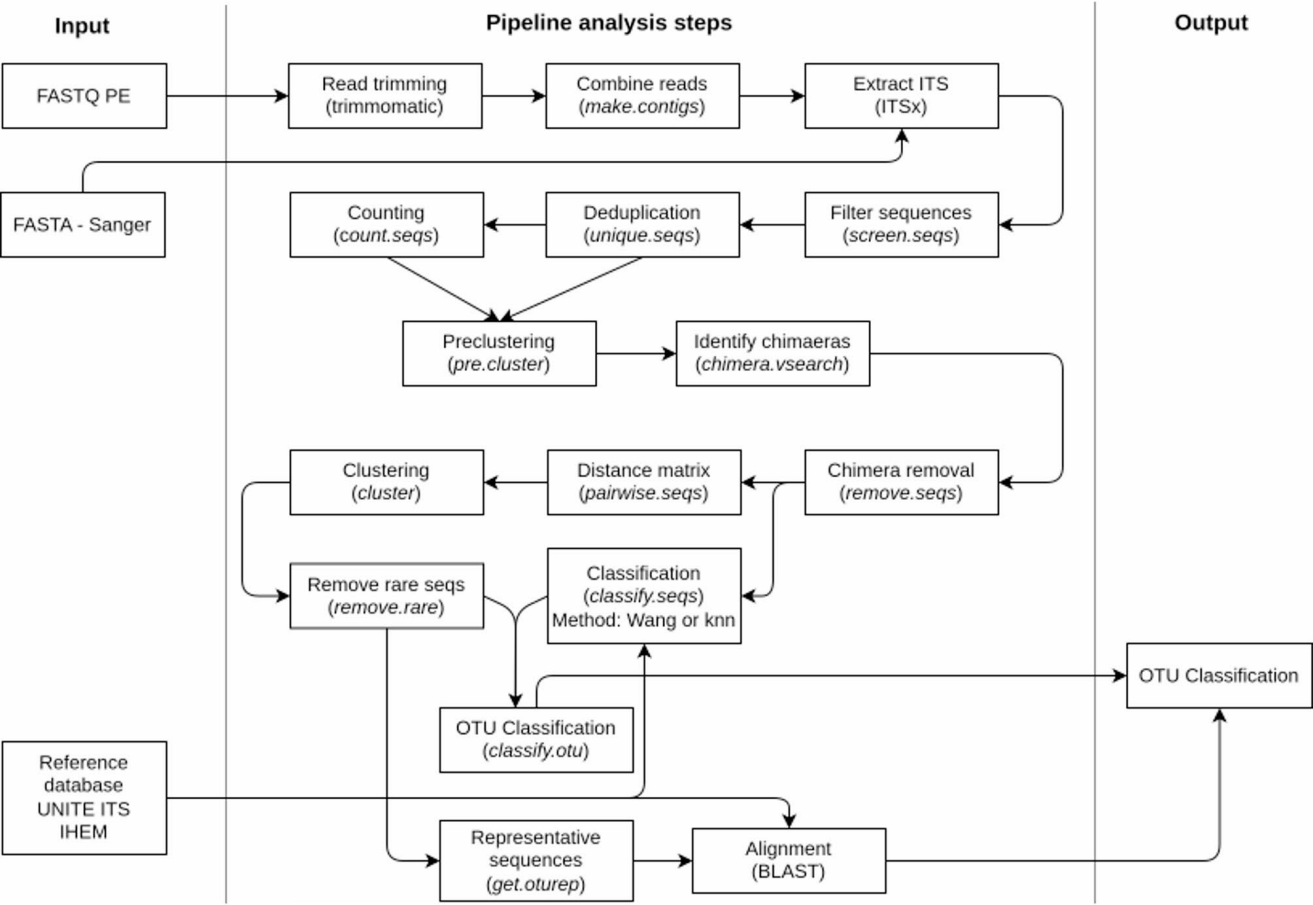
Overview of the bioinformatics pipeline used for the analysis. Input can be either paired-end FASTQ files (for NGS data) or FASTA files (for Sanger sequencing data). Names between brackets indicate the software used, with mothur functionality italicized

A pipeline for classification was created using Snakemake 5.4.0 [32] and Python 3.7. Many of the steps were performed using mothur v1.43.0 [24], an open-source bioinformatics tool suite widely employed by the microbial ecology community, supporting different sequencing technologies and having extensive documentation and online community support. A graphical overview of the pipeline can be found in Fig. 1.

#### ITS extraction

As both NGS paired-end sequences and Sanger sequences were available, the first steps of the pipeline depended on the input type. For NGS data, there were two additional steps in which the reads were first trimmed with Trimmomatic 0.36 [33] with the settings “LEADING:10”, “TRAILING:10”, “SLIDINGWINDOW:4:20”, and “MINLEN:10”. The surviving paired reads were then combined into contigs using the mothur functionality “make.contigs” resulting in a FASTA file. The remaining steps in the pipeline were identical for both the NGS and Sanger sequencing data. To remove the conserved parts of the sequences where the primers anneal, the sequences were processed with ITSx v1.1.2 [34] with the settings “-t F” to select the fungi profile and “-preserve T” to preserve the sequence headers so that only ITS1 or ITS2 sequences remained without primers, barcodes and flanking rRNA sequences.

#### Pre-processing

Mothur “screen.seqs” was then used to remove all contigs with more than ten ambiguity characters and/or homopolymers longer than ten bases, after which “unique.seqs” was used to only retain unique contigs. Highly abundant sequences are more likely to generate sequencing errors compared to rare sequences as more sequencing data is created. Consequently, rare sequences that are within two mismatches of highly abundant sequences, indicating that they likely contain sequencing errors, were subsequently removed with mothur “pre.cluster”. Next, chimeric sequences were identified and removed with mothur “chimera.vsearch” and “remove.seqs”, respectively.

#### Generating representative sequences

A matrix with pairwise distances was created with mothur “pairwise.seqs”. The resulting matrix was used to cluster the sequences in operational taxonomic units (OTUs) using mothur “cluster” with the default opticlust method and default cutoff of 0.03 [35]. OTUs consisting of a single sequence or less than 0.05% of total sequences were removed with mothur “remove.rare” as they likely represent sequencing errors and should be removed [36]. This last step was skipped when using Sanger sequences as these sequences are all singletons because they were pooled in-silico with an individual sequence for each species. For each OTU, a representative sequence, i.e., the most abundant sequence in each OTU, was obtained using mothur “get.oturep”.

#### Classification

After OTU creation, classification was done using BLAST or mothur with different databases and settings. Two databases were used: the IHEM database, containing 4,533 curated ITS sequences (date accessed 13/01/2020), and the UNITE database [37] v8.2 containing 84,387 ITS sequences.

For classification with BLAST, blastn 2.6.0 was used with default settings blasting the representative sequence for each OTU against the two databases. Only hits with at least 80% coverage and 95% identity were retained. Two different settings (strict and loose) were used to classify a BLAST hit as correct or incorrect. In the strict setting, a classification was only considered correct when there was a single top hit, based on E-value and percentage identity, that identified the expected species, and was considered as incorrect when that single top hit identified another species. When multiple equivalent top hits were found, i.e., hits with equal E-value and percentage identity, the OTU was considered unclassified. In the loose setting, a classification was considered correct when the expected species was present in the top BLAST equivalent hits. Both a single wrong hit and multiple equivalent hits not containing the target species were considered incorrect. In the latter case, a random species from those multiple equivalent hits was used for the tables listing the falsely classified species.

Classification with mothur was done using the “classify.seqs” and “classify.otu” functionality. For “classify.seqs”, both the Wang method [38] with a bootstrap cutoff of 80% and the k-nearest neighbour (knn) method taking only the single nearest neighbour were used. An important caveat of using the Wang method for classification, is that when the bootstrap cutoff at the given taxonomic level is not met, the OTU is not classified at that level and is thus not considered when counting the correct classifications, but instead is counted as unclassified.

Classifications with both mothur and BLAST were counted as unclassified when the name ended with “sp” or “unclassified”, indicating that the UNITE database did not give a classification down to the requested level or that mothur did not classify the OTU at the requested level, respectively. For both the BLAST and mothur classification, ITS1 and ITS2 were evaluated separately but a consensus approach was also implemented where a classification was only considered to be correct when both the ITS1 and ITS2 classification agreed on the same (correct) classification.

Fungi have other scientifically recognized subgeneric levels at which they can be classified besides the genus and species, namely the section, series and species complex [14]. As neither the IHEM nor UNITE databases include these levels by default for all taxa, a manual investigation of results was performed to evaluate whether certain wrong classifications at the species level that were correct at the genus level could also be correctly classified at the subgeneric level. More precisely, the identifications for which the top blast hits returned multiple species with equal E-value and percentage, were manually evaluated. If these species belonged to the same section, series or species complex as the original species in the DMC, they were considered correct.

### Performance evaluation

For all DMCs, the results of the different classifications were compared with the composition of the mock communities at both the species and genus levels. However, some species existed in the IHEM database that were not represented in the UNITE database. *Aspergillus chevalieri* (DMCs 5 and 33), *Aspergillus floridensis* (DMC 8), and *Apiospora montagnei* (DMCs 9, 12 and 27) are not present in the UNITE database so it is very likely that they will be identified as a closely related species. The reverse situation also occurred, i.e., a species in the UNITE database that was not present in the IHEM database, namely *Torula herbarum* (DMCs 6, 28), because the sequence entry had not yet been formally accepted by a specialist. In addition to missing species, it is also possible that databases can use a different name for the same species. In Supplementary Table 3, synonymous species names from the UNITE and IHEM databases are listed. A classification was considered to be correct when either synonym was identified.

To evaluate classification performance, a systematic approach was taken based on a previous study by calculating different relevant performance metrics and accompanying figures using Python [39]. An overview of the calculated metrics can be found in Table 1. Precision and recall were calculated from the number of OTUs that were correctly classified (i.e. a true positive), incorrectly classified (i.e., a false positive) and not identified (i.e., a false negative) based on the rules described above. To capture the balance between precision and recall in a single value, F1 scores were calculated. To support the interpretation of the different metrics, several graphs were created. A precision-recall plot was created per ITS region (i.e., ITS1, ITS2 and consensus) and taxonomic level (i.e., species and genus) for the classification containing data points for each combination of software & setting, and database. Additionally, separate graphs were created where precision, recall, and F1 score were plotted against the OTU abundance filter threshold. Cases where the metric could not be calculated due to the denominator being zero, were ignored in the calculation of the average and standard deviation when applicable. Total DNA of all species in each DMC was added in equal quantities and therefore, reads of each species in the DMC should theoretically be equally abundant, i.e., 20%, if no biases were introduced during library preparation and sequencing. To evaluate quantitative measurements, the read abundance of each correctly identified species was calculated and graphs showing the average and standard deviation of L1 distance values were made using Python. The L1 metric serves as a proxy for the distance estimations of a dataset compared to the expected abundances, ranging from 0 (i.e., identical abundance estimates) to 2 (i.e., completely different abundance estimates). For the L1 distance calculation, unclassified OTUs were not considered.

In all results, a nomenclature was used to indicate the combination of software, database and settings used. The format for this nomenclature was “DATABASE-SOFTWARE-SETTING” where DATABASE could be either IHEM or UNITE, and where SOFTWARE and SETTING could be either mothur combined with WANG or KNN, or BLAST combined with LOOSE or STRICT.

**Table 1:** Overview of performance metrics used in the validation, including their definition and equations

Metric	Definition	Formula
Precision	The likelihood that a classified species is truly present in the DMC.	Precision = TP/(TP + FP) * 100%
Recall	The likelihood that a species present in the DMC will be correctly identified.	Recall = TP/(TP + FN) * 100%
F1 score	Harmonic mean between the precision and the recall.	F1 = 2*TP/(2*TP + FP + FN)
L1 distance	The sum of the absolute differences between the observed abundances and the theoretical abundances in a sample. Unclassified reads were excluded in the calculations.	L1 = sum(|read abundance– theoretical abundance|)

*fp* false positives, *tp * true positives, *fn* false negatives

## Results

Two different scenarios were evaluated. In the first scenario, sequences obtained via Sanger sequencing of ITS amplicons were in silico mixed to resemble their respective species as denoted in Supplementary Table 1 to evaluate the theoretical maximum performance of metabarcoding resembling a situation where the ITS amplicon sequences are “perfect”. Sanger sequencing generate highly accurate consensus sequences and are therefore considered the “gold standard” in sequencing [40, 41]. All Sanger sequences of the species present in the different DMCs (Supplementary Table 1) were in silico mixed and then analysed using the described bioinformatics workflow (Fig. 1). Classification results of this analysis were used to emulate performance, i.e. the Sanger sequences were used as if it would have been possible to extract them from a DMC, even though this is impossible in a real metabarcoding analysis. This was done to set a baseline to compare the actually Illumina sequenced DMCs and evaluate whether issues with correct identifications were solely due to the Illumina sequencing process introducing errors such as primer bias and sequencing errors, and/or could also be attributed to the limited discriminatory power of ITS for certain genera and species. In the second scenario, the metabarcoding analysis was then performed by using Illumina sequencing of total DNA mixed for the respective species as denoted in Supplementary Table 1 to evaluate the potential effects of Illumina sequencing compared to the first scenario. For both scenarios, the effects of taxonomic level, ITS region, software, and database, were always evaluated. Subsequently, a manual curation of the results at the species and subgeneric levels was done, followed by evaluating the effect of abundance filtering on the performance of classification when using Illumina sequencing data. Lastly, it was evaluated how well abundances can be estimated from the Illumina sequencing data.

### Classification of in Silico DMCs

**Fig. 2:**
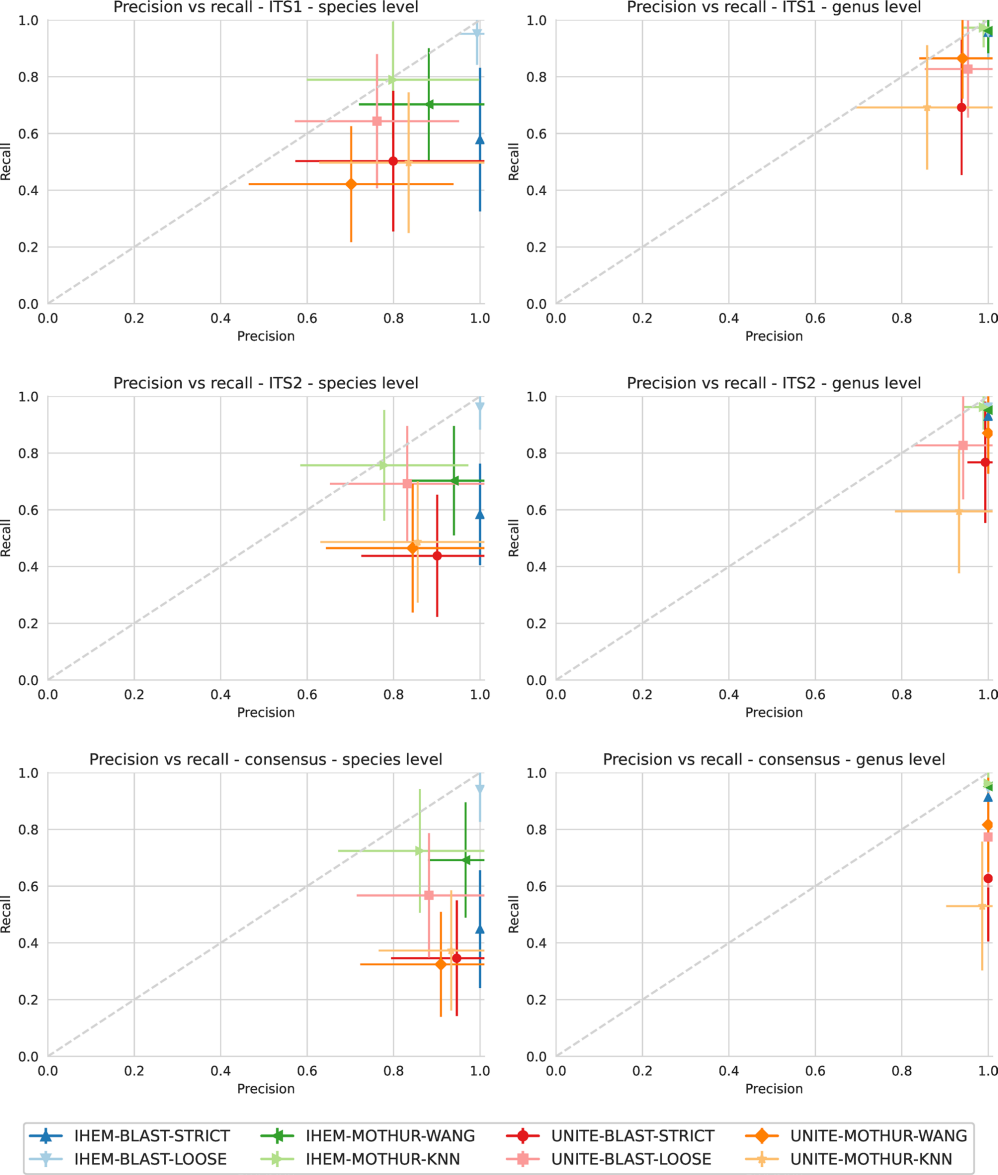
Precision-recall graphs per ITS region and taxonomic level used for classification of Sanger sequences, calculated for all considered combinations of database, software and setting. Average values (symbols) and standard deviation (error bars) for precision and recall are shown, calculated over all DMCs

The 50 available sequences for ITS1 clustered together in 50 OTUs (i.e., each sequence was unique). The 51 sequences available for ITS2 clustered in 46 OTUs, because the sequences of *Syncephalastrum racemosum* and *Thamnidium elegans* were removed during quality control due to a homopolymer stretch of 11 and 12 Ts, respectively (i.e., they were not considered). These homopolymer stretches could also be found in some of the sequences in the UNITE database. Additionally, *Penicillium aurantiogriseum*,* P. chrysogenum*, and *P. halotolerans* clustered together in a single OTU as well as *Cladosporium herbarum* species complex and *C. cladosporioides*. Figure 2 shows the precision-recall graphs for the combinations of different databases, software and method, grouped by taxonomic level and region used.

#### ITS1

At the species level, there was a large difference in precision and recall for the different combinations (i.e. software, databases and methods) (Fig. 2). IHEM-BLAST-LOOSE gave overall the best performance with a precision of 99.3% ± 4.1% and a recall of 95.1% ± 11.0%. Remarkably, IHEM-BLAST-STRICT had a precision of 100.0% ± 0.0% but exhibited a much lower recall of 57.8% ± 25.3% compared to using the loose setting. UNITE-MOTHUR-WANG gave overall the worst performance with a precision of 66.4% ± 28.1% and a recall of 42.2% ± 20.4%. Overall, using the IHEM database instead of the UNITE database resulted in higher performance, as did using BLAST instead of mothur. Over all combinations, precision was typically higher than recall.

At the genus level, there was much less variation in performance between the different combinations, and performance was generally higher compared to the species level, with all combinations having a mean precision > = 85.9% and a mean recall > = 69.2%. Variation in both precision and recall also decreased for combinations themselves (i.e., the different combinations displayed less variation in performance across all DMC samples, as evidenced by the error flags on Fig. 2). Combinations using the IHEM database overall outperformed combinations using the UNITE database, and several combinations (IHEM-MOTHUR-WANG, IHEM-BLAST-LOOSE and IHEM-BLAST-STRICT) even exhibited a precision of 100.0% ± 0.0% and relatively high recall (> 95.7% ± 9.6%) when using the IHEM database. IHEM-MOTHUR-KNN resulted in the highest recall of 97.3% ± 6.9%. Using IHEM-BLAST-LOOSE and IHEM-BLAST-STRICT resulted in a high recall of 95.7% ± 9.6%, indicating that the lower recall at the species level when using the strict setting, was caused by multiple equivalent BLAST hits for different species in the same genus. Using UNITE-MOTHUR-KNN resulted in the lowest performance with a precision of 85.9% ± 16.6% and a recall of 69.2% ± 21.9. Differences between mothur and BLAST when using the same database were much less pronounced than at the species level.

A full overview of the correctly classified species and the taxonomic level at which they were classified can be found in Supplementary Table 4. It was observed that some genera and species were identified using all combinations of software and settings when using the IHEM database, but were never or only once identified using the UNITE database. This is true for the sequences of *Scedosporium* and *Cladosporium* (for the *C. herbarum* sequence) that were never identified, those of *Apiospora*, *Geotrichum*, *Exophiala*, *Epicoccum*,* Penicilium* (for the *P. digitatum* sequence), and *Stachybotrys*, which were identified only once when using the UNITE database. A full overview of incorrect classifications can be found in Supplementary Table 5, containing 26 different species that were detected in one or more combinations. Most of these incorrect classifications were however from the correct genus, as only five different genera were incorrectly identified. most incorrect genera were only identified when using the UNITE database and only one incorrect genus, i.e., *Cyberlindnera*, was identified when using the IHEM database. F1 scores for each of the combinations can be found in Supplementary Fig. 1.

#### ITS2

At the species level, there was a large difference in precision and recall for the different combinations, with trends similar to ITS1 whereby using UNITE decreased performance compared to IHEM, and using mothur decreased performance compared to BLAST (Fig. 2). Notwithstanding, the precision of most combinations was higher compared to ITS1, whereas recall was largely in the same range (i.e., fewer false positives were generated when using ITS2 instead of ITS1). Using IHEM-BLAST-LOOSE resulted in the highest performance with a precision of 100.0% ± 0.0% and a recall of 96.2% ± 7.9%. Using UNITE-MOTHUR-KNN resulted in the lowest precision (77.8% ± 19.4%), and using UNITE-BLAST-STRICT resulted in the lowest recall (43.8% ± 21.5%).

At the genus level, similarly to ITS1, overall the variation in both precision and recall between combinations decreased, and performance was overall higher compared to the species level, with all combinations having a mean precision > = 93.2% and mean recall > = 59.5% (Fig. 2). Precision at the genus level for ITS2 was, hence for most combinations also equal or higher compared to precision at the genus level for ITS1. Variation in both precision and recall also decreased for the combinations themselves. Combinations using the IHEM database overall outperformed combinations using the UNITE database, and several combinations even exhibited a precision of 100.0% ± 0.0% and high mean recall ( > = 93.0%) when using the IHEM database. Using IHEM-BLAST-LOOSE, IHEM-BLAST-STRICT, and IHEM-MOTHUR-WANG resulted in a precision of 100.0% ± 0.0%, while this was only true for UNITE-MOTHUR-WANG when using the UNITE database. Using UNITE-MOTHUR-KNN for classification resulted in the lowest precision (93.2% ± 14.8%) and recall (59.5% ± 21.9%).

A full overview of the correctly classified species and the taxonomic level at which they were classified can be found in Supplementary Table 6. Similar to ITS1, some genera were only detected when using the IHEM database and never or only once when using the UNITE database. This was the case for *Gliomastix*, *Memnoniella*, *Scedosporium*, and *Cladosporium (*with the *C. herbarum* sequence) that were never identified, and *Apiospora*,* Purpureocillium*,* Exophiala*, and *Penicilium* (with the *P. digitatum* sequence) that were only identified once using the UNITE database. A full overview of incorrect classifications can be found in Supplementary Table 7, containing 20 different species that were detected in one or more combinations. Also similar to ITS1, only one incorrect genus (*Pithomyces*) was identified only when using the IHEM database, while the other incorrect genera were identified only when using the UNITE database.

#### Consensus approach

At the species level, precision was higher and recall lower for all combinations compared to using the separate ITS regions (Fig. 2). Using IHEM-BLAST-STRICT and IHEM-BLAST-LOOSE resulted in the highest precision of 100.0% ± 0.0% but with a recall of only 44.9% ± 20.8% for IHEM-BLAST-STRICT. The lowest precision overall was still relatively high with 86.1% ± 18.9% for IHEM-MOTHUR-KNN, while the lowest recall of 32.4% ± 18.5% for UNITE-MOTHUR-WANG was lower than any combination using the separate ITS regions.

At the genus level, all combinations showed a decrease in recall with the exception of IHEM-MOTHUR-KNN (96.2% ± 7.9%) and IHEM-MOTHUR-WANG (95.1% ± 8.7%), which had equal recall compared to using the ITS2 region. Again, the precision was equal to or higher compared to using the separate regions. The highest precision of 100.0% ± 0.0% was obtained with all combinations with the exception of UNITE-MOTHUR-KNN (98.6% ± 8.2%). Moreover, variation within individual combinations was also minimal.

A full overview of the correctly classified species and the taxonomic level at which they were classified can be found in Supplementary Table 8. Since the consensus approach requires a correct classification for both the ITS1 and the ITS2 sequences, the genera that were almost never correctly classified when using their respective ITS1 and ITS2 sequences and either the IHEM or the UNITE database, were also not correctly detected with the consensus approach. This was the case for the genera *Apiospora*,* Epicoccum*,* Exophiala*, *Geotrichum*, *Gliomastix*, *Memnoniella*, *Purpureocillium*, and *Scedosporium*. Conversely, Supplementary Table 9 lists the incorrect classifications and demonstrates that 11 incorrect species were detected at the species level and only one incorrect genus at the genus level.

### Classification of in vitro DMCs

#### ITS1

**Fig. 3:**
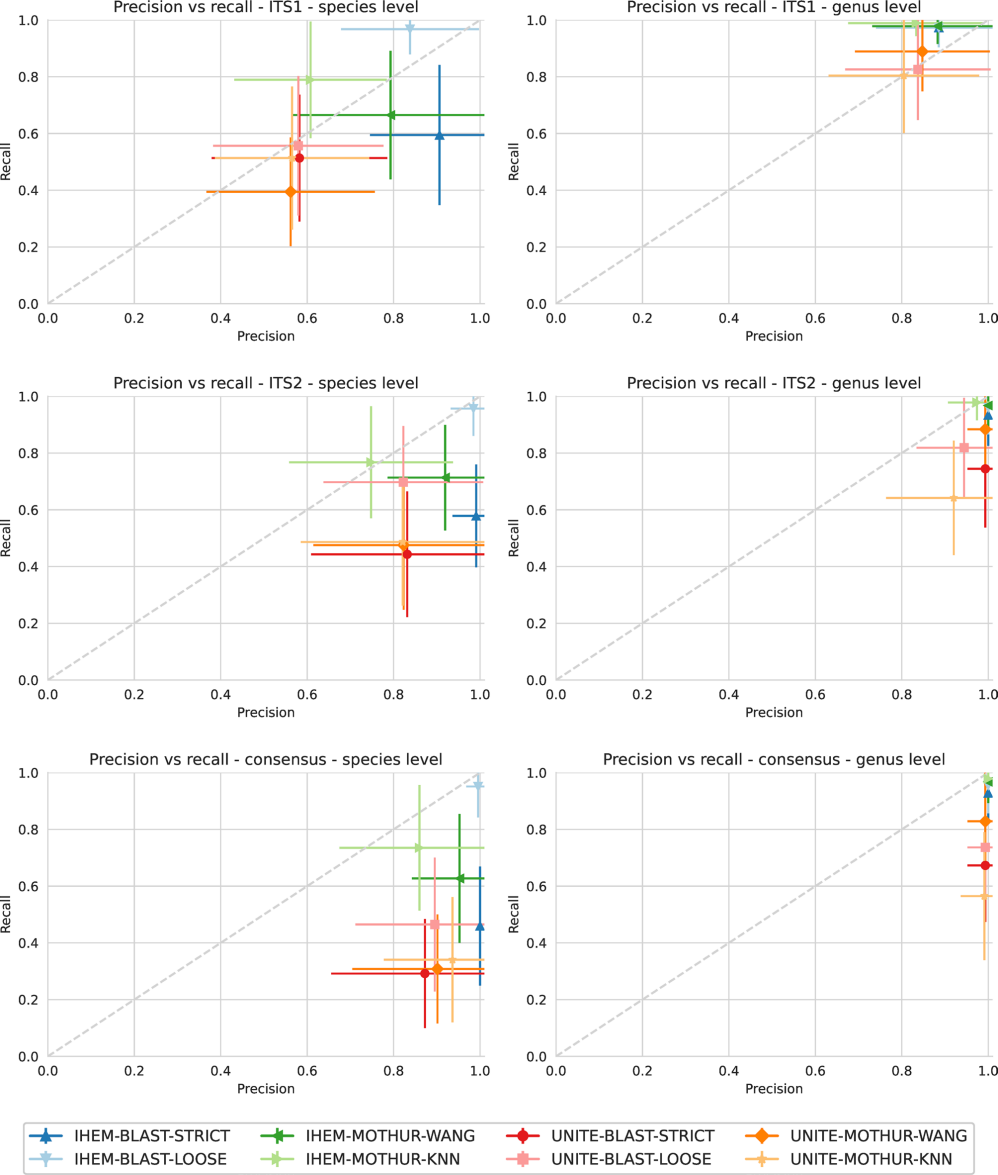
Precision-recall graphs per ITS region and taxonomic level used for classification of MiSeq data, calculated for all considered combinations of database, software and setting. Average values (symbols) and standard deviation (error bars) for precision and recall are shown, calculated over all DMCs

At the species level, the same combinations of database, software and setting displayed lower precision compared to results obtained via Sanger sequencing (Fig. 3). However, recall values varied for combinations between Sanger and Illumina sequencing with the recall being higher in eleven out of sixteen combinations for Illumina sequencing. Further investigation revealed that this effect was a result of the ITSx software not identifying the first bases of some of the Illumina sequences as belonging to the conserved parts of the 18S rRNA. Therefore, these bases remained in the Illumina output while they were cut when using Sanger sequences, as these included more conserved bases - due to different primers being used - allowing ITSx to identify them as 18S rRNA (results not shown) and cut them. The classification step would correctly classify the longer Illumina sequences at the species level, while this was not always the case for the shorter Sanger sequences. Species where this happened were present in multiple DMCs, e.g. *Cladosporium herbarum* was present in seven DMCs, resulting in higher mean recall for Illumina sequences for certain combinations. Similar to Sanger sequencing, especially the database had a profound effect on performance, with UNITE exhibiting lower performance compared to IHEM, followed by the software used with mothur exhibiting lower performance compared to BLAST. Using IHEM-BLAST-STRICT resulted in the highest precision of 90.6% ± 16.1%, whereas UNITE-MOTHUR-WANG had the lowest performance with a precision of 56.2% ± 19.5% and a recall of 39.5% ± 19.1%. These findings are illustrated in Supplementary Fig. 2, showing the F1 scores, demonstrating there exists a clear separation of the combinations using either the IHEM or the UNITE database. Combinations with the highest precision and recall were the same combinations that displayed the highest precision and recall when using Sanger sequencing.

At the genus level, both precision and recall increased markedly compared to species level for all combinations, and variation decreased for the individual combinations. Precision was lower compared to Sanger sequencing while recall was comparable with the exception of UNITE-MOTHUR-KNN and UNITE-BLAST-STRICT that had a higher gain in recall. The database had again the largest effect on performance, and the software had a much less pronounced effect on performance. Although several methods reached near-perfect recall, precision was lower compared to Sanger sequencing.

A full overview of the correctly classified species and the taxonomic level at which they were classified in each of the DMCs can be found in Supplementary Table 10. Similarly to Sanger sequencing, there were some species that were (almost) always identified when using the IHEM database, while they were never identified when using the UNITE database. This was true for the sequences of *Scedosporium* and *Geotrichum* that were never identified. Interestingly, the sequence of *Cladosporium herbarum* was not identified in five out of seven DMCs where it was present, while the correct genus was identified in the other two DMCs. Further investigation revealed that in the latter case, OTUs with a low number of reads comprising only 0.06% of the total number of reads were identified as *C. delicatulum* and thus led to a correct classification at the genus level, while the OTUs with more reads were classified as the false positive *Mycosphaerella tassiana*, as was also the case in the five DMCs where *C. herbarum* was not identified. An overview of all the false positives that were identified in each DMC can be found in Supplementary Table 11, demonstrating that the majority of false positives were found at the species level while only a few false positives were found at the genus level. Noteworthy, these false positives were not always the same ones as the ones identified with the in silico Sanger sequencing approach. Although there was some overlap, there were unique false positives found for both the Sanger and the Illumina sequences.

The technical replicates always gave identical classification results with only three exceptions where each time a false positive classification was introduced, most likely due to stochastic variations in the workflow at low abundance levels. For UNITE-BLAST-LOOSE and UNITE-BLAST-STRICT at the species level, there was an OTU with only 0.37% of the total number of reads that was wrongfully classified as *P. chrysogenum* in only one of the two replicates. For UNITE-MOTHUR-KNN, a correct classification of the genus *Penicillium* was made in only one of the two replicates and this for an OTU with only 0.07% of the reads.

#### ITS2

At the species level, similar to ITS1, the same combinations displayed lower precision compared to results obtained via Sanger sequencing, whereas the difference in recall varied between the different combinations (Fig. 3). Compared to ITS1 for Illumina, the precision was always higher, but for recall, there were again differences depending on the combination used. The combination IHEM-BLAST-STRICT resulted in a precision of 99.1% ± 5.5% but a much lower recall of 57.8% ± 18.1%, while BLAST-IHEM-LOOSE resulted in the highest recall of 95.7% ± 9.6% and a slightly lower precision of 98.5% ± 5.2% compared to the strict setting. UNITE-BLAST-STRICT resulted in the lowest recall of 44.3% ± 22.2% while IHEM-MOTHUR-KNN resulted in the lowest precision of 74.8% ± 19.0%. Supplementary Fig. 2 illustrates that a consistent separation of performance of the different combinations using either the IHEM or the UNITE database no longer existed, in contrast to using the ITS1 sequences. While using the IHEM database generally led to higher precision and recall, this was not the case for precision for IHEM-MOTHUR-KNN or for recall for IHEM-BLAST-STRICT. More precisely, IHEM-BLAST-STRICT exhibited a lower F1 score than UNITE-BLAST-LOOSE. Also, among the different software, there existed differences in precision and recall, with approximately the same trends as described for ITS1 sequences. The most noticeable difference with ITS1 was that using BLAST with the loose setting also clearly exhibited a higher F1 score compared to all other combinations of software and setting when using the UNITE database. In contrast, when using ITS1, the mean F1 score of UNITE-BLAST-LOOSE was comparable to the other combinations using the UNITE database.

At the genus level, the variation in both precision and recall between combinations decreased, and performance was overall higher compared to the species level. The highest precision of 100% ± 0.0% was observed for the combinations IHEM-BLAST-STRICT, IHEM-BLAST-LOOSE, and IHEM-MOTHUR-WANG, while the highest recall of 97.8% ± 6.3% was obtained with IHEM-MOTHUR-KNN. While the precision of the same combination of database, software, and setting was only slightly higher in two combinations when using Illumina sequencing compared to Sanger sequencing, for the recall, this was the case in ten out of the sixteen possible combinations. The lowest recall (64.2% ± 20.2%) and precision (92.0% ± 15.6%) were observed when using UNITE-MOTHUR-KNN. Whereas the mean F1 scores did no longer show a consistent separation between the combinations using the IHEM and the UNITE database at the species level, they still did so at the genus level.

A full overview of the correctly classified species and the taxonomic level at which they were classified in each of the DMCs can be found in Supplementary Table 12. Again, similarly to Sanger sequencing, there were some species that were never identified using the UNITE database, while they were always identified at least at the genus level when using the IHEM database. This was the case for the sequences of *Cladosporium (herbarum)*,* Gliomastix*,* Memnoniella*, and *Scedosporium.* Likewise, there was again some overlap between the false positives, but other false positives were unique to either Sanger or Illumina sequencing. An overview of the false positive identifications can be found in Supplementary Table 13.

For ITS2, the technical replicates always gave identical classification results, hence no additional false positives or negatives were introduced.

#### Consensus approach

At the species level, the precision and recall differed again depending on the different combinations, with some having higher and lower values compared to Sanger sequencing (Fig. 3). A perfect precision at the species level (100.0% ± 0.0%) was observed for IHEM-BLAST-STRICT, while the highest recall (95.1% ± 11.0%) was observed using IHEM-BLAST-LOOSE. The lowest precision, on the other hand, was observed when using IHEM-MOTHUR-KNN (86.0% ± 18.5%), while the lowest recall of only 29.2% ± 19.2% was observed when using UNITE-BLAST-STRICT. This is illustrated in Supplementary Fig. 2, where F1 scores were higher for IHEM compared to UNITE, but with a notably lower F1 score for IHEM-BLAST-STRICT, which was not as outspoken when using either ITS1 or ITS2. In general, the differences in performance followed a similar pattern as those displayed for ITS2, albeit with lower recall and higher precision.

At the genus level, a clear separation recall was observed with the combinations including the IHEM database scoring better, whereas the precision for all considered combinations was very similar and close to 100%. The consensus approach resulted in equal precision compared to using sequences generated by Sanger sequencing when using the IHEM database and near identical precision when using the UNITE database. Conversely, the mean recall was always higher except when using UNITE-BLAST-LOOSE. A perfect precision of 100.0% ± 0.0% was obtained for any combination using the IHEM database, while the lowest precision of 99.1% ± 5.5% was obtained for the combination UNITE-MOTHUR-KNN, which also had the lowest recall (56.5% ± 22.5%). The highest recall, on the other hand, was achieved using IHEM-MOTHUR-KNN, resulting in a recall of 97.8% ± 6.3%. This is also visible in Supplementary Fig. 2, where the F1 scores when using the IHEM database are much closer to 100%. Similarly to the consensus approach at the species level, the relative positions of the mean F1 scores mimic those when using ITS2 Illumina sequences, but with a lower recall.

A full overview of the correctly classified species and the taxonomic level at which they were classified in each of the DMCs can be found in Supplementary Table 14, while an overview of the false positive identifications can be found in Supplementary Table 15. Since the technical replicates for ITS2 did not introduce any additional false positives or negatives, the classification results using the consensus approach were also identical.

### Identification at subgeneric levels for in vitro DMCs

Results from DMCs subjected to metabarcoding and Illumina sequencing indicated that several combinations generated accurate classifications at the genus level, whereas at the species level, often drops in recall and precision were observed. According to the ICN (International Code of Nomenclature for algae, fungi and plants) [15], there exist two additional official ranks between the genus and the species, i.e., sections and series, although some genera are not formally subdivided into sections and/or series. In these cases, closely related taxa for which identification at the species level is difficult with ITS1 or ITS2 can be identified at the level of “species complex”. An inaccurate classification at the species level can therefore still be mitigated to some extent by an accurate one at either the section or series level. Since evaluation at these levels is hard to automate due to the absence of a standardized taxonomy, we took the best scoring method, IHEM-BLAST-LOOSE, and manually evaluated classification results at the section and series levels for both ITS1 and ITS2 for the Illumina data. This manual investigation of classification results indicated that many species could be correctly identified using an intermediate level between the species and the genus level (Supplementary Tables 16 and 17).

Both ITS1 and ITS2 displayed a lack of discriminatory power at the species level within the genera *Aspergillus*,* Penicillium*,* Cladosporium*,* Aureobasidium*,* Cephalotrichum* and *Fusarium*. However, the ITS regions were useful for accurate classifications up to series and/or section level in *Aspergillus* and *Penicillium*. Within these two genera, there were some differences between the discriminatory power of ITS1 versus ITS2. Out of the eight species belonging to *Aspergillus*, ITS1 could always identify *A. ochraceus*,* A. subalbidus*,* A. restrictus* and *A. ustus* to the species level and sometimes *A. fumigatus*, whereas ITS2 could only identify *A. fumigatus* and *A. restrictus*. The remaining species were correctly classified at the series or section level. Out of the seven tested *Penicillium* species, the ITS1 region could always identify *P. brevicompactum* and *P. digitatum*, and sometimes *P. corylophilum* and *P. halotolerans*, while the ITS2 region could always identify *P. brevicompactum*,* P. digitatum* and *P. citreonigrum* and sometimes *P. corylophilum*. The remaining species were correctly classified to series and/or section level. Species belonging to the genus *Cladosporium* could only be identified up to species complex, with the exception of *C. sphaerospermum*, which was always identified at the species level. Both ITS1 and ITS2 could not distinguish between *Cephalotrichum stemonitis* or its close relative *C. nanum*, and the identification was therefore retained at the *C. stemonitis* species complex. Within the genus *Fusarium*, one species was tested, namely *F. culmorum*. This species could not be identified at the species level, but it was correctly classified in the *Fusarium sambucinum* species complex. The ITS1 region could not unequivocally identify *Aureobasidium pullulans*, and the ITS2 region could only sometimes identify it. For this genus, it was not possible to assign a subgeneric classification, and the identification was left at *Aureobasidium sp.*, where appropriate.

Furthermore, some other differences were apparent between the ITS1 region and the ITS2 region. The ITS1 region had difficulties with species identification within the genera *Stachybotrys*,* Alternaria* and *Purpureocillium*. The identifications within *Stachybotrys* and *Alternaria* could be classified to a subgeneric level, as *Stachybotrys chartarum* species complex and *Alternaria* section *Alternata* respectively, but *Purpureocillium* could only be identified at the genus level. *Cryptococcus neoformans* could be correctly identified at the species level by ITS1, but ITS2 could only identify it up to the *C. neoformans/C. gattii* species complex. Lastly, neither *Torula herbarum* nor *Syncephalastrum racemosum* could be identified by either ITS regions.

In total, an additional 13 (ITS1) and 14 (ITS2) species that could not correctly be classified at the species level, could be classified to a subgeneric level.

### Abundance filtering to increase precision for in vitro DMCs

**Fig. 4:**
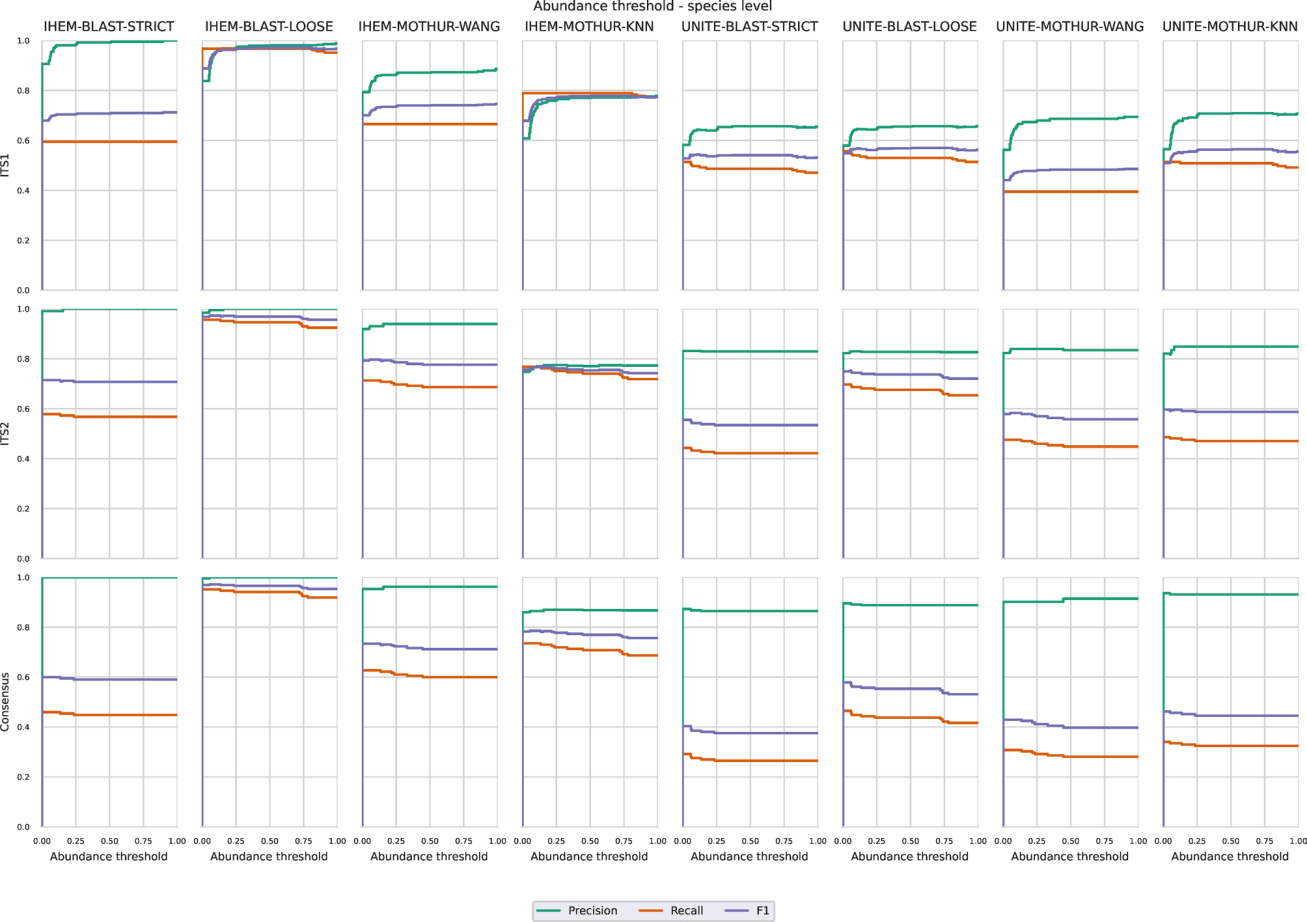
Plots showing the effect on the precision, recall, and F1 score when changing the abundance cutoff between 0.05% and 1% at species level. Graphs are for Illumina sequencing per ITS region used for classification and calculated over all DMCs for each combination of database, software and setting

**Fig. 5:**
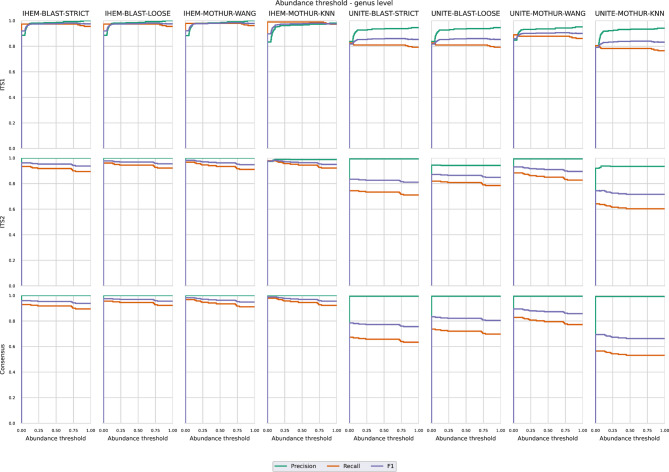
Plots showing the effect on the precision, recall and F1 score when changing the abundance cutoff between 0.05% and 1% at genus level. Graphs are for Illumina sequencing per ITS region used for classification and calculated over all DMCs for each combination of database, software and setting

For Illumina data, OTUs that had an abundance of less than 0.05% were filtered out in the workflow (Fig. 1), since these were likely sequencing errors resulting in false positive predictions (note that for the in silico mixed Sanger sequences no abundance information was available). Since increasing this threshold to higher abundances could further reduce the number of false positives and increase precision, albeit at a cost in recall, we investigated more strict abundance filtering thresholds. Figures 4 and 5 illustrate the effect of varying the abundance filtering thresholds from 0 to 1% in increments of 0.05% at the species and genus level, respectively, on the mean precision, recall, and F1 scores. For ITS1, adjusting the OTU abundance cutoff resulted in a sometimes large increase in precision while having little to no impact on the recall, consequently overall increasing F1 values. More specifically, increasing the abundance cutoff to 0.5% instead of the default cutoff of 0.05% always increased the precision and F1 scores while having no penalty in recall when using the IHEM database at both the species and genus levels. When using the UNITE database, the recall always had a small penalty for all combinations at both the species and genus levels with the exception of UNITE-MOTHUR-WANG at the species level. For ITS2, however, recall values were affected more strongly when increasing the abundance filtering cutoffs despite having only a limited positive effect on precision. Consequently, increasing the abundance cutoff to 0.5% resulted in a decrease in recall for all possible combinations at both the species and genus levels. Moreover, the negative effect on recall was more pronounced compared to the positive effect on precision, as F1-scores also overall decreased, with the exception of a small increase when using IHEM-BLAST-LOOSE. In general, a cutoff of 0.1% when using ITS2 did increase the F1 scores for most methods, as the decrease in recall was compensated by a higher precision. For the consensus approach, similar to ITS2, increasing the abundance filtering threshold had a limited positive effect on precision, whilst having a more pronounced negative effect on recall and therefore exhibiting decreasing F1 scores at both the species and genus levels at cutoffs above 0.1%. Based on these results, a cutoff of 0.5% for ITS1, and of 0.1% for ITS2 and the consensus approach, are recommended for increasing precision.

### Abundance estimations for in vitro DMCs

**Fig. 6:**
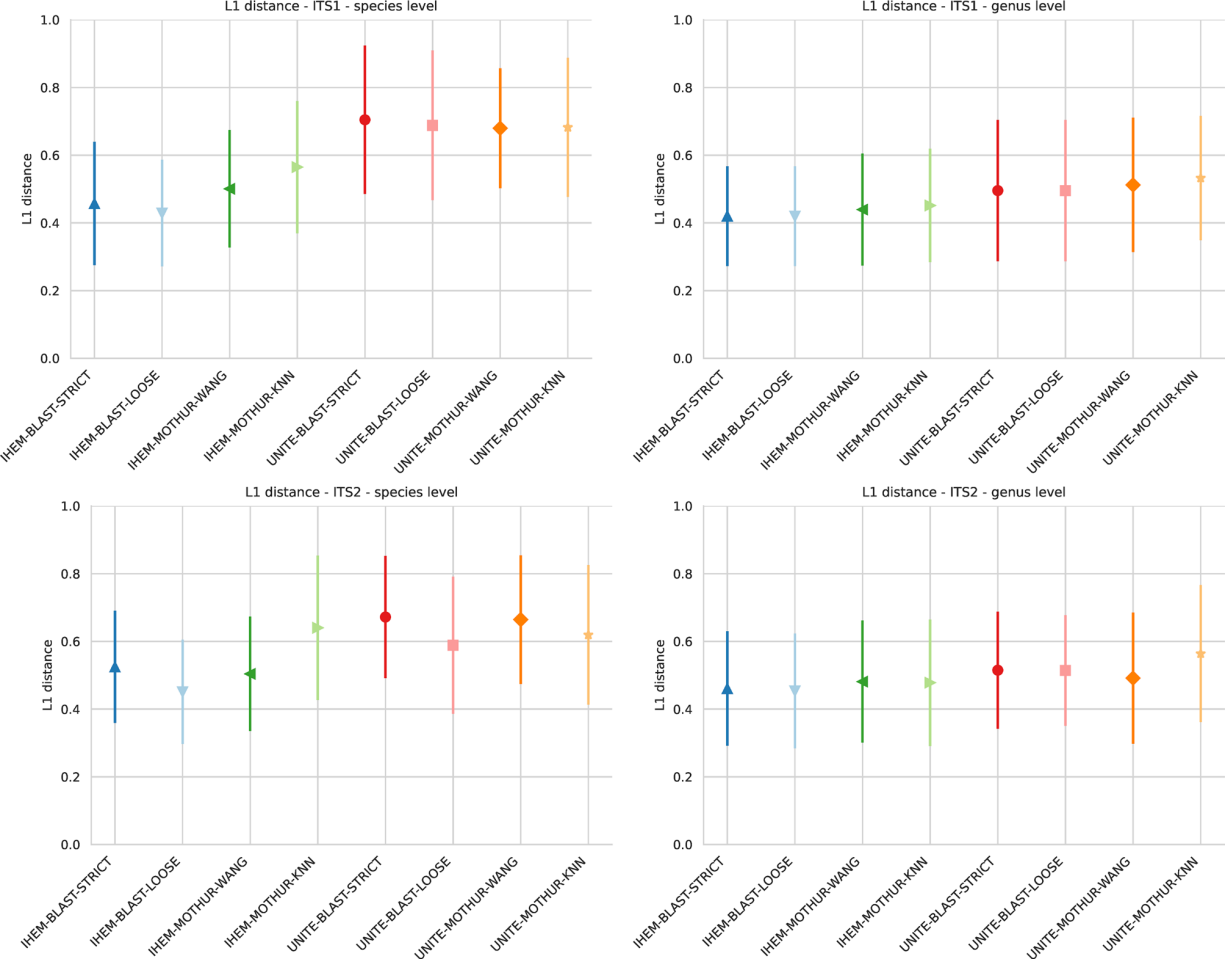
L1 distances per ITS region and taxonomic level used for classification of reads generated with Illumina sequencing, calculated for all considered combinations of database, software and setting. Average values (symbols) and standard deviation (error bars) for the L1 distances are shown, calculated over all DMCs. A score of 0.0 represents perfectly predicted abundances and species while a score of 2.0 represents that only abundances for incorrectly classified species were predicted

As the DNA of all species was added in equal quantities, i.e. 20% for each species, L1 distances were calculated to verify whether the sequencing data could be used to estimate abundances (note that for the in silico mixed Sanger sequences, no abundance information was available). For these analyses, the sequence abundance was calculated [42] as there can be variability in both genome size and ITS copy number for each of the species in the DMCs. Converting these sequence abundances to taxonomic abundances is impossible, as the exact ITS copy numbers for the species used in the DMCs are not available. Figure 6 provides an overview of the average L1 distances and the standard deviation for all combinations for both ITS1 and ITS2 at the species and genus levels. Note that no L1 distances could be calculated for the consensus approach, as this is a combination of both ITS1 and ITS2 that were sequenced independently, and it consequently was impossible to extract a combined read abundance. At the species level, an effect of the database on abundance estimations was observed where the L1 distances tended to be greater when using the UNITE database for ITS1, which was less marked for ITS2. For both markers however, IHEM-MOTHUR-KNN exhibited higher L1 mean values compared to the other combinations using the IHEM database; whereas this effect was not observed when using the UNITE database. For the same combination of software, database and setting, there was high variation in L1 distances between the DMCs observed, as evidenced by the large error flags. At the genus level, the L1 scores were much more similar between the different combinations for both ITS1 and ITS2. Large variation remained between the different DMCs for the eight different combinations individually. Supplementary Figs. 3–6 show histograms of the read abundance of correctly identified species over all DMCs with each combination of ITS region, taxonomic level and software setting grouped per software and database. Supplementary Tables 18–21 shows how many times the abundance of a correctly identified species was over- or underestimated with more than 5% over all DMCs. The abundances for some species or genera were consistently over- or underestimated while for others, the results varied between the different combinations of ITS region, database, software and setting. In general, using the Illumina data to estimate abundances was unreliable and the magnitude of deviation from the true abundance depended on the ITS region, the composition of the DMCs, the taxonomic level, database, software, and setting used for the analysis.

## Discussion

In this study, we investigated the performance of ITS1 and ITS2 metabarcoding when using short-read Illumina sequencing for identifying fungal species in complex samples, using a series of 37 DMCs with known composition, two ITS reference databases, and different commonly used software and settings. We employed a framework for systematic evaluation that allowed us to evaluate the effects of these factors on classification performance [39]. Our results demonstrate that large differences exist, primarily due to the underlying database and taxonomic level at which the classification was evaluated, but also to the software and settings used, which ITS region was sequenced and the Illumina sequencing itself.

We evaluated classification performance using ITS1 and ITS2 sequences generated by both Sanger and Illumina sequencing. Sanger sequencing is impractical for direct sequencing of mixed communities, as it cannot handle samples containing multiple species and/or those that are challenging to cultivate. However, Sanger sequencing represents the current “gold standard” for fungal identification, generating long contiguous sequences that are highly accurate [40, 41]. Therefore, we created each DMC in silico from the corresponding Sanger sequences as if it would have been possible to extract them individually from each DMC to establish a baseline for the performance of fungal identification to compare the actual DMCs undergoing Illumina sequencing. The latter generates short reads that need to be bioinformatically analysed to obtain OTUs, during which potentially multiple biases and artefacts, due to differences in e.g. sequencing depth, sequencing errors, sequence filtering or clustering thresholds, can be introduced [43]. Hence, an evaluation of classification performance between Sanger and Illumina sequencing allowed us to evaluate to what extent classification performance is affected by the short-read sequencing technology itself. Evaluation of classification performance with Sanger sequencing for both ITS1 and ITS2 demonstrated that for the different combinations, performance was never perfect (Fig. 2). Even though Sanger sequencing is considered the gold standard [40, 41], our analysis demonstrated suboptimal performance, indicating some limitations in the discriminatory power of ITS1 and ITS2, in line with previously reported findings [4]. Evaluation of the same combinations on Illumina data (Fig. 3), demonstrated that the mean precision was lower for most combinations compared to Sanger sequencing, indicating that Illumina sequencing resulted in more false positive identifications, most likely due to the fact that short reads first need to be translated into OTUs. The mean recall was not consistently lower or higher for Illumina compared to Sanger sequencing. For ITS1 at the species level, a higher recall was sometimes observed for Illumina sequencing because the ITSx software did not identify the first bases of the sequences as belonging to the 18S rRNA, which it did when using Sanger sequences that were created using different primers, thereby affecting the downstream classification. However, as recall was otherwise not heavily affected, Illumina did not lead to the inclusion of more false negatives and the recall was comparable to Sanger sequencing. Comparing the two technical replicates for the Illumina technology revealed that there were only a few differences between the two samples due to stochastic variation, only when using ITS1 for three combinations using the UNITE database for OTUs with a very low number of reads. These differences were all additional false positives, and no additional false negatives were encountered, indicating that the sequencing process itself can be replicated without major issues.

Illumina sequencing was influenced by different factors: the taxonomic level (genus or species), marker (ITS1, ITS2, or consensus), database (IHEM or UNITE), and method & setting (BLAST or mothur, each with custom settings). The taxonomic level had the largest effect on classification performance. Classification at the genus level always resulted in higher precision and recall compared to classifications at the species level, irrespective of the considered combination, with the sole exception of IHEM-BLAST-STRICT for ITS1 where the precision was slightly lower, i.e. 88.6% ± 14.5% at the genus level versus 90.6% ± 16.1% at the species level (see below). Consequently, F1 scores were always higher at the genus level, indicating that the overall accuracy still increased in line with observations from other studies [44–46]. We moreover found that manual curation of results for identification at a subgeneric level can offset this loss in performance at the species level to a certain extent. The recuperation of as much taxonomic information as possible is relevant, notably to uncover the distribution patterns of fungal groups in various ecosystems [47, 48]. However, this subdivision at subgeneric levels has to be done manually, is time-consuming and requires expert mycological knowledge. This would hence be only reasonable to perform when a limited number of samples need to be processed. Because of the ever-increasing information on phylogenies and relationships between fungal organisms, it is difficult to construct an accurate, curated, and publicly accessible database containing this information on section or series subdivisions. For instance, for fungal taxonomy, the comprehensive NCBI taxonomy browser generally only shows information at the genus and species levels, and only occasionally information on species complexes is provided.

Besides the taxonomic level, the employed reference database had a profound effect on performance. F1 scores were consistently higher when using IHEM instead of UNITE except IHEM-BLAST-STRICT that had a slightly lower mean F1 score than UNITE-BLAST-LOOSE for ITS2 at the species level. Database choice could also have a different effect when using different ITS regions. For instance, IHEM-BLAST-LOOSE resulted in comparable recall for the consensus approach using either ITS1 or ITS2 at both the species and genus levels, but this was not the case when using UNITE-BLAST-LOOSE, which exhibited lower recall for the consensus approach compared to either ITS1 or ITS2. This was explained by the fact that, depending on the species, false negative results were higher when using either ITS1 or ITS2 and the UNITE database. Another noteworthy observation was that there existed a larger difference in precision between IHEM-BLAST-STRICT and IHEM-BLAST-LOOSE for ITS1 at the species level, which was not the case for ITS2 or at the genus level. This indicates that when using BLAST and the IHEM database, there was often more than one equivalent incorrect hit, while the same did not occur with the UNITE database, ITS2, nor at the genus level. The difference in precision and recall between IHEM and UNITE can potentially be caused by wrong or incomplete annotations in the database. Since both IHEM and UNITE are curated, the probability of containing wrong annotations is likely limited. However, because of the rapid changes in fungal taxonomy, there can be incongruences in the naming between different databases. The annotation can also exert an influence due to sequences for which no classification is available at the genus and/or species levels. There were 43,716 and 22,456 entries in the UNITE database version we used, for which no classifications were available at the species and genus level respectively, whereas in the IHEM database, all sequences were annotated down to the species level. Database size is another likely cause for the observed differences between IHEM and UNITE. For instance, as the Wang method implemented in mothur filters the results based on a bootstrap value, results should be more robust compared to the knn method that simply picks the closest sequence. As the database size increases, the chance of finding closely related sequences that are not from the target species also increases, leading to decreasing bootstrap values and subsequent filtering out correct identifications when dropping below this threshold. This effect is clearly visible where the number of unclassified OTUs increased markedly when using the UNITE database instead of the IHEM database combined with mothur and the Wang method (Supplementary Table 22), and was most pronounced at the genus level. It is likely that the IHEM database would suffer from the same effects if it were to reach a similar size to the UNITE database. On the other hand, the larger UNITE database contains more species, which could lead to the identification of species not present in the IHEM database. Several studies have found that having a smaller, specific and dedicated database for taxonomic classifications can increase the accuracy of the analysis and that larger databases can lead to loss of resolution [49–54]. For guidance of DMCs with unknown composition, this offers the opportunity to increase performance by using a specific database that fits the application scope by containing a range of species most likely to be encountered. For the species considered in this study, i.e., species commonly found in an indoor environment, the IHEM database is very well suited.

The choice of ITS region also had a marked effect on classification performance. Both at the genus and species level, ITS2 typically displayed higher precision than ITS1 with comparable or slightly lower recall, i.e., the number of false positive predictions was lower, whereas the number of false negative predictions was in the same range, which was also reflected in the higher F1-scores for ITS2. Interestingly, the consensus approach also displayed high precision but was affected by reduced recall, indicating that considering both markers also decreased false positives but induced more false negatives because some species are more likely to be correctly classified using either ITS1 or ITS2. The consensus approach may therefore be too strict, unless when avoiding false positive predictions is highly relevant for the intended application. Despite ITS2 displaying higher precision with comparable recall compared to ITS1, the latter still can be a relevant marker to use because both ITS1 and ITS2 missed different species identifications, whereby ITS1 was able to identify certain species that were missed by ITS2 (and vice versa). For instance, *Purpureocillium lilacinum* could only be identified by ITS1 using the UNITE database. Conversely, *Geotrichum candidum* could only be identified by ITS2 using the UNITE database. A limitation of using both ITS1 and ITS2 is that it is impossible to know whether the ITS1 and ITS2 sequences come from the same organism, and especially in highly complex samples, this could lead to spurious classifications. Overall, ITS1 and ITS2 appeared quite robust in identifying the majority of the species in the DMCs, and our results are in line with previous findings on the performance of ITS in several fungal groups. Schoch et al. [4] reported an effective species discrimination of more than 70% when using the whole ITS region, but a lower discriminatory power of ITS has been described for some of the genera used in our study, e.g., *Aspergillus*,* Penicillium*,* Aureobasidium*,* Cephalotrichum*,* Fusarium* and *Cladosporium* [5, 55–58] but also for other genera [13, 59, 60], hinting that some of the misclassifications come from species that show limited sequence divergence between their ITS sequences.

The software and its settings also influenced results. BLAST typically resulted in better performance compared to mothur, although the effect could differ substantially depending on the ITS region, employed database, and taxonomic level considered. This effect was especially noticeable at the species level for both ITS1 and ITS2, where IHEM-BLAST-LOOSE yielded the highest performance. However, this combination has a significant limitation, as the correct species is often included in a list of other species that appear as equivalent BLAST hits. Therefore, an additional examination by an expert is required. This detection method was included in our evaluation because it mimics current procedures whereby researchers typically BLAST obtained Sanger sequences and manually decide on potentially present species based on the top BLAST hit results, for which often multiple species are present, which is then followed by a deeper investigation [61–64]. Enforcing the strict setting, whereby only one top hit containing the correct species is considered, immediately incurred a heavy penalty in recall at only a small gain in precision. Consequently, using mothur with the IHEM database often resulted in F1 scores that were comparable to using BLAST with strict settings. The same trends were apparent when using the UNITE database with BLAST and mothur, albeit less pronounced. This implies that when no manual curation by an expert is possible and/or the context of the sample is unclear, there is no discernible advantage of using either BLAST with strict settings compared to mothur. MOTHUR-WANG displayed a very modest performance advantage compared to the knn method for both ITS1 and ITS2. At the genus level, the strong performance advantage of BLAST-LOOSE was no longer present, and all methods exhibited similar F1 scores when using the IHEM database, while MOTHUR-WANG had a small performance advantage compared to the other combinations when using the UNITE database. Consequently, it is hard to provide a general recommendation on which software to use, as this should take into account the possibility of performing curation of the results, the employed database, the considered taxonomic level, and the relevance of precision and recall to the research question. Notwithstanding, our results indicate that overall BLAST-LOOSE is a good detection method for both the IHEM and UNITE databases when expert curation can be provided, whereas MOTHUR-WANG is a good detection method for both databases when this option is not available.

An inverse relationship between recall and precision generally exists within classification, and precision can often be improved by applying more strict abundance filtering thresholds [65, 66]. In our study, a default abundance filtering threshold of 0.05% was used for all detection methods, i.e. OTUs were filtered out with a read abundance ≤ 0.05%. Our results (Figs. 4 and 5) confirmed that increasing this threshold increased precision, but the effect on recall varied. For ITS1, increasing this cutoff to 0.5% considerably increased precision for most methods without an effect on recall, so that the overall F1 scores increased. For ITS2, however, increasing this cutoff to 0.5% did increase precision but at a substantial cost in recall, so that the overall F1 scores decreased. Setting the threshold at 0.1% did increase the F1 scores for most methods, with only a small decrease for UNITE-MOTHUR-KNN, UNITE-BLAST-LOOSE and UNITE-BLAST-STRICT due to a slightly lower recall. One explanation for this drop in recall at low abundance cutoff values is that sometimes OTUs representing a small number of reads were classified correctly, while an OTU representing more but very similar sequences was classified incorrectly. This finding is remarkable, since increasing this threshold would theoretically be expected to pose a problem for species present at very low abundances, whereas they were always present in equal abundances of 20% in every DMC. Our results demonstrated that considerable variation in the percentage of reads existed and were classified as each species. Using the L1 metric as a proxy for approximating the real relative abundance within each DMC, we found that the experimental abundances could often deviate substantially depending on the considered combination (Fig. 6). At the genus level, mean L1 distances were slightly lower when using IHEM compared to UNITE. At the species level, the same trend was more pronounced, with a larger difference between the mean values when using IHEM compared to UNITE, with the sole exception of IHEM-MOTHUR-KNN that had a higher mean L1 distance than UNITE-BLAST-LOOSE and UNITE-MOTHUR-KNN when using ITS2. Further research is needed to determine the causes for the differences in abundance and the limit of detection of a species. One potential explanation may be PCR amplification bias between the different species present in the same DMC, while another could be the variation in ITS copy numbers across fungal species, potentially causing an overrepresentation of high-copy-number species [16, 31, 37, 67].

Limitations of our study include the relatively simple composition of DMCs with only five species present per DMC, with equal abundances, which were pooled in vitro, rendering them a simplification of real samples, where other biases may be introduced. Although unbalanced mixes would better represent real samples, our results show that the estimated abundances from the read counts can differ greatly, possibly resulting in certain species remaining undetected in these unbalanced mixes when underestimated. Additionally, species were selected to be representative of indoor samples and results could therefore differ when analysing species from different environments, such as soil samples. Furthermore, our analysis began with DNA rather than cells, meaning that biases introduced during DNA extraction were not addressed, which could impact results when starting from real environmental samples. Notwithstanding, in the absence of other studies or evidence on the performance of classification methods for other types of samples, our results can serve as a proxy to guide the marker selection and bioinformatics analysis for other application scopes. Another limitation is that we employed a limited set of detection methods and combinations, while other methods and workflows are used in the field [45, 68–70]. We opted to use mothur and OTU-based analysis to evaluate a (semi-automated) workflow, but other options exist and are commonly used such as QIIME and/or amplicon sequence variant-based analysis [71–73], which can potentially result in increased performance although the added value thereof remains unclear [74, 75]. A systematic benchmarking of different metabarcoding software packages was however not in scope for our study. However, the selected methods consider a robust set of potential approaches, as the BLAST-based approaches mimic the traditional hands-on investigation by expert scientists [61–64], whereas mothur is considered a standard in the field for more automated processing [76]. Moreover, the UNITE database is considered a standard and high-quality curated database for ITS containing a very broad representation of the fungal kingdom [77], whereas the IHEM database represents a high-quality curated database well-adapted for indoor fungi. Our extensive evaluation also did not consider all factors which may potentially affect classification. For instance, the choice of primers is an important factor influencing identification, as some primers can be biased against several groups of fungi due to the intragenomic variation of the ITS region [31, 78, 79], and it can also impact the fungi that are recovered from a sample [80]. Other factors such as environmental variables, DNA extraction method, and PCR conditions could also influence our results when using real environmental samples [26, 43, 80].

## Conclusions

The choice of ITS region, taxonomic level, database, software, and settings all influenced classification performance and they have to be carefully considered and adapted to fit the application scope. In particular, even when using Sanger sequencing to set a theoretical baseline to compare Illumina against, suboptimal performance was observed due to the low discriminatory power of the ITS region for certain species and genera. With Illumina sequencing, the precision typically decreased while the recall was comparable. Performance was best at the genus level, while at the species level, results were more diverse but suffered from lower precision and recall. Additionally, expert knowledge was required to interpret results to gain more information from the data. Although the IHEM database performed better than the UNITE database, this effect resulted from a lower number of entries, for which full annotations were available down to the species level. ITS2 typically resulted in a slightly better precision and similar recall compared to ITS1. BLAST-LOOSE resulted in the best performance overall but requires expert knowledge to correctly interpret classifications. MOTHUR-WANG tended to result in better performance when using mothur, but this was dependent on the aforementioned factors. Abundance estimations using read counts to estimate the relative abundance of species overall performed poorly, and abundance filtering improved performance for ITS1 but led to the loss of recall for ITS2. Given that the DMCs always contained five species at equal abundances and that sequence abundance estimates varied broadly, abundance filtering could impair a strong cost in recall when species would be present at low abundances in samples. Notwithstanding, our results demonstrated that Illumina sequencing of ITS1 or ITS2, irrespective of database, software and settings, can be used to robustly predict the fungal composition of unknown samples at the genus level. At the species level, both false negative and false positive findings become problematic, and require careful consideration of the employed region, database, software, and setting, as well as the limited discriminatory power of ITS for certain species. Newer sequencing technologies, such as in particular long-read sequencing as offered by Oxford Nanopore Technologies, may offer promising alternatives compared to more traditional short-read Illumina sequencing, as long-read sequencing allows for sequencing of the entire ITS region. This could potentially increase the performance by leveraging the entire sequence, but would require adaptation of the bioinformatics analyses to work with these long sequences. Also, the higher error rate of long read sequencing could still negatively affect performance as observed for 16 S rRNA sequencing [81] even though recent advances show a substantial reduction in error rates and accompanying increase in accuracy [82–85].

## Electronic supplementary material

Below is the link to the electronic supplementary material.

Supplementary Material 1

## Data Availability

The datasets supporting the conclusions of this study have been deposited in the NCBI Sequence Read Archive under BioProject number PRJNA814881.
