# Supplementary material for: Investigating fungal diversity through metabarcoding for environmental samples: assessment of ITS1 and ITS2 Illumina sequencing using multiple defined mock communities with different classification methods and reference databases
Source: BMC Genomics. 2025 Aug 6;26:729. doi: 10.1186/s12864-025-11917-y (PMC12329927; doi:10.1186/s12864-025-11917-y)
Supplement: Supplementary file 1 — Supplementary Material 1 [file 12864_2025_11917_MOESM1_ESM.docx]

**Supplementary material**

# Tables

Table 1: Overview of the composition of the defined mock communities (DMCs). Each DMC consisted out of 5 species in total, for which DNA was added in equal quantities. All DMCs contained species belonging to five different genera except for DMCs 1, 18, 19, and 34, which contained four different genera. DMCs 36 and 37 were the same and served as technical replicates.

| **1** | **2** | **3** | **4** |
| --- | --- | --- | --- |
| *Aspergillus versicolor* | *Aspergillus versicolor* | *Cladosporium cladosporioides* | *Cladosporium sphaerospermum* |
| *Aspergillus fumigatus* | *Stachybotrys chartarum* | *Aspergillus ochraceus* | *Penicillium chrysogenum* |
| *Cladosporium sphaerospermum* | *Aureobasidium pullulans* | *Chaetomium globosum* | *Aspergillus fumigatus* |
| *Stachybotrys chartarum* | *Cladosporium herbarum* | *Rhizopus arrhizus* | *Chaetomium globosum* |
| *Penicillium chrysogenum* | *Penicillium aurantiogriseum* | *Saccharomyces cerevisiae* | *Memnoniella echinata* |
| **5** | **6** | **7** | **8** |
| *Cladosporium cladosporioides* | *Torula herbarum* | *Cladosporium herbarum* | *Cladosporium sphaerospermum* |
| *Aspergillus chevalieri* | *Aspergillus subalbidus* | *Byssochlamys spectabilis* | *Aspergillus floridensis* |
| *Cephalotrichum stemonitis* | *Saccharomyces cerevisiae* | *Scopulariopsis brevicaulis* | *Beauveria bassiana* |
| *Scedosporium apiospermum* | *Exophiala jeanselmei* | *Apiospora montagnei* | *Cephalotrichum stemonitis* |
| *Syncephalastrum racemosum* | *Sporobolomyces roseus* | *Exophiala jeanselmei* | *Syncephalastrum racemosum* |
| **9** | **10** | **11** | **12** |
| *Cladosporium cladosporioides* | *Cladosporium sphaerospermum* | *Cladosporium sphaerospermum* | *Cladosporium herbarum* |
| *Aspergillus restrictus* | *Purpureocillium lilacinum* | *Aspergillus restrictus* | *Aspergillus ustus* |
| *Rhizopus arrhizus* | *Rhodotorula mucilaginosa* | *Cephalotrichum stemonitis* | *Penicillium brevicompactum* |
| *Exophiala jeanselmei* | *Alternaria alternata* | *Lichtheimia corymbifera* | *Apiospora montagnei* |
| *Sporothrix schenckii* | *Exophiala jeanselmei* | *Sporothrix schenckii* | *Sporobolomyces roseus* |
| **13** | **14** | **15** | **16** |
| *Sarocladium strictum* | *Cladosporium herbarum* | *Cladosporium herbarum* | *Cladosporium cladosporioides* |
| *Aspergillus ochraceus* | *Fusarium culmorum* | *Purpureocillium lilacinum* | *Fusarium culmorum* |
| *Penicillium brevicompactum* | *Penicillium digitatum* | *Penicillium digitatum* | *Penicillium glabrum* |
| *Memnoniella echinata* | *Cephalotrichum stemonitis* | *Aureobasidium pullulans* | *Stachybotrys chartarum* |
| *Cryptococcus neoformans* | *Rhodotorula mucilaginosa* | *Rhodotorula mucilaginosa* | *Geotrichum candidum* |
| **17** | **18** | **19** | **20** |
| *Cladosporium cladosporioides* | *Penicillium citreonigrum* | *Gliomastix murorum* | *Cladosporium cladosporioides* |
| *Byssochlamys spectabilis* | *Byssochlamys spectabilis* | *Fusarium culmorum* | *Pupureocillium lilacinum* |
| *Penicillium glabrum* | *Penicillium corylophilum* | *Penicillium corylophilum* | *Penicillium chrysogenum* |
| *Epicoccum nigrum* | *Epicoccum nigrum* | *Penicillium chrysogenum* | *Phoma herbarum* |
| *Rhizopus arrhizus* | *Cryptococcus neoformans* | *Lichtheimia corymbifera* | *Saccharomyces cerevisiae* |
| **21** | **22** | **23** | **24** |
| *Alternaria alternata* | *Cryptococcus neoformans* | *Rhodotorula mucilaginosa* | *Cladosporium sphaerospermum* |
| *Aspergillus fumigatus* | *Aspergillus versicolor* | *Aspergillus fumigatus* | *Sarocladium strictum* |
| *Penicillium halotolerans* | *Penicillium digitatum* | *Penicillium digitatum* | *Penicillium brevicompactum* |
| *Trichothecium roseum* | *Scopulariopsis brevicaulis* | *Stachybotrys chartarum* | *Stachybotrys chartarum* |
| *Tritirachium oryzae* | *Rhizopus arrhizus* | *Scopulariopsis brevicaulis* | *Lichtheimia corymbifera* |
| **25** | **26** | **27** | **28** |
| *Cladosporium sphaerospermum* | *Cladosporium sphaerospermum* | *Syncephalastrum racemosum* | *Torula herbarum* |
| *Cryptococcus neoformans* | *Aspergillus ustus* | *Aspergillus versicolor* | *Aspergillus versicolor* |
| *Penicillium brevicompactum* | *Penicillium brevicompactum* | *Penicillium chrysogenum* | *Penicillium halotolerans* |
| *Stachybotrys chartarum* | *Phoma herbarum* | *Tritirachium oryzae* | *Cunninghamella echinulata* |
| *Lichtheimia corymbifera* | *Cryptococcus neoformans* | *Apiospora montagnei* | *Trichothecium roseum* |

| **29** | **30** | **31** | **32** |
| --- | --- | --- | --- |
| *Rhodotorula mucilaginosa* | *Sporothrix schenckii* | *Tritirachium oryzae* | *Cladosporium herbarum* |
| *Aspergillus fumigatus* | *Tritirachium oryzae* | *Aspergillus fumigatus* | *Thamnidium elegans* |
| *Penicillium chrysogenum* | *Penicillium chrysogenum* | *Penicillium aurantiogriseum* | *Penicillium aurantiogriseum* |
| *Trichothecium roseum* | *Cunninghamella echinulata* | *Trichothecium roseum* | *Memnoniella echinata* |
| *Thamnidium elegans* | *Beauveria bassiana* | *Pupureocillium lilacinum* | *Sporobolomyces roseus* |
| **33** | **34** | **35** | **36 - 37 (technical replicates)** |
| *Cladosporium cladosporioides* | *Cladosporium cladosporioides* | *Cladosporium herbarum* | *Penicillium halotolerans* |
| *Aspergillus chevalieri* | *Penicillium citreonigrum* | *Fusarium culmorum* | *Aspergillus ustus* |
| *Penicillium aurantiogriseum* | *Penicillium chrysogenum* | *Penicillium aurantiogriseum* | *Cladosporium cladosporioides* |
| *Alternaria alternata* | *Sarocladium strictum* | *Sarocladium strictum* | *Stachybotrys chartarum* |
| *Geotrichum candidum* | *Cunninghamella echinulata* | *Alternaria alternata* | *Saccharomyces cerevisiae* |

Table 2: Length (in base pairs) of the amplicons of each strain for region ITS1 and region ITS2, assessed by Tapestation D1000 analysis.

| **Species** | **Size ITS1 amplicon [bp]** | **Size ITS2 amplicon [bp]** |
| --- | --- | --- |
| *Alternaria alternata* | 232 | 289 |
| *Apiospora montagnei* | 255 | 296 |
| *Aspergillus chevalieri* | 216 | 299 |
| *Aspergillus floridensis* | 246 | 282 |
| *Aspergillus fumigatus* | 251 | 285 |
| *Aspergillus ochraceus* | 240 | 309 |
| *Aspergillus restrictus* | 258 | 304 |
| *Aspergillus subalbidus* | 245 | 299 |
| *Aspergillus ustus* | 225 | 304 |
| *Aspergillus versicolor* | 213 | 298 |
| *Aureobasidium pullulans* | 250 | 292 |
| *Beauveria bassiana* | 241 | 286 |
| *Cephalotrichum stemonitis* | 240 | 326 |
| *Chaetomium globosum* | 251 | 290 |
| *Cladosporium cladosporioides* | 218 | 277 |
| *Cladosporium herbarum* species complex | 215 | 279 |
| *Cladosporium sphaerospermum* | 230 | 284 |
| *Cryptococcus neoformans* | 193 | 326 |
| *Cunninghamella echinulata* | 344 | 565 |
| *Epicoccum nigrum* | 211 | 291 |
| *Exophiala jeanselmei* | 282 | 325 |
| *Fusarium culmorum* | 221 | 284 |
| *Geotrichum candidum* | / | 199 |
| *Gliomastix murorum* | 248 | 314 |
| *Lichtheimia corymbifera* | 349 | 380 |
| *Memnoniella echinata* | 232 | 311 |
| *Paecilomyces variotii* | 249 | 310 |
| *Penicillium aurantiogriseum* | 247 | 298 |
| *Penicillium brevicompactum* | 245 | 296 |
| *Penicillium chrysogenum* | 252 | 292 |
| *Penicillium citreonigrum* | 250 | 290 |
| *Penicillium corylophilum* | 250 | 296 |
| *Penicillium digitatum* | 246 | 303 |
| *Penicillium glabrum* | 249 | 283 |
| *Penicillium halotolerans* | 242 | 295 |
| *Phoma herbarum* | 212 | 294 |
| *Purpureocillium lilacinum* | 255 | 287 |
| *Rhizopus arrhizus* | 269 | 323 |
| *Rhodotorula mucilaginosa* | 224 | 345 |
| *Saccharomyces cerevisiae* | 426 | 374 |
| *Sarocladium strictum* | 253 | 310 |
| *Scedosporium apiospermum* | 241 | 343 |
| *Scopulariopsis brevicaulis* | 257 | 327 |
| *Sporobolomyces roseus* | 223 | 330 |
| *Sporothrix schenckii* | 261 | 332 |
| *Stachybotrys chartarum* | 245 | 301 |
| *Syncephalastrum racemosum* | 306 | 338 |
| *Thamnidium elegans* | 333 | 365 |
| *Torula herbarum* | 238 | 290 |
| *Trichothecium roseum* | 243 | 331 |
| *Tritirachium oryzae* | 349 | 357 |

Table 3: List of species with different names in the IHEM and UNITE ITS database and the synonyms they are known as in the other database. * indicates that the species is not found in the database but the genus is.

| **Name in DMC** | **Name in IHEM** | **Name in UNITE ITS** |
| --- | --- | --- |
| *Apiospora montagnei* | *Arthrinium arundinis* | *Arthrinium** |
| *Geotrichum candidum* | *Geotrichum candidum* | *Dipodascus geotrichum* |
| *Memnoniella echinata* | *Memnoniella echinata* | *Stachybotrys echinatus* |
| *Scedosporium apiospermum* | *Scedosporium apiospermum* | *Pseudoallescheria boydii* |
| *Scopulariopsis brevicaulis* | *Scopulariopsis brevicaulis* | *Microascus brevicaulis* |
| *Aspergillus chevalieri* | *Aspergillus chevalieri* | – |
| *Aspergillus floridensis* | *Aspergillus floridensis* | – |
| *Torula herbarum* | – | *Torula herbarum* |

Table 4: Overview of correctly identified species using Sanger sequences and the ITS1 region for classification. Each cell indicates whether the species was correctly identified at the species level (S), the genus level (G) or not identified (0) at any of these levels for that combination of database, software and method.

|  | **IHEM** | | | | **UNITE** | | | |
| --- | --- | --- | --- | --- | --- | --- | --- | --- |
|  | **BLAST** | | **Mothur** | | **BLAST** | | **Mothur** | |
| **Species** | **Strict** | **Loose** | **Wang** | **Knn** | **Strict** | **Loose** | **Wang** | **Knn** |
| *Alternaria alternata* | G | S | S | S | 0 | S | G | 0 |
| *Apiospora montagnei* | S | S | S | S | 0 | 0 | G | 0 |
| *Aspergillus chevalieri* | G | S | G | G | G | G | G | 0 |
| *Aspergillus floridensis* | G | G | G | G | G | G | G | G |
| *Aspergillus fumigatus* | S | S | S | S | S | S | G | S |
| *Aspergillus ochraceus* | S | S | G | S | G | S | S | G |
| *Aspergillus restrictus* | S | S | S | S | S | S | G | S |
| *Aspergillus subalbidus* | S | S | S | S | G | S | G | G |
| *Aspergillus ustus* | S | S | G | S | S | S | G | S |
| *Aspergillus versicolor* | G | S | G | S | G | G | G | 0 |
| *Aureobasidium pullulans* | G | S | G | G | S | S | S | S |
| *Beauveria bassiana* | S | S | S | S | S | S | S | S |
| *Cephalotrichum stemonitis* | G | S | G | G | S | S | G | G |
| *Chaetomium globosum* | S | S | S | S | G | S | G | G |
| *Cladosporium cladosporioides* | G | S | S | G | 0 | G | G | 0 |
| *Cladosporium herbarum* | G | S | G | G | 0 | 0 | 0 | 0 |
| *Cladosporium sphaerospermum* | S | S | S | S | S | S | S | S |
| *Cryptococcus neoformans* | S | S | S | S | S | S | S | S |
| *Cunninghamella echinulata* | 0 | 0 | S | S | S | S | S | G |
| *Epicoccum nigrum* | S | S | S | S | 0 | S | 0 | 0 |
| *Exophiala jeanselmei* | S | S | S | S | 0 | 0 | G | 0 |
| *Fusarium culmorum* | G | S | S | G | 0 | G | 0 | G |
| *Geotrichum candidum* | S | S | S | S | 0 | 0 | 0 | G |
| *Gliomastix murorum* | S | S | S | S | G | G | S | G |
| *Lichtheimia corymbifera* | S | S | S | S | S | S | S | S |
| *Memnoniella echinata* | S | S | S | S | S | S | S | S |
| *Paecilomyces variotii* | S | S | S | S | 0 | S | S | S |
| *Penicillium aurantiogriseum* | G | S | G | S | S | S | S | S |
| *Penicillium brevicompactum* | S | S | S | S | S | S | G | S |
| *Penicillium chrysogenum* | G | S | G | S | G | S | G | G |
| *Penicillium citreonigrum* | G | S | S | S | G | S | G | S |
| *Penicillium corylophilum* | G | S | S | S | G | S | S | S |
| *Penicillium digitatum* | S | S | S | S | 0 | 0 | G | 0 |
| *Penicillium glabrum* | G | S | S | S | G | G | G | G |
| *Penicillium halotolerans* | S | S | G | S | S | S | G | 0 |
| *Phoma herbarum* | S | S | 0 | S | S | S | 0 | S |
| *Purpureocillium lilacinum* | G | S | S | S | S | S | S | S |
| *Rhizopus arrhizus* | S | S | G | G | S | S | 0 | S |
| *Rhodotorula mucilaginosa* | S | S | S | S | S | S | S | S |
| *Saccharomyces cerevisiae* | S | S | S | S | S | S | S | S |
| *Sarocladium strictum* | S | S | S | S | G | G | G | G |
| *Scedosporium apiospermum* | S | S | S | S | 0 | 0 | 0 | 0 |
| *Scopulariopsis brevicaulis* | S | S | S | S | S | S | S | S |
| *Sporobolomyces roseus* | S | S | S | S | S | S | S | S |
| *Sporothrix schenckii* | S | S | S | S | G | G | G | S |
| *Stachybotrys chartarum* | G | S | S | S | 0 | 0 | S | 0 |
| *Syncephalastrum racemosum* | 0 | 0 | 0 | 0 | 0 | 0 | 0 | 0 |
| *Thamnidium elegans* | S | S | S | S | S | S | S | S |
| *Torula herbarum* | 0 | 0 | 0 | 0 | G | G | G | G |
| *Trichothecium roseum* | S | S | S | S | S | S | S | S |
| *Tritirachium oryzae* | S | S | S | S | S | S | S | S |

Table 5: Overview of incorrect identifications at the species and genus level using Sanger sequences and the ITS1 region for classification. Each cell indicates whether the species was identified (1) or not (0) for that combination of database, software and method.

|  | **IHEM** | | | | **UNITE** | | | |
| --- | --- | --- | --- | --- | --- | --- | --- | --- |
|  | **BLAST** | | **Mothur** | | **BLAST** | | **Mothur** | |
| **Species** | **Strict** | **Loose** | **Wang** | **Knn** | **Strict** | **Loose** | **Wang** | **Knn** |
| *Alternaria tenuissima* | 0 | 0 | 0 | 0 | 0 | 0 | 1 | 0 |
| *Arthrinium malaysianum* | 0 | 0 | 0 | 0 | 0 | 0 | 1 | 0 |
| *Aspergillus aculeatus* | 0 | 0 | 0 | 1 | 0 | 0 | 1 | 0 |
| *Aspergillus brunneoviolaceus* | 0 | 0 | 1 | 0 | 0 | 0 | 0 | 0 |
| *Aspergillus candidus* | 0 | 0 | 0 | 0 | 0 | 0 | 1 | 1 |
| *Aspergillus intermedius* | 0 | 0 | 0 | 0 | 0 | 0 | 1 | 0 |
| *Aspergillus japonicus* | 0 | 0 | 0 | 0 | 0 | 0 | 0 | 1 |
| *Aspergillus montevidensis* | 0 | 0 | 1 | 1 | 0 | 0 | 0 | 0 |
| *Aspergillus protuberus* | 0 | 0 | 0 | 0 | 1 | 1 | 1 | 0 |
| *Aureobasidium melanogenum* | 0 | 0 | 1 | 1 | 0 | 0 | 0 | 0 |
| *Cephalotrichum nanum* | 0 | 0 | 1 | 1 | 0 | 0 | 0 | 0 |
| *Cladosporium cucumerinum* | 0 | 0 | 0 | 1 | 0 | 0 | 0 | 0 |
| *Cladosporium delicatulum* | 0 | 0 | 0 | 0 | 0 | 0 | 1 | 0 |
| *Cladosporium sinuosum* | 0 | 0 | 0 | 1 | 0 | 0 | 0 | 0 |
| *Cunninghamella septata* | 0 | 0 | 0 | 0 | 0 | 0 | 0 | 1 |
| *Cyberlindnera jadinii* | 0 | 0 | 0 | 1 | 0 | 0 | 0 | 0 |
| *Fusarium sambucinum*species complex | 0 | 0 | 0 | 1 | 0 | 0 | 0 | 0 |
| *Gibberella zeae* | 0 | 0 | 0 | 0 | 1 | 1 | 0 | 0 |
| *Gliomastix tumulicola* | 0 | 0 | 0 | 0 | 1 | 1 | 0 | 1 |
| *Mycosphaerella tassiana* | 0 | 0 | 0 | 0 | 1 | 1 | 1 | 1 |
| *Penicillium nalgiovense* | 0 | 0 | 1 | 0 | 0 | 0 | 0 | 0 |
| *Penicillium paczoskii* | 0 | 0 | 0 | 0 | 1 | 1 | 0 | 1 |
| *Pseudallescheria boydii* | 0 | 0 | 0 | 0 | 0 | 0 | 1 | 1 |
| *Rhizopus oryzae* | 0 | 0 | 1 | 1 | 0 | 0 | 0 | 0 |
| *Sarocladium dejongiae* | 0 | 0 | 0 | 0 | 1 | 1 | 1 | 1 |
| *Sporothrix globosa* | 0 | 0 | 0 | 0 | 1 | 1 | 0 | 0 |
| **Genus** |  |  |  |  |  |  |  |  |
| *Aureobasidium* | 0 | 0 | 0 | 0 | 0 | 0 | 0 | 1 |
| *Cyberlindnera* | 0 | 0 | 0 | 1 | 0 | 0 | 0 | 0 |
| *Mrakia* | 0 | 0 | 0 | 0 | 0 | 0 | 1 | 1 |
| *Mycosphaerella* | 0 | 0 | 0 | 0 | 1 | 1 | 1 | 1 |
| *Pseudallescheria* | 0 | 0 | 0 | 0 | 0 | 0 | 1 | 1 |

Table 6: Overview of correctly identified species using Sanger sequences and the ITS2 region for classification. Each cell indicates whether the species was correctly identified at the species level (S), the genus level (G) or not identified (0) at any of these levels for that combination of database, software and method.

|  | **IHEM** | | | | **UNITE** | | | |
| --- | --- | --- | --- | --- | --- | --- | --- | --- |
|  | **BLAST** | | **Mothur** | | **BLAST** | | **Mothur** | |
| **Species** | **Strict** | **Loose** | **Wang** | **Knn** | **Strict** | **Loose** | **Wang** | **Knn** |
| *Alternaria alternata* | S | S | S | S | G | S | S | 0 |
| *Apiospora montagnei* | S | S | S | S | 0 | 0 | G | 0 |
| *Aspergillus chevalieri* | G | S | G | G | G | G | G | G |
| *Aspergillus floridensis* | G | S | G | G | G | G | G | G |
| *Aspergillus fumigatus* | 0 | S | S | S | S | S | G | S |
| *Aspergillus ochraceus* | G | S | G | G | G | S | S | S |
| *Aspergillus restrictus* | S | S | S | S | S | S | S | S |
| *Aspergillus subalbidus* | G | S | S | S | G | S | G | G |
| *Aspergillus ustus* | G | S | G | G | G | S | G | S |
| *Aspergillus versicolor* | G | S | G | S | G | G | G | 0 |
| *Aureobasidium pullulans* | S | S | S | S | S | S | S | S |
| *Beauveria bassiana* | S | S | S | S | 0 | S | S | 0 |
| *Cephalotrichum stemonitis* | S | S | G | S | S | S | S | S |
| *Chaetomium globosum* | S | S | S | S | G | S | G | S |
| *Cladosporium cladosporioides* | G | S | S | G | G | S | G | 0 |
| *Cladosporium herbarum* | G | S | G | G | 0 | 0 | 0 | 0 |
| *Cladosporium sphaerospermum* | S | S | S | S | S | S | S | S |
| *Cryptococcus neoformans* | G | S | S | S | S | S | S | S |
| *Cunninghamella echinulata* | S | S | S | S | S | S | S | S |
| *Epicoccum nigrum* | S | S | S | S | 0 | S | 0 | S |
| *Exophiala jeanselmei* | S | S | S | S | 0 | 0 | G | 0 |
| *Fusarium culmorum* | G | S | S | G | 0 | G | G | 0 |
| *Geotrichum candidum* | S | S | S | S | S | S | S | 0 |
| *Gliomastix murorum* | S | S | S | S | 0 | 0 | 0 | 0 |
| *Lichtheimia corymbifera* | S | S | S | S | S | S | S | S |
| *Memnoniella echinata* | S | S | S | S | 0 | 0 | 0 | 0 |
| *Paecilomyces variotii* | S | S | S | S | 0 | S | S | S |
| *Penicillium aurantiogriseum* | G | S | G | S | S | S | S | S |
| *Penicillium brevicompactum* | S | S | S | S | G | S | S | G |
| *Penicillium chrysogenum* | G | S | G | S | G | S | G | 0 |
| *Penicillium citreonigrum* | S | S | S | S | S | S | S | S |
| *Penicillium corylophilum* | S | S | S | S | S | S | S | 0 |
| *Penicillium digitatum* | S | S | S | S | 0 | 0 | G | 0 |
| *Penicillium glabrum* | G | S | S | S | G | G | G | 0 |
| *Penicillium halotolerans* | G | S | G | G | G | S | G | 0 |
| *Phoma herbarum* | S | S | 0 | S | S | S | 0 | S |
| *Purpureocillium lilacinum* | G | S | S | S | 0 | 0 | G | 0 |
| *Rhizopus arrhizus* | S | S | G | G | S | S | S | S |
| *Rhodotorula mucilaginosa* | S | S | S | S | S | S | S | S |
| *Saccharomyces cerevisiae* | S | S | S | S | G | G | G | G |
| *Sarocladium strictum* | S | S | S | S | G | G | G | G |
| *Scedosporium apiospermum* | S | S | S | S | 0 | 0 | 0 | 0 |
| *Scopulariopsis brevicaulis* | S | S | S | S | S | S | 0 | S |
| *Sporobolomyces roseus* | S | S | S | S | S | S | S | S |
| *Sporothrix schenckii* | S | S | S | S | S | S | S | S |
| *Stachybotrys chartarum* | S | S | S | S | S | S | S | S |
| *Syncephalastrum racemosum* | 0 | 0 | 0 | 0 | 0 | 0 | 0 | 0 |
| *Thamnidium elegans* | 0 | 0 | 0 | 0 | 0 | 0 | 0 | 0 |
| *Torula herbarum* | 0 | 0 | 0 | 0 | G | G | G | G |
| *Trichothecium roseum* | S | S | S | S | S | S | S | S |
| *Tritirachium oryzae* | S | S | S | S | S | S | S | S |

Table 7: Overview of incorrect identifications at the species and genus level using Sanger sequences and the ITS2 region for classification. Each cell indicates whether the species was identified (1) or not (0) for that combination of database, software and method.

|  | **IHEM** | | | | **UNITE** | | | |
| --- | --- | --- | --- | --- | --- | --- | --- | --- |
|  | **BLAST** | | **Mothur** | | **BLAST** | | **Mothur** | |
| **Species** | **Strict** | **Loose** | **Wang** | **Knn** | **Strict** | **Loose** | **Wang** | **Knn** |
| *Arthrinium malaysianum* | 0 | 0 | 0 | 0 | 0 | 0 | 1 | 0 |
| *Aspergillus aculeatus* | 0 | 0 | 0 | 1 | 0 | 0 | 0 | 0 |
| *Aspergillus brunneoviolaceus* | 0 | 0 | 1 | 0 | 0 | 0 | 0 | 1 |
| *Aspergillus candidus* | 0 | 0 | 0 | 0 | 0 | 0 | 0 | 1 |
| *Aspergillus melleus* | 0 | 0 | 0 | 1 | 0 | 0 | 0 | 0 |
| *Aspergillus montevidensis* | 0 | 0 | 0 | 1 | 0 | 0 | 0 | 1 |
| *Aspergillus puniceus* | 0 | 0 | 1 | 1 | 0 | 0 | 0 | 0 |
| *Aspergillus puulaauensis* | 0 | 0 | 0 | 0 | 1 | 1 | 0 | 0 |
| *Cladosporium cucumerinum* | 0 | 0 | 0 | 1 | 0 | 0 | 0 | 0 |
| *Cladosporium ramotenellum* | 0 | 0 | 0 | 1 | 0 | 0 | 0 | 0 |
| *Cladosporium tenuissimum* | 0 | 0 | 0 | 0 | 0 | 0 | 1 | 0 |
| *Cordyceps bassiana* | 0 | 0 | 0 | 0 | 0 | 0 | 0 | 1 |
| *Fusarium sambucinum*species complex | 0 | 0 | 0 | 1 | 0 | 0 | 0 | 0 |
| *Gibberella zeae* | 0 | 0 | 0 | 0 | 0 | 0 | 0 | 1 |
| *Mycosphaerella tassiana* | 0 | 0 | 0 | 0 | 0 | 1 | 0 | 0 |
| *Penicillium chrysogenum* | 0 | 0 | 0 | 1 | 0 | 0 | 0 | 0 |
| *Pithomyces chartarum* | 0 | 0 | 0 | 1 | 0 | 0 | 0 | 0 |
| *Pseudallescheria boydii* | 0 | 0 | 0 | 0 | 1 | 1 | 0 | 1 |
| *Rhizopus oryzae* | 0 | 0 | 1 | 1 | 0 | 0 | 0 | 0 |
| *Sarocladium dejongiae* | 0 | 0 | 0 | 0 | 1 | 1 | 1 | 1 |
| **Genus** |  |  |  |  |  |  |  |  |
| *Cordyceps* | 0 | 0 | 0 | 0 | 0 | 0 | 0 | 1 |
| *Gibberella* | 0 | 0 | 0 | 0 | 0 | 0 | 0 | 1 |
| *Pithomyces* | 0 | 0 | 0 | 1 | 0 | 0 | 0 | 0 |
| *Pseudallescheria* | 0 | 0 | 0 | 0 | 1 | 1 | 0 | 1 |

Table 8: Overview of correctly identified species using Sanger sequences and the consensus approach for classification. Each cell indicates whether the species was correctly identified at the species level (S), the genus level (G) or not identified (0) at any of these levels for that combination of database, software and method.

|  | **IHEM** | | | | **UNITE** | | | |
| --- | --- | --- | --- | --- | --- | --- | --- | --- |
|  | **BLAST** | | **Mothur** | | **BLAST** | | **Mothur** | |
| **Species** | **Strict** | **Loose** | **Wang** | **Knn** | **Strict** | **Loose** | **Wang** | **Knn** |
| *Alternaria alternata* | G | S | S | S | 0 | S | G | 0 |
| *Apiospora montagnei* | S | S | S | S | 0 | 0 | G | 0 |
| *Aspergillus chevalieri* | G | S | G | G | G | G | G | 0 |
| *Aspergillus floridensis* | G | G | G | G | G | G | G | G |
| *Aspergillus fumigatus* | 0 | S | S | S | S | S | G | S |
| *Aspergillus ochraceus* | G | S | G | G | G | S | S | G |
| *Aspergillus restrictus* | S | S | S | S | S | S | G | S |
| *Aspergillus subalbidus* | G | S | S | S | G | S | G | G |
| *Aspergillus ustus* | G | S | G | G | G | S | G | S |
| *Aspergillus versicolor* | G | S | G | S | G | G | G | 0 |
| *Aureobasidium pullulans* | G | S | G | G | S | S | S | S |
| *Beauveria bassiana* | S | S | S | S | 0 | S | S | 0 |
| *Cephalotrichum stemonitis* | G | S | G | G | S | S | G | G |
| *Chaetomium globosum* | S | S | S | S | G | S | G | G |
| *Cladosporium cladosporioides* | G | S | S | G | 0 | G | G | 0 |
| *Cladosporium herbarum* | G | S | G | G | 0 | 0 | 0 | 0 |
| *Cladosporium sphaerospermum* | S | S | S | S | S | S | S | S |
| *Cryptococcus neoformans* | G | S | S | S | S | S | S | S |
| *Cunninghamella echinulata* | 0 | 0 | S | S | S | S | S | G |
| *Epicoccum nigrum* | S | S | S | S | 0 | S | 0 | 0 |
| *Exophiala jeanselmei* | S | S | S | S | 0 | 0 | G | 0 |
| *Fusarium culmorum* | G | S | S | G | 0 | G | 0 | 0 |
| *Geotrichum candidum* | S | S | S | S | 0 | 0 | 0 | 0 |
| *Gliomastix murorum* | S | S | S | S | 0 | 0 | 0 | 0 |
| *Lichtheimia corymbifera* | S | S | S | S | S | S | S | S |
| *Memnoniella echinata* | S | S | S | S | 0 | 0 | 0 | 0 |
| *Paecilomyces variotii* | S | S | S | S | 0 | S | S | S |
| *Penicillium aurantiogriseum* | G | S | G | S | S | S | S | S |
| *Penicillium brevicompactum* | S | S | S | S | G | S | G | G |
| *Penicillium chrysogenum* | G | S | G | S | G | S | G | 0 |
| *Penicillium citreonigrum* | G | S | S | S | G | S | G | S |
| *Penicillium corylophilum* | G | S | S | S | G | S | S | 0 |
| *Penicillium digitatum* | S | S | S | S | 0 | 0 | G | 0 |
| *Penicillium glabrum* | G | S | S | S | G | G | G | 0 |
| *Penicillium halotolerans* | G | S | G | G | G | S | G | 0 |
| *Phoma herbarum* | S | S | 0 | S | S | S | 0 | S |
| *Purpureocillium lilacinum* | G | S | S | S | 0 | 0 | G | 0 |
| *Rhizopus arrhizus* | S | S | G | G | S | S | 0 | S |
| *Rhodotorula mucilaginosa* | S | S | S | S | S | S | S | S |
| *Saccharomyces cerevisiae* | S | S | S | S | G | G | G | G |
| *Sarocladium strictum* | S | S | S | S | G | G | G | G |
| *Scedosporium apiospermum* | S | S | S | S | 0 | 0 | 0 | 0 |
| *Scopulariopsis brevicaulis* | S | S | S | S | S | S | 0 | S |
| *Sporobolomyces roseus* | S | S | S | S | S | S | S | S |
| *Sporothrix schenckii* | S | S | S | S | G | G | G | S |
| *Stachybotrys chartarum* | G | S | S | S | 0 | 0 | S | 0 |
| *Syncephalastrum racemosum* | 0 | 0 | 0 | 0 | 0 | 0 | 0 | 0 |
| *Thamnidium elegans* | 0 | 0 | 0 | 0 | 0 | 0 | 0 | 0 |
| *Torula herbarum* | 0 | 0 | 0 | 0 | G | G | G | G |
| *Trichothecium roseum* | S | S | S | S | S | S | S | S |
| *Tritirachium oryzae* | S | S | S | S | S | S | S | S |

Table 9: Overview of incorrect identifications at the species and genus level using Sanger sequences and the consensus approach for classification. Each cell indicates whether the species was identified (1) or not (0) for that combination of database, software and method.

|  | **IHEM** | | | | **UNITE** | | | |
| --- | --- | --- | --- | --- | --- | --- | --- | --- |
|  | **BLAST** | | **Mothur** | | **BLAST** | | **Mothur** | |
| **Species** | **Strict** | **Loose** | **Wang** | **Knn** | **Strict** | **Loose** | **Wang** | **Knn** |
| *Arthrinium malaysianum* | 0 | 0 | 0 | 0 | 0 | 0 | 1 | 0 |
| *Aspergillus aculeatus* | 0 | 0 | 0 | 1 | 0 | 0 | 0 | 0 |
| *Aspergillus brunneoviolaceus* | 0 | 0 | 1 | 0 | 0 | 0 | 0 | 0 |
| *Aspergillus candidus* | 0 | 0 | 0 | 0 | 0 | 0 | 0 | 1 |
| *Aspergillus montevidensis* | 0 | 0 | 0 | 1 | 0 | 0 | 0 | 0 |
| *Cladosporium cucumerinum* | 0 | 0 | 0 | 1 | 0 | 0 | 0 | 0 |
| *Fusarium sambucinum*species complex | 0 | 0 | 0 | 1 | 0 | 0 | 0 | 0 |
| *Mycosphaerella tassiana* | 0 | 0 | 0 | 0 | 0 | 1 | 0 | 0 |
| *Pseudallescheria boydii* | 0 | 0 | 0 | 0 | 0 | 0 | 0 | 1 |
| *Rhizopus oryzae* | 0 | 0 | 1 | 1 | 0 | 0 | 0 | 0 |
| *Sarocladium dejongiae* | 0 | 0 | 0 | 0 | 1 | 1 | 1 | 1 |
| **Genus** |  |  |  |  |  |  |  |  |
| *Pseudallescheria* | 0 | 0 | 0 | 0 | 0 | 0 | 0 | 1 |

Table 10: Overview of correctly identified species using Illumina sequences and the ITS1 region for classification for each DMC. Each cell indicates whether the species was correctly identified at the species level (S), the genus level (G) or not identified (0) at any of these levels for that combination of database, software and method.

|  | **IHEM** | | | | **UNITE** | | | |
| --- | --- | --- | --- | --- | --- | --- | --- | --- |
|  | **BLAST** | | **MOTHUR** | | **BLAST** | | **MOTHUR** | |
| **Species - DMC** | **Strict** | **Loose** | **Wang** | **knn** | **Strict** | **Loose** | **Wang** | **knn** |
|  |  |  |  |  |  |  |  |  |
| **DMC 1** |  |  |  |  |  |  |  |  |
| *Aspergillus fumigatus* | S | S | S | S | G | G | G | G |
| *Aspergillus versicolor* | G | S | G | G | G | G | G | G |
| *Cladosporium sphaerospermum* | S | S | S | S | S | S | S | S |
| *Penicillium chrysogenum* | G | S | G | G | S | S | G | G |
| *Stachybotrys chartarum* | G | S | S | S | 0 | 0 | S | S |
|  |  |  |  |  |  |  |  |  |
| **DMC 2** |  |  |  |  |  |  |  |  |
| *Aspergillus versicolor* | G | S | G | G | 0 | 0 | G | G |
| *Aureobasidium pullulans* | G | S | G | G | G | G | 0 | 0 |
| *Cladosporium herbarum* | G | S | S | S | G | G | G | G |
| *Penicillium aurantiogriseum* | G | S | G | G | S | S | S | S |
| *Stachybotrys chartarum* | G | S | S | S | 0 | 0 | S | S |
|  |  |  |  |  |  |  |  |  |
| **DMC 3** |  |  |  |  |  |  |  |  |
| *Aspergillus ochraceus* | S | S | G | G | S | S | G | G |
| *Chaetomium globosum* | S | S | S | S | G | S | G | G |
| *Cladosporium cladosporioides* | G | S | G | G | G | G | G | G |
| *Rhizopus arrhizus* | S | S | G | G | S | S | S | S |
| *Saccharomyces cerevisiae* | S | S | S | S | S | S | S | S |
|  |  |  |  |  |  |  |  |  |
| **DMC 4** |  |  |  |  |  |  |  |  |
| *Aspergillus fumigatus* | S | S | S | S | G | G | G | G |
| *Chaetomium globosum* | S | S | S | S | G | S | G | G |
| *Cladosporium sphaerospermum* | S | S | S | S | S | S | S | S |
| *Memnoniella echinata* | S | S | S | S | S | S | S | S |
| *Penicillium chrysogenum* | G | S | G | G | S | S | G | G |
|  |  |  |  |  |  |  |  |  |
| **DMC 5** |  |  |  |  |  |  |  |  |
| *Aspergillus chevalieri* | G | S | G | G | G | G | G | G |
| *Cephalotrichum stemonitis* | G | S | G | G | G | G | G | G |
| *Cladosporium cladosporioides* | G | S | G | G | G | G | G | G |
| *Scedosporium apiospermum* | S | S | S | S | 0 | 0 | 0 | 0 |
| *Syncephalastrum racemosum* | 0 | 0 | G | G | S | S | G | G |
|  |  |  |  |  |  |  |  |  |
| **DMC 6** |  |  |  |  |  |  |  |  |
| *Aspergillus subalbidus* | S | S | S | S | G | G | G | G |
| *Exophiala jeanselmei* | S | S | S | S | 0 | 0 | G | G |
| *Saccharomyces cerevisiae* | S | S | S | S | S | S | S | S |
| *Sporobolomyces roseus* | S | S | S | S | S | S | S | S |
| *Torula herbarum* | 0 | 0 | 0 | 0 | 0 | 0 | G | G |
|  |  |  |  |  |  |  |  |  |
| **DMC 7** |  |  |  |  |  |  |  |  |
| *Apiospora montagnei* | S | S | S | S | 0 | 0 | G | G |
| *Cladosporium herbarum* | G | S | S | S | 0 | 0 | 0 | 0 |
| *Exophiala jeanselmei* | S | S | S | S | 0 | 0 | G | G |
| *Paecilomyces variotii* | S | S | S | S | S | S | S | S |
| *Scopulariopsis brevicaulis* | S | S | S | S | S | S | S | S |
|  |  |  |  |  |  |  |  |  |
| **DMC 8** |  |  |  |  |  |  |  |  |
| *Aspergillus floridensis* | G | G | G | G | G | G | G | G |
| *Beauveria bassiana* | S | S | S | S | S | S | 0 | 0 |
| *Cephalotrichum stemonitis* | G | S | G | G | G | G | G | G |
| *Cladosporium sphaerospermum* | S | S | S | S | S | S | S | S |
| *Syncephalastrum racemosum* | 0 | 0 | G | G | S | S | G | G |
|  |  |  |  |  |  |  |  |  |
| **DMC 9** |  |  |  |  |  |  |  |  |
| *Aspergillus restrictus* | S | S | S | S | S | S | S | S |
| *Cladosporium cladosporioides* | G | S | G | G | G | G | G | G |
| *Exophiala jeanselmei* | S | S | S | S | 0 | 0 | G | G |
| *Rhizopus arrhizus* | S | S | G | G | S | S | S | S |
| *Sporothrix schenckii* | S | S | S | S | G | G | G | G |
|  |  |  |  |  |  |  |  |  |
| **DMC 10** |  |  |  |  |  |  |  |  |
| *Alternaria alternata* | G | S | G | G | G | G | G | G |
| *Cladosporium sphaerospermum* | S | S | S | S | S | S | S | S |
| *Exophiala jeanselmei* | S | S | S | S | 0 | 0 | G | G |
| *Purpureocillium lilacinum* | G | S | S | S | S | S | S | S |
| *Rhodotorula mucilaginosa* | S | S | S | S | S | S | G | G |
|  |  |  |  |  |  |  |  |  |
| **DMC 11** |  |  |  |  |  |  |  |  |
| *Aspergillus restrictus* | S | S | S | S | S | S | S | S |
| *Cephalotrichum stemonitis* | G | S | G | G | G | G | G | G |
| *Cladosporium sphaerospermum* | S | S | S | S | S | S | S | S |
| *Lichtheimia corymbifera* | S | S | S | S | S | S | S | S |
| *Sporothrix schenckii* | S | S | S | S | G | G | G | G |
|  |  |  |  |  |  |  |  |  |
| **DMC 12** |  |  |  |  |  |  |  |  |
| *Apiospora montagnei* | S | S | S | S | 0 | 0 | G | G |
| *Aspergillus ustus* | S | S | G | G | S | S | G | G |
| *Cladosporium herbarum* | G | S | S | S | 0 | 0 | 0 | 0 |
| *Penicillium brevicompactum* | S | S | S | S | S | S | G | G |
| *Sporobolomyces roseus* | S | S | S | S | S | S | S | S |
|  |  |  |  |  |  |  |  |  |
| **DMC 13** |  |  |  |  |  |  |  |  |
| *Aspergillus ochraceus* | S | S | G | G | S | S | G | G |
| *Cryptococcus neoformans* | S | S | S | S | S | S | S | S |
| *Memnoniella echinata* | S | S | S | S | S | S | S | S |
| *Penicillium brevicompactum* | S | S | S | S | S | S | G | G |
| *Sarocladium strictum* | S | S | S | S | G | G | G | G |
|  |  |  |  |  |  |  |  |  |
| **DMC 14** |  |  |  |  |  |  |  |  |
| *Cephalotrichum stemonitis* | G | S | G | G | G | G | G | G |
| *Cladosporium herbarum* | G | S | S | S | 0 | 0 | 0 | 0 |
| *Fusarium culmorum* | G | S | S | S | S | S | 0 | 0 |
| *Penicillium digitatum* | S | S | S | S | 0 | 0 | G | G |
| *Rhodotorula mucilaginosa* | S | S | S | S | S | S | G | G |
|  |  |  |  |  |  |  |  |  |
| **DMC 15** |  |  |  |  |  |  |  |  |
| *Aureobasidium pullulans* | G | S | G | G | S | S | 0 | 0 |
| *Cladosporium herbarum* | G | S | S | S | 0 | 0 | 0 | 0 |
| *Penicillium digitatum* | S | S | S | S | 0 | 0 | G | G |
| *Purpureocillium lilacinum* | G | S | S | S | S | S | S | S |
| *Rhodotorula mucilaginosa* | S | S | S | S | S | S | G | G |
|  |  |  |  |  |  |  |  |  |
| **DMC 16** |  |  |  |  |  |  |  |  |
| *Cladosporium cladosporioides* | G | S | G | G | G | G | G | G |
| *Fusarium culmorum* | G | S | S | S | G | G | 0 | 0 |
| *Geotrichum candidum* | S | S | S | S | 0 | 0 | 0 | 0 |
| *Penicillium glabrum* | G | S | S | S | G | G | G | G |
| *Stachybotrys chartarum* | G | S | S | S | 0 | 0 | S | S |
|  |  |  |  |  |  |  |  |  |
| **DMC 17** |  |  |  |  |  |  |  |  |
| *Cladosporium cladosporioides* | G | S | G | G | G | G | G | G |
| *Epicoccum nigrum* | S | S | S | S | G | S | 0 | 0 |
| *Paecilomyces variotii* | S | S | S | S | S | S | S | S |
| *Penicillium glabrum* | G | S | S | S | G | G | G | G |
| *Rhizopus arrhizus* | S | S | G | G | S | S | S | S |
|  |  |  |  |  |  |  |  |  |
| **DMC 18** |  |  |  |  |  |  |  |  |
| *Cryptococcus neoformans* | S | S | S | S | S | S | S | S |
| *Epicoccum nigrum* | S | S | S | S | G | S | 0 | 0 |
| *Paecilomyces variotii* | S | S | S | S | S | S | S | S |
| *Penicillium citreonigrum* | G | S | S | S | G | S | G | G |
| *Penicillium corylophilum* | G | S | S | S | G | S | S | S |
|  |  |  |  |  |  |  |  |  |
| **DMC 19** |  |  |  |  |  |  |  |  |
| *Fusarium culmorum* | G | S | S | S | S | S | 0 | 0 |
| *Gliomastix murorum* | S | S | S | S | S | S | S | S |
| *Lichtheimia corymbifera* | S | S | S | S | S | S | S | S |
| *Penicillium chrysogenum* | G | S | G | G | S | S | G | G |
| *Penicillium corylophilum* | G | S | S | S | G | S | S | S |
|  |  |  |  |  |  |  |  |  |
| **DMC 20** |  |  |  |  |  |  |  |  |
| *Cladosporium cladosporioides* | G | S | G | G | G | G | G | G |
| *Penicillium chrysogenum* | G | S | G | G | S | S | G | G |
| *Phoma herbarum* | S | S | 0 | 0 | S | S | 0 | 0 |
| *Purpureocillium lilacinum* | G | S | S | S | S | S | S | S |
| *Saccharomyces cerevisiae* | S | S | S | S | S | S | S | S |
|  |  |  |  |  |  |  |  |  |
| **DMC 21** |  |  |  |  |  |  |  |  |
| *Alternaria alternata* | G | S | G | G | G | G | G | G |
| *Aspergillus fumigatus* | S | S | S | S | G | G | G | G |
| *Penicillium halotolerans* | S | S | G | G | G | G | G | G |
| *Trichothecium roseum* | S | S | S | S | S | S | S | S |
| *Tritirachium oryzae* | S | S | S | S | S | S | S | S |
|  |  |  |  |  |  |  |  |  |
| **DMC 22** |  |  |  |  |  |  |  |  |
| *Aspergillus versicolor* | G | S | G | G | 0 | 0 | G | G |
| *Cryptococcus neoformans* | S | S | S | S | S | S | S | S |
| *Penicillium digitatum* | S | S | S | S | 0 | 0 | G | G |
| *Rhizopus arrhizus* | S | S | G | G | S | S | S | S |
| *Scopulariopsis brevicaulis* | S | S | S | S | S | S | S | S |
|  |  |  |  |  |  |  |  |  |
| **DMC 23** |  |  |  |  |  |  |  |  |
| *Aspergillus fumigatus* | S | S | S | S | G | G | G | G |
| *Penicillium digitatum* | S | S | S | S | 0 | 0 | G | G |
| *Rhodotorula mucilaginosa* | S | S | S | S | S | S | G | G |
| *Scopulariopsis brevicaulis* | S | S | S | S | S | S | S | S |
| *Stachybotrys chartarum* | G | S | S | S | 0 | 0 | S | S |
|  |  |  |  |  |  |  |  |  |
| **DMC 24** |  |  |  |  |  |  |  |  |
| *Cladosporium sphaerospermum* | S | S | S | S | S | S | S | S |
| *Lichtheimia corymbifera* | S | S | S | S | S | S | S | S |
| *Penicillium brevicompactum* | S | S | S | S | S | S | G | G |
| *Sarocladium strictum* | S | S | S | S | G | G | G | G |
| *Stachybotrys chartarum* | G | S | S | S | 0 | 0 | S | S |
|  |  |  |  |  |  |  |  |  |
| **DMC 25** |  |  |  |  |  |  |  |  |
| *Cladosporium sphaerospermum* | S | S | S | S | S | S | S | S |
| *Cryptococcus neoformans* | S | S | S | S | S | S | S | S |
| *Lichtheimia corymbifera* | S | S | S | S | S | S | S | S |
| *Penicillium brevicompactum* | S | S | S | S | S | S | G | G |
| *Stachybotrys chartarum* | G | S | S | S | 0 | 0 | S | S |
|  |  |  |  |  |  |  |  |  |
| **DMC 26** |  |  |  |  |  |  |  |  |
| *Aspergillus ustus* | S | S | G | G | S | S | G | G |
| *Cladosporium sphaerospermum* | S | S | S | S | S | S | S | S |
| *Cryptococcus neoformans* | S | S | S | S | S | S | S | S |
| *Penicillium brevicompactum* | S | S | S | S | S | S | G | G |
| *Phoma herbarum* | S | S | 0 | 0 | S | S | 0 | 0 |
|  |  |  |  |  |  |  |  |  |
| **DMC 27** |  |  |  |  |  |  |  |  |
| *Apiospora montagnei* | S | S | S | S | 0 | 0 | G | G |
| *Aspergillus versicolor* | G | S | G | G | 0 | 0 | G | G |
| *Penicillium chrysogenum* | G | S | G | G | S | S | G | G |
| *Syncephalastrum racemosum* | 0 | 0 | G | G | S | S | G | G |
| *Tritirachium oryzae* | S | S | S | S | S | S | S | S |
|  |  |  |  |  |  |  |  |  |
| **DMC 28** |  |  |  |  |  |  |  |  |
| *Aspergillus versicolor* | G | S | G | G | 0 | 0 | G | G |
| *Cunninghamella echinulata* | S | S | S | S | G | G | S | S |
| *Penicillium halotolerans* | S | S | G | G | G | G | G | G |
| *Torula herbarum* | 0 | 0 | 0 | 0 | G | G | G | G |
| *Trichothecium roseum* | S | S | S | S | S | S | S | S |
|  |  |  |  |  |  |  |  |  |
| **DMC 29** |  |  |  |  |  |  |  |  |
| *Aspergillus fumigatus* | S | S | S | S | G | G | G | G |
| *Penicillium chrysogenum* | G | S | G | G | S | S | G | G |
| *Rhodotorula mucilaginosa* | S | S | S | S | S | S | G | G |
| *Thamnidium elegans* | S | S | S | S | S | S | S | S |
| *Trichothecium roseum* | S | S | S | S | S | S | S | S |
|  |  |  |  |  |  |  |  |  |
| **DMC 30** |  |  |  |  |  |  |  |  |
| *Beauveria bassiana* | S | S | S | S | S | S | 0 | 0 |
| *Cunninghamella echinulata* | S | S | S | S | G | G | S | S |
| *Penicillium chrysogenum* | G | S | G | G | S | S | G | G |
| *Sporothrix schenckii* | S | S | S | S | G | G | G | G |
| *Tritirachium oryzae* | S | S | S | S | S | S | S | S |
|  |  |  |  |  |  |  |  |  |
| **DMC 31** |  |  |  |  |  |  |  |  |
| *Aspergillus fumigatus* | S | S | S | S | G | G | G | G |
| *Penicillium aurantiogriseum* | G | S | G | G | S | S | S | S |
| *Purpureocillium lilacinum* | G | S | S | S | S | S | S | S |
| *Trichothecium roseum* | S | S | S | S | S | S | S | S |
| *Tritirachium oryzae* | S | S | S | S | S | S | S | S |
|  |  |  |  |  |  |  |  |  |
| **DMC 32** |  |  |  |  |  |  |  |  |
| *Cladosporium herbarum* | G | S | S | S | G | G | G | G |
| *Memnoniella echinata* | S | S | S | S | S | S | S | S |
| *Penicillium aurantiogriseum* | G | S | G | G | S | S | S | S |
| *Sporobolomyces roseus* | S | S | S | S | S | S | S | S |
| *Thamnidium elegans* | S | S | S | S | S | S | S | S |
|  |  |  |  |  |  |  |  |  |
| **DMC 33** |  |  |  |  |  |  |  |  |
| *Alternaria alternata* | G | S | G | G | G | G | G | G |
| *Aspergillus chevalieri* | G | S | G | G | G | G | G | G |
| *Cladosporium cladosporioides* | G | S | G | G | G | G | G | G |
| *Geotrichum candidum* | S | S | S | S | 0 | 0 | 0 | 0 |
| *Penicillium aurantiogriseum* | G | S | G | G | S | S | S | S |
|  |  |  |  |  |  |  |  |  |
| **DMC 34** |  |  |  |  |  |  |  |  |
| *Cladosporium cladosporioides* | G | S | G | G | G | G | G | G |
| *Cunninghamella echinulata* | S | S | S | S | G | G | S | S |
| *Penicillium chrysogenum* | G | S | G | G | S | S | G | G |
| *Penicillium citreonigrum* | G | S | S | S | G | S | G | G |
| *Sarocladium strictum* | S | S | S | S | G | G | G | G |
|  |  |  |  |  |  |  |  |  |
| **DMC 35** |  |  |  |  |  |  |  |  |
| *Alternaria alternata* | G | S | G | G | G | G | G | G |
| *Cladosporium herbarum* | G | S | S | S | 0 | 0 | 0 | 0 |
| *Fusarium culmorum* | G | S | S | S | S | S | 0 | 0 |
| *Penicillium aurantiogriseum* | G | S | G | G | S | S | S | S |
| *Sarocladium strictum* | S | S | S | S | G | G | G | G |
|  |  |  |  |  |  |  |  |  |
| **DMC 36** |  |  |  |  |  |  |  |  |
| *Aspergillus ustus* | S | S | G | G | S | S | G | G |
| *Cladosporium cladosporioides* | G | S | G | G | G | G | G | G |
| *Penicillium halotolerans* | S | S | G | G | G | G | G | G |
| *Saccharomyces cerevisiae* | S | S | S | S | S | S | S | S |
| *Stachybotrys chartarum* | G | S | S | S | 0 | 0 | S | S |
|  |  |  |  |  |  |  |  |  |
| **DMC 37** |  |  |  |  |  |  |  |  |
| *Aspergillus ustus* | S | S | G | G | S | S | G | G |
| *Cladosporium cladosporioides* | G | S | G | G | G | G | G | G |
| *Penicillium halotolerans* | S | S | G | G | G | G | G | G |
| *Saccharomyces cerevisiae* | S | S | S | S | S | S | S | S |
| *Stachybotrys chartarum* | G | S | S | S | 0 | 0 | S | S |

Table 11: Overview of false positives identifies using Illumina sequences and the ITS1 region for classification for each DMC. Each cell indicates whether the species was identified (1) or not (0) for that combination of database, software and method.

|  | **IHEM** | | | | **UNITE** | | | |
| --- | --- | --- | --- | --- | --- | --- | --- | --- |
|  | **BLAST** | | **MOTHUR** | | **BLAST** | | **MOTHUR** | |
| **Species - DMC** | **Strict** | **Loose** | **Wang** | **knn** | **Strict** | **Loose** | **Wang** | **knn** |
|  |  |  |  |  |  |  |  |  |
| **DMC 1** |  |  |  |  |  |  |  |  |
| **Species** |  |  |  |  |  |  |  |  |
| *Aspergillus fischeri* | 0 | 0 | 0 | 0 | 1 | 1 | 0 | 0 |
| *Aspergillus protuberus* | 0 | 0 | 0 | 0 | 0 | 0 | 1 | 1 |
| *Cladosporium delicatulum* | 0 | 0 | 0 | 0 | 0 | 0 | 1 | 1 |
| *Cladosporium tenuissimum* | 0 | 1 | 0 | 0 | 0 | 0 | 0 | 0 |
| *Geotrichum candidum* | 1 | 1 | 1 | 1 | 0 | 0 | 0 | 0 |
|  |  |  |  |  |  |  |  |  |
| **Genus** |  |  |  |  |  |  |  |  |
| *Geotrichum* | 1 | 1 | 1 | 1 | 0 | 0 | 0 | 0 |
|  |  |  |  |  |  |  |  |  |
| **DMC 2** |  |  |  |  |  |  |  |  |
| **Species** |  |  |  |  |  |  |  |  |
| *Aspergillus protuberus* | 0 | 0 | 0 | 0 | 0 | 0 | 1 | 1 |
| *Aureobasidium melanogenum* | 0 | 0 | 1 | 1 | 0 | 0 | 0 | 0 |
| *Aureobasidium namibiae* | 0 | 0 | 0 | 0 | 1 | 1 | 0 | 0 |
| *Cladosporium delicatulum* | 0 | 0 | 0 | 0 | 1 | 1 | 1 | 1 |
| *Cladosporium tenuissimum* | 0 | 1 | 0 | 0 | 0 | 0 | 0 | 0 |
| *Mycosphaerella tassiana* | 0 | 0 | 0 | 0 | 1 | 1 | 1 | 1 |
|  |  |  |  |  |  |  |  |  |
| **Genus** |  |  |  |  |  |  |  |  |
| *Mycosphaerella* | 0 | 0 | 0 | 0 | 1 | 1 | 1 | 1 |
|  |  |  |  |  |  |  |  |  |
| **DMC 3** |  |  |  |  |  |  |  |  |
| **Species** |  |  |  |  |  |  |  |  |
| *Cladosporium delicatulum* | 0 | 0 | 0 | 0 | 1 | 1 | 1 | 1 |
| *Fusarium cerealis* | 0 | 1 | 0 | 0 | 0 | 0 | 0 | 0 |
| *Fusarium culmorum* | 0 | 0 | 1 | 1 | 0 | 0 | 0 | 0 |
| *Penicillium aurantiogriseum* | 0 | 1 | 0 | 0 | 1 | 1 | 1 | 1 |
| *Rhizopus oryzae* | 0 | 0 | 1 | 1 | 0 | 0 | 0 | 0 |
|  |  |  |  |  |  |  |  |  |
| **Genus** |  |  |  |  |  |  |  |  |
| *Fusarium* | 1 | 1 | 1 | 1 | 1 | 1 | 0 | 0 |
| *Penicillium* | 1 | 1 | 1 | 1 | 1 | 1 | 1 | 1 |
|  |  |  |  |  |  |  |  |  |
| **DMC 4** |  |  |  |  |  |  |  |  |
| **Species** |  |  |  |  |  |  |  |  |
| *Aspergillus fischeri* | 0 | 0 | 0 | 0 | 1 | 1 | 0 | 0 |
| *Cladosporium delicatulum* | 0 | 0 | 0 | 0 | 0 | 0 | 1 | 1 |
| *Cladosporium tenuissimum* | 0 | 1 | 0 | 0 | 0 | 0 | 0 | 0 |
|  |  |  |  |  |  |  |  |  |
| **DMC 5** |  |  |  |  |  |  |  |  |
| **Species** |  |  |  |  |  |  |  |  |
| *Aspergillus intermedius* | 0 | 0 | 0 | 0 | 0 | 1 | 1 | 1 |
| *Aspergillus montevidensis* | 0 | 0 | 1 | 1 | 0 | 0 | 0 | 0 |
| *Cephalotrichum nanum* | 0 | 0 | 1 | 1 | 0 | 0 | 0 | 0 |
| *Cladosporium delicatulum* | 0 | 0 | 0 | 0 | 1 | 1 | 1 | 1 |
| *Penicillium gladioli* | 0 | 0 | 0 | 0 | 1 | 1 | 0 | 0 |
| *Penicillium halotolerans* | 1 | 1 | 0 | 0 | 0 | 0 | 0 | 0 |
| *Pseudallescheria boydii* | 0 | 0 | 0 | 0 | 1 | 1 | 1 | 1 |
| *Syncephalastrum monosporum* | 0 | 0 | 1 | 1 | 0 | 0 | 0 | 0 |
|  |  |  |  |  |  |  |  |  |
| **Genus** |  |  |  |  |  |  |  |  |
| *Penicillium* | 1 | 1 | 1 | 1 | 1 | 1 | 1 | 1 |
| *Pseudallescheria* | 0 | 0 | 0 | 0 | 1 | 1 | 1 | 1 |
|  |  |  |  |  |  |  |  |  |
| **DMC 6** |  |  |  |  |  |  |  |  |
| **Species** |  |  |  |  |  |  |  |  |
| *Aspergillus candidus* | 0 | 0 | 0 | 0 | 1 | 1 | 0 | 0 |
|  |  |  |  |  |  |  |  |  |
| **DMC 7** |  |  |  |  |  |  |  |  |
| **Species** |  |  |  |  |  |  |  |  |
| *Arthrinium malaysianum* | 0 | 0 | 0 | 0 | 0 | 0 | 1 | 1 |
| *Aspergillus fischeri* | 0 | 0 | 0 | 0 | 1 | 1 | 0 | 0 |
| *Aspergillus fumigatus* | 1 | 1 | 1 | 1 | 0 | 0 | 0 | 0 |
| *Mycosphaerella tassiana* | 0 | 0 | 0 | 0 | 1 | 1 | 1 | 1 |
| *Penicillium aurantiogriseum* | 0 | 1 | 0 | 0 | 1 | 1 | 1 | 1 |
| *Purpureocillium lilacinum* | 0 | 1 | 1 | 1 | 1 | 1 | 1 | 1 |
| *Rhodotorula mucilaginosa* | 1 | 1 | 1 | 1 | 1 | 1 | 0 | 0 |
|  |  |  |  |  |  |  |  |  |
| **Genus** |  |  |  |  |  |  |  |  |
| *Aspergillus* | 1 | 1 | 1 | 1 | 1 | 1 | 1 | 1 |
| *Mycosphaerella* | 0 | 0 | 0 | 0 | 1 | 1 | 1 | 1 |
| *Penicillium* | 1 | 1 | 1 | 1 | 1 | 1 | 1 | 1 |
| *Purpureocillium* | 1 | 1 | 1 | 1 | 1 | 1 | 1 | 1 |
| *Rhodotorula* | 1 | 1 | 1 | 1 | 1 | 1 | 1 | 1 |
|  |  |  |  |  |  |  |  |  |
| **DMC 8** |  |  |  |  |  |  |  |  |
| **Species** |  |  |  |  |  |  |  |  |
| *Aspergillus aculeatus* | 0 | 0 | 0 | 0 | 0 | 0 | 1 | 1 |
| *Aspergillus brunneoviolaceus* | 0 | 0 | 1 | 1 | 0 | 0 | 0 | 0 |
| *Aspergillus japonicus* | 0 | 0 | 0 | 0 | 0 | 1 | 0 | 0 |
| *Aspergillus uvarum* | 0 | 1 | 0 | 0 | 0 | 0 | 0 | 0 |
| *Cephalotrichum nanum* | 0 | 0 | 1 | 1 | 0 | 0 | 0 | 0 |
| *Syncephalastrum monosporum* | 0 | 0 | 1 | 1 | 0 | 0 | 0 | 0 |
|  |  |  |  |  |  |  |  |  |
| **DMC 9** |  |  |  |  |  |  |  |  |
| **Species** |  |  |  |  |  |  |  |  |
| *Cladosporium delicatulum* | 0 | 0 | 0 | 0 | 1 | 1 | 1 | 1 |
| *Rhizopus oryzae* | 0 | 0 | 1 | 1 | 0 | 0 | 0 | 0 |
| *Sporothrix globosa* | 0 | 0 | 0 | 0 | 1 | 1 | 0 | 0 |
|  |  |  |  |  |  |  |  |  |
| **DMC 10** |  |  |  |  |  |  |  |  |
| **Species** |  |  |  |  |  |  |  |  |
| *Alternaria tenuissima* | 0 | 0 | 0 | 0 | 1 | 1 | 1 | 1 |
| *Cladosporium delicatulum* | 0 | 0 | 0 | 0 | 0 | 0 | 1 | 1 |
| *Cladosporium tenuissimum* | 0 | 1 | 0 | 0 | 0 | 0 | 0 | 0 |
| *Penicillium citreonigrum* | 0 | 0 | 1 | 1 | 0 | 1 | 0 | 0 |
| *Penicillium citreosulfuratum* | 0 | 1 | 0 | 0 | 0 | 0 | 0 | 0 |
| *Rhodotorula dairenensis* | 0 | 0 | 0 | 0 | 1 | 1 | 0 | 0 |
|  |  |  |  |  |  |  |  |  |
| **Genus** |  |  |  |  |  |  |  |  |
| *Penicillium* | 1 | 1 | 1 | 1 | 1 | 1 | 1 | 1 |
|  |  |  |  |  |  |  |  |  |
| **DMC 11** |  |  |  |  |  |  |  |  |
| **Species** |  |  |  |  |  |  |  |  |
| *Cephalotrichum nanum* | 0 | 0 | 1 | 1 | 0 | 0 | 0 | 0 |
| *Cladosporium allicinum* | 0 | 1 | 0 | 0 | 0 | 0 | 0 | 0 |
| *Mycosphaerella tassiana* | 0 | 0 | 0 | 0 | 1 | 1 | 1 | 1 |
| *Sporothrix globosa* | 0 | 0 | 0 | 0 | 1 | 1 | 0 | 0 |
|  |  |  |  |  |  |  |  |  |
| **Genus** |  |  |  |  |  |  |  |  |
| *Mycosphaerella* | 0 | 0 | 0 | 0 | 1 | 1 | 1 | 1 |
|  |  |  |  |  |  |  |  |  |
| **DMC 12** |  |  |  |  |  |  |  |  |
| **Species** |  |  |  |  |  |  |  |  |
| *Arthrinium malaysianum* | 0 | 0 | 0 | 0 | 0 | 0 | 1 | 1 |
| *Mycosphaerella tassiana* | 0 | 0 | 0 | 0 | 1 | 1 | 1 | 1 |
|  |  |  |  |  |  |  |  |  |
| **Genus** |  |  |  |  |  |  |  |  |
| *Mycosphaerella* | 0 | 0 | 0 | 0 | 1 | 1 | 1 | 1 |
|  |  |  |  |  |  |  |  |  |
| **DMC 13** |  |  |  |  |  |  |  |  |
| **Species** |  |  |  |  |  |  |  |  |
| *Cladosporium delicatulum* | 0 | 0 | 0 | 0 | 0 | 0 | 1 | 1 |
| *Cladosporium tenuissimum* | 0 | 1 | 0 | 0 | 0 | 0 | 0 | 0 |
| *Penicillium gladioli* | 0 | 0 | 0 | 0 | 1 | 1 | 0 | 0 |
| *Penicillium halotolerans* | 1 | 1 | 0 | 0 | 0 | 0 | 0 | 0 |
| *Sarocladium dejongiae* | 0 | 0 | 0 | 0 | 1 | 1 | 1 | 1 |
|  |  |  |  |  |  |  |  |  |
| **Genus** |  |  |  |  |  |  |  |  |
| *Cladosporium* | 1 | 1 | 1 | 1 | 1 | 1 | 1 | 1 |
|  |  |  |  |  |  |  |  |  |
| **DMC 14** |  |  |  |  |  |  |  |  |
| **Species** |  |  |  |  |  |  |  |  |
| *Cephalotrichum nanum* | 0 | 0 | 1 | 1 | 0 | 0 | 0 | 0 |
| *Mycosphaerella tassiana* | 0 | 0 | 0 | 0 | 1 | 1 | 1 | 1 |
|  |  |  |  |  |  |  |  |  |
| **Genus** |  |  |  |  |  |  |  |  |
| *Mycosphaerella* | 0 | 0 | 0 | 0 | 1 | 1 | 1 | 1 |
|  |  |  |  |  |  |  |  |  |
| **DMC 15** |  |  |  |  |  |  |  |  |
| **Species** |  |  |  |  |  |  |  |  |
| *Aspergillus fischeri* | 0 | 0 | 0 | 0 | 1 | 1 | 0 | 0 |
| *Aspergillus fumigatus* | 1 | 1 | 1 | 1 | 0 | 0 | 0 | 0 |
| *Aureobasidium melanogenum* | 0 | 0 | 1 | 1 | 0 | 0 | 0 | 0 |
| *Aureobasidium namibiae* | 0 | 0 | 0 | 0 | 1 | 1 | 0 | 0 |
| *Mycosphaerella tassiana* | 0 | 0 | 0 | 0 | 1 | 1 | 1 | 1 |
|  |  |  |  |  |  |  |  |  |
| **Genus** |  |  |  |  |  |  |  |  |
| *Aspergillus* | 1 | 1 | 1 | 1 | 1 | 1 | 1 | 1 |
| *Mycosphaerella* | 0 | 0 | 0 | 0 | 1 | 1 | 1 | 1 |
|  |  |  |  |  |  |  |  |  |
| **DMC 16** |  |  |  |  |  |  |  |  |
| **Species** |  |  |  |  |  |  |  |  |
| *Cladosporium delicatulum* | 0 | 0 | 0 | 0 | 1 | 1 | 1 | 1 |
| *Penicillium paczoskii* | 0 | 0 | 0 | 0 | 1 | 1 | 0 | 0 |
|  |  |  |  |  |  |  |  |  |
| **DMC 17** |  |  |  |  |  |  |  |  |
| **Species** |  |  |  |  |  |  |  |  |
| *Cladosporium delicatulum* | 0 | 0 | 0 | 0 | 1 | 1 | 1 | 1 |
| *Penicillium paczoskii* | 0 | 0 | 0 | 0 | 1 | 1 | 0 | 0 |
| *Rhizopus oryzae* | 0 | 0 | 1 | 1 | 0 | 0 | 0 | 0 |
|  |  |  |  |  |  |  |  |  |
| **DMC 18** |  |  |  |  |  |  |  |  |
| **Species** |  |  |  |  |  |  |  |  |
| *Aspergillus fischeri* | 0 | 0 | 0 | 0 | 1 | 1 | 0 | 0 |
| *Aspergillus fumigatus* | 1 | 1 | 1 | 1 | 0 | 0 | 0 | 0 |
| *Aspergillus jensenii* | 0 | 1 | 0 | 0 | 0 | 0 | 0 | 0 |
| *Aspergillus protuberus* | 0 | 0 | 0 | 0 | 0 | 0 | 1 | 1 |
| *Cladosporium delicatulum* | 0 | 0 | 0 | 0 | 1 | 1 | 1 | 1 |
| *Cladosporium sphaerospermum* | 1 | 1 | 1 | 1 | 1 | 1 | 1 | 1 |
| *Cladosporium tenuissimum* | 0 | 1 | 0 | 0 | 0 | 0 | 0 | 0 |
| *Stachybotrys chartarum* | 0 | 1 | 1 | 1 | 0 | 0 | 1 | 1 |
|  |  |  |  |  |  |  |  |  |
| **Genus** |  |  |  |  |  |  |  |  |
| *Aspergillus* | 1 | 1 | 1 | 1 | 1 | 1 | 1 | 1 |
| *Cladosporium* | 1 | 1 | 1 | 1 | 1 | 1 | 1 | 1 |
| *Stachybotrys* | 1 | 1 | 1 | 1 | 0 | 0 | 1 | 1 |
|  |  |  |  |  |  |  |  |  |
| **DMC 19** |  |  |  |  |  |  |  |  |
| **Species** |  |  |  |  |  |  |  |  |
| *Cladosporium delicatulum* | 0 | 0 | 0 | 0 | 0 | 0 | 1 | 1 |
| *Cladosporium tenuissimum* | 0 | 1 | 0 | 0 | 0 | 0 | 0 | 0 |
| *Gliomastix tumulicola* | 0 | 0 | 0 | 0 | 1 | 1 | 0 | 0 |
|  |  |  |  |  |  |  |  |  |
| **Genus** |  |  |  |  |  |  |  |  |
| *Cladosporium* | 1 | 1 | 1 | 1 | 1 | 1 | 1 | 1 |
|  |  |  |  |  |  |  |  |  |
| **DMC 20** |  |  |  |  |  |  |  |  |
| **Species** |  |  |  |  |  |  |  |  |
| *Cladosporium delicatulum* | 0 | 0 | 0 | 0 | 1 | 1 | 1 | 1 |
|  |  |  |  |  |  |  |  |  |
| **DMC 21** |  |  |  |  |  |  |  |  |
| **Species** |  |  |  |  |  |  |  |  |
| *Alternaria tenuissima* | 0 | 0 | 0 | 0 | 1 | 1 | 1 | 1 |
| *Aspergillus fischeri* | 0 | 0 | 0 | 0 | 1 | 1 | 0 | 0 |
| *Cladosporium delicatulum* | 0 | 0 | 0 | 0 | 0 | 0 | 1 | 1 |
| *Cladosporium tenuissimum* | 0 | 1 | 0 | 0 | 0 | 0 | 0 | 0 |
| *Penicillium gladioli* | 0 | 0 | 0 | 0 | 1 | 1 | 0 | 0 |
|  |  |  |  |  |  |  |  |  |
| **Genus** |  |  |  |  |  |  |  |  |
| *Cladosporium* | 1 | 1 | 1 | 1 | 1 | 1 | 1 | 1 |
|  |  |  |  |  |  |  |  |  |
| **DMC 22** |  |  |  |  |  |  |  |  |
| **Species** |  |  |  |  |  |  |  |  |
| *Aspergillus protuberus* | 0 | 0 | 0 | 0 | 0 | 0 | 1 | 1 |
| *Rhizopus oryzae* | 0 | 0 | 1 | 1 | 0 | 0 | 0 | 0 |
|  |  |  |  |  |  |  |  |  |
| **DMC 23** |  |  |  |  |  |  |  |  |
| **Species** |  |  |  |  |  |  |  |  |
| *Aspergillus fischeri* | 0 | 0 | 0 | 0 | 1 | 1 | 0 | 0 |
| *Cladosporium delicatulum* | 0 | 0 | 0 | 0 | 1 | 1 | 1 | 1 |
| *Cladosporium tenuissimum* | 0 | 1 | 0 | 0 | 0 | 0 | 0 | 0 |
|  |  |  |  |  |  |  |  |  |
| **Genus** |  |  |  |  |  |  |  |  |
| *Cladosporium* | 1 | 1 | 1 | 1 | 1 | 1 | 1 | 1 |
|  |  |  |  |  |  |  |  |  |
| **DMC 24** |  |  |  |  |  |  |  |  |
| **Species** |  |  |  |  |  |  |  |  |
| *Sarocladium dejongiae* | 0 | 0 | 0 | 0 | 1 | 1 | 1 | 1 |
|  |  |  |  |  |  |  |  |  |
| **DMC 25** |  |  |  |  |  |  |  |  |
| **Species** |  |  |  |  |  |  |  |  |
| *Cladosporium delicatulum* | 0 | 0 | 0 | 0 | 0 | 0 | 1 | 1 |
| *Cladosporium tenuissimum* | 0 | 1 | 0 | 0 | 0 | 0 | 0 | 0 |
|  |  |  |  |  |  |  |  |  |
| **DMC 26** |  |  |  |  |  |  |  |  |
| **Species** |  |  |  |  |  |  |  |  |
| *Byssochlamys spectabilis* | 0 | 0 | 1 | 1 | 1 | 1 | 1 | 1 |
| *Cladosporium delicatulum* | 0 | 0 | 0 | 0 | 1 | 1 | 1 | 1 |
| *Cladosporium tenuissimum* | 0 | 1 | 0 | 0 | 0 | 0 | 0 | 0 |
| *Epicoccum nigrum* | 1 | 1 | 1 | 1 | 0 | 1 | 0 | 0 |
| *Fusarium cerealis* | 0 | 1 | 0 | 0 | 0 | 0 | 0 | 0 |
| *Fusarium culmorum* | 0 | 0 | 1 | 1 | 0 | 0 | 0 | 0 |
| *Lichtheimia corymbifera* | 1 | 1 | 1 | 1 | 1 | 1 | 1 | 1 |
| *Paecilomyces variotii* | 1 | 1 | 0 | 0 | 0 | 0 | 0 | 0 |
| *Penicillium corylophilum* | 0 | 0 | 1 | 1 | 0 | 1 | 1 | 1 |
| *Penicillium glabrum* | 0 | 0 | 1 | 1 | 0 | 0 | 0 | 0 |
| *Penicillium paczoskii* | 0 | 0 | 0 | 0 | 1 | 1 | 0 | 0 |
| *Penicillium rubefaciens* | 0 | 1 | 0 | 0 | 0 | 0 | 0 | 0 |
| *Penicillium section Aspergilloides (glabrum-clade)* | 0 | 1 | 0 | 0 | 0 | 0 | 0 | 0 |
| *Rhizopus arrhizus* | 0 | 1 | 0 | 0 | 1 | 1 | 1 | 1 |
| *Rhizopus oryzae* | 0 | 0 | 1 | 1 | 0 | 0 | 0 | 0 |
|  |  |  |  |  |  |  |  |  |
| **Genus** |  |  |  |  |  |  |  |  |
| *Byssochlamys* | 0 | 0 | 1 | 1 | 1 | 1 | 1 | 1 |
| *Epicoccum* | 1 | 1 | 1 | 1 | 1 | 1 | 0 | 0 |
| *Fusarium* | 1 | 1 | 1 | 1 | 1 | 1 | 0 | 0 |
| *Lichtheimia* | 1 | 1 | 1 | 1 | 1 | 1 | 1 | 1 |
| *Paecilomyces* | 1 | 1 | 0 | 0 | 0 | 0 | 0 | 0 |
| *Rhizopus* | 1 | 1 | 1 | 1 | 1 | 1 | 1 | 1 |
|  |  |  |  |  |  |  |  |  |
| **DMC 27** |  |  |  |  |  |  |  |  |
| **Species** |  |  |  |  |  |  |  |  |
| *Arthrinium malaysianum* | 0 | 0 | 0 | 0 | 0 | 0 | 1 | 1 |
| *Aspergillus protuberus* | 0 | 0 | 0 | 0 | 0 | 0 | 1 | 1 |
| *Cladosporium allicinum* | 0 | 1 | 0 | 0 | 0 | 0 | 0 | 0 |
| *Cladosporium herbarum* | 0 | 0 | 1 | 1 | 0 | 0 | 0 | 0 |
| *Mycosphaerella tassiana* | 0 | 0 | 0 | 0 | 1 | 1 | 1 | 1 |
| *Syncephalastrum monosporum* | 0 | 0 | 1 | 1 | 0 | 0 | 0 | 0 |
|  |  |  |  |  |  |  |  |  |
| **Genus** |  |  |  |  |  |  |  |  |
| *Cladosporium* | 1 | 1 | 1 | 1 | 0 | 0 | 0 | 0 |
| *Mycosphaerella* | 0 | 0 | 0 | 0 | 1 | 1 | 1 | 1 |
|  |  |  |  |  |  |  |  |  |
| **DMC 28** |  |  |  |  |  |  |  |  |
| **Species** |  |  |  |  |  |  |  |  |
| *Aspergillus protuberus* | 0 | 0 | 0 | 0 | 0 | 0 | 1 | 1 |
| *Cladosporium delicatulum* | 0 | 0 | 0 | 0 | 1 | 1 | 1 | 1 |
| *Cladosporium tenuissimum* | 0 | 1 | 0 | 0 | 0 | 0 | 0 | 0 |
| *Penicillium gladioli* | 0 | 0 | 0 | 0 | 1 | 1 | 0 | 0 |
|  |  |  |  |  |  |  |  |  |
| **Genus** |  |  |  |  |  |  |  |  |
| *Cladosporium* | 1 | 1 | 1 | 1 | 1 | 1 | 1 | 1 |
|  |  |  |  |  |  |  |  |  |
| **DMC 29** |  |  |  |  |  |  |  |  |
| **Species** |  |  |  |  |  |  |  |  |
| *Aspergillus fischeri* | 0 | 0 | 0 | 0 | 1 | 1 | 0 | 0 |
| *Cladosporium cladosporioides* | 0 | 0 | 1 | 1 | 0 | 0 | 0 | 0 |
| *Cladosporium delicatulum* | 0 | 0 | 0 | 0 | 0 | 0 | 1 | 1 |
| *Cladosporium tenuissimum* | 0 | 1 | 0 | 0 | 0 | 0 | 0 | 0 |
| *Pseudallescheria boydii* | 0 | 0 | 0 | 0 | 1 | 1 | 1 | 1 |
| *Scedosporium apiospermum* | 1 | 1 | 1 | 1 | 0 | 0 | 0 | 0 |
|  |  |  |  |  |  |  |  |  |
| **Genus** |  |  |  |  |  |  |  |  |
| *Cladosporium* | 1 | 1 | 1 | 1 | 1 | 1 | 1 | 1 |
| *Pseudallescheria* | 0 | 0 | 0 | 0 | 1 | 1 | 1 | 1 |
| *Scedosporium* | 1 | 1 | 1 | 1 | 0 | 0 | 0 | 0 |
|  |  |  |  |  |  |  |  |  |
| **DMC 30** |  |  |  |  |  |  |  |  |
| **Species** |  |  |  |  |  |  |  |  |
| *Aspergillus fischeri* | 0 | 0 | 0 | 0 | 1 | 1 | 0 | 0 |
| *Aspergillus fumigatus* | 1 | 1 | 1 | 1 | 0 | 0 | 0 | 0 |
| *Rhizopus oryzae* | 0 | 0 | 1 | 1 | 0 | 0 | 0 | 0 |
| *Sporothrix globosa* | 1 | 1 | 0 | 0 | 1 | 1 | 0 | 0 |
|  |  |  |  |  |  |  |  |  |
| **Genus** |  |  |  |  |  |  |  |  |
| *Aspergillus* | 1 | 1 | 1 | 1 | 1 | 1 | 1 | 1 |
| *Rhizopus* | 0 | 0 | 1 | 1 | 0 | 0 | 0 | 0 |
|  |  |  |  |  |  |  |  |  |
| **DMC 31** |  |  |  |  |  |  |  |  |
| **Species** |  |  |  |  |  |  |  |  |
| *Aspergillus fischeri* | 0 | 0 | 0 | 0 | 1 | 1 | 0 | 0 |
| *Cryptococcus neoformans* | 0 | 1 | 1 | 1 | 1 | 1 | 1 | 1 |
| *Microascus brevicaulis* | 0 | 0 | 0 | 0 | 1 | 1 | 1 | 1 |
| *Penicillium digitatum* | 1 | 1 | 1 | 1 | 0 | 0 | 0 | 0 |
| *Scopulariopsis brevicaulis* | 1 | 1 | 1 | 1 | 0 | 0 | 0 | 0 |
|  |  |  |  |  |  |  |  |  |
| **Genus** |  |  |  |  |  |  |  |  |
| *Cryptococcus* | 1 | 1 | 1 | 1 | 1 | 1 | 1 | 1 |
| *Microascus* | 0 | 0 | 0 | 0 | 1 | 1 | 1 | 1 |
| *Scopulariopsis* | 1 | 1 | 1 | 1 | 0 | 0 | 0 | 0 |
|  |  |  |  |  |  |  |  |  |
| **DMC 32** |  |  |  |  |  |  |  |  |
| **Species** |  |  |  |  |  |  |  |  |
| *Cladosporium delicatulum* | 0 | 0 | 0 | 0 | 1 | 1 | 1 | 1 |
| *Cladosporium tenuissimum* | 0 | 1 | 0 | 0 | 0 | 0 | 0 | 0 |
| *Mycosphaerella tassiana* | 0 | 0 | 0 | 0 | 1 | 1 | 1 | 1 |
|  |  |  |  |  |  |  |  |  |
| **Genus** |  |  |  |  |  |  |  |  |
| *Mycosphaerella* | 0 | 0 | 0 | 0 | 1 | 1 | 1 | 1 |
|  |  |  |  |  |  |  |  |  |
| **DMC 33** |  |  |  |  |  |  |  |  |
| **Species** |  |  |  |  |  |  |  |  |
| *Alternaria tenuissima* | 0 | 0 | 0 | 0 | 1 | 1 | 1 | 1 |
| *Aspergillus intermedius* | 0 | 0 | 0 | 0 | 0 | 1 | 1 | 1 |
| *Aspergillus montevidensis* | 0 | 0 | 1 | 1 | 0 | 0 | 0 | 0 |
| *Cladosporium delicatulum* | 0 | 0 | 0 | 0 | 1 | 1 | 1 | 1 |
|  |  |  |  |  |  |  |  |  |
| **DMC 34** |  |  |  |  |  |  |  |  |
| **Species** |  |  |  |  |  |  |  |  |
| *Cladosporium delicatulum* | 0 | 0 | 0 | 0 | 1 | 1 | 1 | 1 |
| *Sarocladium dejongiae* | 0 | 0 | 0 | 0 | 1 | 1 | 1 | 1 |
|  |  |  |  |  |  |  |  |  |
| **DMC 35** |  |  |  |  |  |  |  |  |
| **Species** |  |  |  |  |  |  |  |  |
| *Alternaria tenuissima* | 0 | 0 | 0 | 0 | 1 | 1 | 1 | 1 |
| *Mycosphaerella tassiana* | 0 | 0 | 0 | 0 | 1 | 1 | 1 | 1 |
| *Sarocladium dejongiae* | 0 | 0 | 0 | 0 | 1 | 1 | 1 | 1 |
|  |  |  |  |  |  |  |  |  |
| **Genus** |  |  |  |  |  |  |  |  |
| *Mycosphaerella* | 0 | 0 | 0 | 0 | 1 | 1 | 1 | 1 |
|  |  |  |  |  |  |  |  |  |
| **DMC 36** |  |  |  |  |  |  |  |  |
| **Species** |  |  |  |  |  |  |  |  |
| *Cladosporium delicatulum* | 0 | 0 | 0 | 0 | 1 | 1 | 1 | 1 |
| *Penicillium gladioli* | 0 | 0 | 0 | 0 | 1 | 1 | 0 | 0 |
|  |  |  |  |  |  |  |  |  |
| **DMC 37** |  |  |  |  |  |  |  |  |
| **Species** |  |  |  |  |  |  |  |  |
| *Cladosporium delicatulum* | 0 | 0 | 0 | 0 | 1 | 1 | 1 | 1 |
| *Penicillium chrysogenum* | 0 | 0 | 0 | 0 | 1 | 1 | 0 | 0 |
| *Penicillium gladioli* | 0 | 0 | 0 | 0 | 1 | 1 | 0 | 0 |

Table 12: Overview of correctly identified species using Illumina sequences and the ITS2 region for classification for each DMC. Each cell indicates whether the species was correctly identified at the species level (S), the genus level (G) or not identified (0) at any of these levels for that combination of database, software and method.

|  | **IHEM** | | | | **UNITE** | | | |
| --- | --- | --- | --- | --- | --- | --- | --- | --- |
|  | **BLAST** | | **MOTHUR** | | **BLAST** | | **MOTHUR** | |
| **Species - DMC** | **Strict** | **Loose** | **Wang** | **knn** | **Strict** | **Loose** | **Wang** | **knn** |
|  |  |  |  |  |  |  |  |  |
| **DMC 1** |  |  |  |  |  |  |  |  |
| *Aspergillus fumigatus* | G | S | S | S | S | S | G | G |
| *Aspergillus versicolor* | G | S | G | G | G | G | G | G |
| *Cladosporium sphaerospermum* | S | S | S | S | S | S | S | S |
| *Penicillium chrysogenum* | G | S | G | G | G | S | G | G |
| *Stachybotrys chartarum* | S | S | S | S | S | S | S | S |
|  |  |  |  |  |  |  |  |  |
| **DMC 2** |  |  |  |  |  |  |  |  |
| *Aspergillus versicolor* | G | S | G | G | G | G | G | G |
| *Aureobasidium pullulans* | S | S | S | S | S | S | S | S |
| *Cladosporium herbarum* | G | S | G | G | 0 | 0 | 0 | 0 |
| *Penicillium aurantiogriseum* | G | S | G | G | S | S | S | S |
| *Stachybotrys chartarum* | S | S | S | S | S | S | S | S |
|  |  |  |  |  |  |  |  |  |
| **DMC 3** |  |  |  |  |  |  |  |  |
| *Aspergillus ochraceus* | G | S | G | G | G | S | S | S |
| *Chaetomium globosum* | S | S | S | S | G | S | G | G |
| *Cladosporium cladosporioides* | G | S | S | S | G | S | G | G |
| *Rhizopus arrhizus* | S | S | G | G | S | S | S | S |
| *Saccharomyces cerevisiae* | S | S | S | S | G | G | G | G |
|  |  |  |  |  |  |  |  |  |
| **DMC 4** |  |  |  |  |  |  |  |  |
| *Aspergillus fumigatus* | 0 | S | S | S | S | S | G | G |
| *Chaetomium globosum* | S | S | S | S | G | S | G | G |
| *Cladosporium sphaerospermum* | S | S | S | S | S | S | S | S |
| *Memnoniella echinata* | S | S | S | S | 0 | 0 | 0 | 0 |
| *Penicillium chrysogenum* | G | S | G | G | G | S | G | G |
|  |  |  |  |  |  |  |  |  |
| **DMC 5** |  |  |  |  |  |  |  |  |
| *Aspergillus chevalieri* | G | S | G | G | G | G | G | G |
| *Cephalotrichum stemonitis* | S | S | G | G | S | S | S | S |
| *Cladosporium cladosporioides* | G | S | S | S | G | S | G | G |
| *Scedosporium apiospermum* | S | S | S | S | 0 | 0 | 0 | 0 |
| *Syncephalastrum racemosum* | 0 | 0 | 0 | 0 | 0 | 0 | 0 | 0 |
|  |  |  |  |  |  |  |  |  |
| **DMC 6** |  |  |  |  |  |  |  |  |
| *Aspergillus subalbidus* | G | S | S | S | G | S | G | G |
| *Exophiala jeanselmei* | S | S | S | S | 0 | 0 | G | G |
| *Saccharomyces cerevisiae* | S | S | S | S | S | S | G | G |
| *Sporobolomyces roseus* | S | S | S | S | S | S | S | S |
| *Torula herbarum* | 0 | 0 | 0 | 0 | G | G | G | G |
|  |  |  |  |  |  |  |  |  |
| **DMC 7** |  |  |  |  |  |  |  |  |
| *Apiospora montagnei* | S | S | S | S | 0 | 0 | G | G |
| *Cladosporium herbarum* | G | S | G | G | 0 | 0 | 0 | 0 |
| *Exophiala jeanselmei* | S | S | S | S | 0 | 0 | G | G |
| *Paecilomyces variotii* | S | S | S | S | 0 | S | S | S |
| *Scopulariopsis brevicaulis* | S | S | S | S | S | S | 0 | 0 |
|  |  |  |  |  |  |  |  |  |
| **DMC 8** |  |  |  |  |  |  |  |  |
| *Aspergillus floridensis* | G | S | G | G | G | G | G | G |
| *Beauveria bassiana* | S | S | S | S | 0 | S | S | S |
| *Cephalotrichum stemonitis* | S | S | G | G | S | S | S | S |
| *Cladosporium sphaerospermum* | S | S | S | S | S | S | S | S |
| *Syncephalastrum racemosum* | 0 | 0 | 0 | 0 | 0 | 0 | 0 | 0 |
|  |  |  |  |  |  |  |  |  |
| **DMC 9** |  |  |  |  |  |  |  |  |
| *Aspergillus restrictus* | S | S | S | S | S | S | S | S |
| *Cladosporium cladosporioides* | G | S | S | S | G | S | G | G |
| *Exophiala jeanselmei* | S | S | S | S | 0 | 0 | G | G |
| *Rhizopus arrhizus* | S | S | G | G | S | S | S | S |
| *Sporothrix schenckii* | S | S | S | S | S | S | S | S |
|  |  |  |  |  |  |  |  |  |
| **DMC 10** |  |  |  |  |  |  |  |  |
| *Alternaria alternata* | S | S | S | S | G | S | S | S |
| *Cladosporium sphaerospermum* | S | S | S | S | S | S | S | S |
| *Exophiala jeanselmei* | S | S | S | S | 0 | 0 | G | G |
| *Purpureocillium lilacinum* | G | S | S | S | 0 | 0 | G | G |
| *Rhodotorula mucilaginosa* | S | S | S | S | S | S | S | S |
|  |  |  |  |  |  |  |  |  |
| **DMC 11** |  |  |  |  |  |  |  |  |
| *Aspergillus restrictus* | S | S | S | S | S | S | S | S |
| *Cephalotrichum stemonitis* | S | S | G | G | S | S | S | S |
| *Cladosporium sphaerospermum* | S | S | S | S | S | S | S | S |
| *Lichtheimia corymbifera* | S | S | S | S | S | S | S | S |
| *Sporothrix schenckii* | S | S | S | S | S | S | S | S |
|  |  |  |  |  |  |  |  |  |
| **DMC 12** |  |  |  |  |  |  |  |  |
| *Apiospora montagnei* | S | S | S | S | 0 | 0 | G | G |
| *Aspergillus ustus* | G | S | G | G | G | S | G | G |
| *Cladosporium herbarum* | G | S | G | G | 0 | 0 | 0 | 0 |
| *Penicillium brevicompactum* | S | S | S | S | G | S | S | S |
| *Sporobolomyces roseus* | S | S | S | S | S | S | S | S |
|  |  |  |  |  |  |  |  |  |
| **DMC 13** |  |  |  |  |  |  |  |  |
| *Aspergillus ochraceus* | G | S | G | G | G | S | S | S |
| *Cryptococcus neoformans* | G | S | S | S | S | S | S | S |
| *Memnoniella echinata* | S | S | S | S | 0 | 0 | 0 | 0 |
| *Penicillium brevicompactum* | S | S | S | S | G | S | S | S |
| *Sarocladium strictum* | S | S | S | S | G | G | G | G |
|  |  |  |  |  |  |  |  |  |
| **DMC 14** |  |  |  |  |  |  |  |  |
| *Cephalotrichum stemonitis* | S | S | G | G | S | S | S | S |
| *Cladosporium herbarum* | G | S | G | G | 0 | 0 | 0 | 0 |
| *Fusarium culmorum* | G | S | S | S | 0 | G | G | G |
| *Penicillium digitatum* | S | S | S | S | 0 | 0 | G | G |
| *Rhodotorula mucilaginosa* | S | S | S | S | S | S | S | S |
|  |  |  |  |  |  |  |  |  |
| **DMC 15** |  |  |  |  |  |  |  |  |
| *Aureobasidium pullulans* | S | S | S | S | S | S | S | S |
| *Cladosporium herbarum* | G | S | G | G | 0 | 0 | 0 | 0 |
| *Penicillium digitatum* | S | S | S | S | 0 | 0 | G | G |
| *Purpureocillium lilacinum* | G | S | S | S | 0 | 0 | G | G |
| *Rhodotorula mucilaginosa* | S | S | S | S | S | S | S | S |
|  |  |  |  |  |  |  |  |  |
| **DMC 16** |  |  |  |  |  |  |  |  |
| *Cladosporium cladosporioides* | G | S | S | S | G | S | G | G |
| *Fusarium culmorum* | G | S | S | S | 0 | G | G | G |
| *Geotrichum candidum* | S | S | S | S | S | S | S | S |
| *Penicillium glabrum* | G | S | S | S | G | G | G | G |
| *Stachybotrys chartarum* | S | S | S | S | S | S | S | S |
|  |  |  |  |  |  |  |  |  |
| **DMC 17** |  |  |  |  |  |  |  |  |
| *Cladosporium cladosporioides* | G | S | S | S | G | S | G | G |
| *Epicoccum nigrum* | S | S | S | S | 0 | S | 0 | 0 |
| *Paecilomyces variotii* | S | S | S | S | 0 | S | S | S |
| *Penicillium glabrum* | G | S | S | S | G | G | G | G |
| *Rhizopus arrhizus* | S | S | G | G | S | S | S | S |
|  |  |  |  |  |  |  |  |  |
| **DMC 18** |  |  |  |  |  |  |  |  |
| *Cryptococcus neoformans* | G | S | S | S | S | S | S | S |
| *Epicoccum nigrum* | S | S | S | S | 0 | S | 0 | 0 |
| *Paecilomyces variotii* | S | S | S | S | 0 | S | S | S |
| *Penicillium citreonigrum* | S | S | S | S | S | S | S | S |
| *Penicillium corylophilum* | S | S | S | S | S | S | S | S |
|  |  |  |  |  |  |  |  |  |
| **DMC 19** |  |  |  |  |  |  |  |  |
| *Fusarium culmorum* | G | S | S | S | 0 | G | G | G |
| *Gliomastix murorum* | S | S | S | S | 0 | 0 | 0 | 0 |
| *Lichtheimia corymbifera* | S | S | S | S | S | S | S | S |
| *Penicillium chrysogenum* | G | S | G | G | G | S | G | G |
| *Penicillium corylophilum* | S | S | S | S | S | S | S | S |
|  |  |  |  |  |  |  |  |  |
| **DMC 20** |  |  |  |  |  |  |  |  |
| *Cladosporium cladosporioides* | G | S | S | S | G | S | G | G |
| *Penicillium chrysogenum* | G | S | G | G | G | S | G | G |
| *Phoma herbarum* | S | S | 0 | 0 | S | S | 0 | 0 |
| *Purpureocillium lilacinum* | G | S | S | S | 0 | 0 | G | G |
| *Saccharomyces cerevisiae* | S | S | S | S | S | S | G | G |
|  |  |  |  |  |  |  |  |  |
| **DMC 21** |  |  |  |  |  |  |  |  |
| *Alternaria alternata* | S | S | S | S | G | S | S | S |
| *Aspergillus fumigatus* | 0 | S | S | S | S | S | G | G |
| *Penicillium halotolerans* | G | S | G | G | G | S | G | G |
| *Trichothecium roseum* | S | S | S | S | S | S | S | S |
| *Tritirachium oryzae* | S | S | S | S | S | S | S | S |
|  |  |  |  |  |  |  |  |  |
| **DMC 22** |  |  |  |  |  |  |  |  |
| *Aspergillus versicolor* | G | S | G | G | G | G | G | G |
| *Cryptococcus neoformans* | G | S | S | S | S | S | S | S |
| *Penicillium digitatum* | S | S | S | S | 0 | 0 | G | G |
| *Rhizopus arrhizus* | S | S | G | G | S | S | S | S |
| *Scopulariopsis brevicaulis* | S | S | S | S | S | S | 0 | 0 |
|  |  |  |  |  |  |  |  |  |
| **DMC 23** |  |  |  |  |  |  |  |  |
| *Aspergillus fumigatus* | 0 | S | S | S | S | S | G | G |
| *Penicillium digitatum* | S | S | S | S | 0 | 0 | G | G |
| *Rhodotorula mucilaginosa* | S | S | S | S | S | S | S | S |
| *Scopulariopsis brevicaulis* | S | S | S | S | S | S | 0 | 0 |
| *Stachybotrys chartarum* | S | S | S | S | S | S | S | S |
|  |  |  |  |  |  |  |  |  |
| **DMC 24** |  |  |  |  |  |  |  |  |
| *Cladosporium sphaerospermum* | S | S | S | S | S | S | S | S |
| *Lichtheimia corymbifera* | S | S | S | S | S | S | S | S |
| *Penicillium brevicompactum* | S | S | S | S | G | S | S | S |
| *Sarocladium strictum* | S | S | S | S | G | G | G | G |
| *Stachybotrys chartarum* | S | S | S | S | S | S | S | S |
|  |  |  |  |  |  |  |  |  |
| **DMC 25** |  |  |  |  |  |  |  |  |
| *Cladosporium sphaerospermum* | S | S | S | S | S | S | S | S |
| *Cryptococcus neoformans* | G | S | S | S | S | S | S | S |
| *Lichtheimia corymbifera* | S | S | S | S | S | S | S | S |
| *Penicillium brevicompactum* | S | S | S | S | G | S | S | S |
| *Stachybotrys chartarum* | S | S | S | S | S | S | S | S |
|  |  |  |  |  |  |  |  |  |
| **DMC 26** |  |  |  |  |  |  |  |  |
| *Aspergillus ustus* | G | S | G | G | G | S | G | G |
| *Cladosporium sphaerospermum* | S | S | S | S | S | S | S | S |
| *Cryptococcus neoformans* | G | S | S | S | S | S | S | S |
| *Penicillium brevicompactum* | S | S | S | S | G | S | S | S |
| *Phoma herbarum* | S | S | 0 | 0 | S | S | 0 | 0 |
|  |  |  |  |  |  |  |  |  |
| **DMC 27** |  |  |  |  |  |  |  |  |
| *Apiospora montagnei* | S | S | S | S | 0 | 0 | G | G |
| *Aspergillus versicolor* | G | S | G | G | G | G | G | G |
| *Penicillium chrysogenum* | G | S | G | G | G | S | G | G |
| *Syncephalastrum racemosum* | G | G | G | G | 0 | 0 | G | G |
| *Tritirachium oryzae* | S | S | S | S | S | S | S | S |
|  |  |  |  |  |  |  |  |  |
| **DMC 28** |  |  |  |  |  |  |  |  |
| *Aspergillus versicolor* | G | S | G | G | G | G | G | G |
| *Cunninghamella echinulata* | 0 | 0 | S | S | 0 | 0 | S | S |
| *Penicillium halotolerans* | G | S | G | G | G | S | G | G |
| *Torula herbarum* | 0 | 0 | 0 | 0 | G | G | G | G |
| *Trichothecium roseum* | S | S | S | S | S | S | S | S |
|  |  |  |  |  |  |  |  |  |
| **DMC 29** |  |  |  |  |  |  |  |  |
| *Aspergillus fumigatus* | 0 | S | S | S | S | S | G | G |
| *Penicillium chrysogenum* | G | S | G | G | G | S | G | G |
| *Rhodotorula mucilaginosa* | S | S | S | S | S | S | S | S |
| *Thamnidium elegans* | S | S | S | S | S | S | S | S |
| *Trichothecium roseum* | S | S | S | S | S | S | S | S |
|  |  |  |  |  |  |  |  |  |
| **DMC 30** |  |  |  |  |  |  |  |  |
| *Beauveria bassiana* | S | S | S | S | 0 | S | S | S |
| *Cunninghamella echinulata* | 0 | 0 | S | S | 0 | 0 | S | S |
| *Penicillium chrysogenum* | G | S | G | G | G | S | G | G |
| *Sporothrix schenckii* | S | S | S | S | S | S | S | S |
| *Tritirachium oryzae* | S | S | S | S | S | S | S | S |
|  |  |  |  |  |  |  |  |  |
| **DMC 31** |  |  |  |  |  |  |  |  |
| *Aspergillus fumigatus* | 0 | S | S | S | S | S | G | G |
| *Penicillium aurantiogriseum* | G | S | G | G | S | S | S | S |
| *Purpureocillium lilacinum* | G | S | S | S | 0 | 0 | G | G |
| *Trichothecium roseum* | S | S | S | S | S | S | S | S |
| *Tritirachium oryzae* | S | S | S | S | S | S | S | S |
|  |  |  |  |  |  |  |  |  |
| **DMC 32** |  |  |  |  |  |  |  |  |
| *Cladosporium herbarum* | G | S | G | G | 0 | 0 | 0 | 0 |
| *Memnoniella echinata* | S | S | S | S | 0 | 0 | 0 | 0 |
| *Penicillium aurantiogriseum* | G | S | G | G | S | S | S | S |
| *Sporobolomyces roseus* | S | S | S | S | S | S | S | S |
| *Thamnidium elegans* | S | S | S | S | S | S | S | S |
|  |  |  |  |  |  |  |  |  |
| **DMC 33** |  |  |  |  |  |  |  |  |
| *Alternaria alternata* | S | S | S | S | G | S | S | S |
| *Aspergillus chevalieri* | G | S | G | G | G | G | G | G |
| *Cladosporium cladosporioides* | G | S | S | S | G | S | G | G |
| *Geotrichum candidum* | S | S | S | S | S | S | S | S |
| *Penicillium aurantiogriseum* | G | S | G | G | S | S | S | S |
|  |  |  |  |  |  |  |  |  |
| **DMC 34** |  |  |  |  |  |  |  |  |
| *Cladosporium cladosporioides* | G | S | S | S | G | S | G | G |
| *Cunninghamella echinulata* | 0 | 0 | S | S | 0 | 0 | S | S |
| *Penicillium chrysogenum* | G | S | G | G | G | S | G | G |
| *Penicillium citreonigrum* | S | S | S | S | S | S | S | S |
| *Sarocladium strictum* | S | S | S | S | G | G | G | G |
|  |  |  |  |  |  |  |  |  |
| **DMC 35** |  |  |  |  |  |  |  |  |
| *Alternaria alternata* | S | S | S | S | G | S | S | S |
| *Cladosporium herbarum* | G | S | G | G | 0 | 0 | 0 | 0 |
| *Fusarium culmorum* | G | S | S | S | 0 | G | G | G |
| *Penicillium aurantiogriseum* | G | S | G | G | S | S | S | S |
| *Sarocladium strictum* | S | S | S | S | G | G | G | G |
|  |  |  |  |  |  |  |  |  |
| **DMC 36** |  |  |  |  |  |  |  |  |
| *Aspergillus ustus* | G | S | G | G | G | S | G | G |
| *Cladosporium cladosporioides* | G | S | S | S | G | S | G | G |
| *Penicillium halotolerans* | G | S | G | G | G | S | G | G |
| *Saccharomyces cerevisiae* | S | S | S | S | G | G | G | G |
| *Stachybotrys chartarum* | S | S | S | S | S | S | S | S |
|  |  |  |  |  |  |  |  |  |
| **DMC 37** |  |  |  |  |  |  |  |  |
| *Aspergillus ustus* | G | S | G | G | G | S | G | G |
| *Cladosporium cladosporioides* | G | S | S | S | G | S | G | G |
| *Penicillium halotolerans* | G | S | G | G | G | S | G | G |
| *Saccharomyces cerevisiae* | S | S | S | S | G | G | G | G |
| *Stachybotrys chartarum* | S | S | S | S | S | S | S | S |

Table 13: Overview of false positives identifies using Illumina sequences and the ITS2 region for classification for each DMC. Each cell indicates whether the species was identified (1) or not (0) for that combination of database, software and method.

|  | **IHEM** | | | | **UNITE** | | | |
| --- | --- | --- | --- | --- | --- | --- | --- | --- |
|  | **BLAST** | | **MOTHUR** | | **BLAST** | | **MOTHUR** | |
| **Species - DMC** | **Strict** | **Loose** | **Wang** | **knn** | **Strict** | **Loose** | **Wang** | **knn** |
|  |  |  |  |  |  |  |  |  |
| **DMC 1** |  |  |  |  |  |  |  |  |
| **Species** |  |  |  |  |  |  |  |  |
| *Aspergillus puulaauensis* | 0 | 0 | 0 | 0 | 1 | 1 | 0 | 0 |
| *Cladosporium cladosporioides* | 0 | 0 | 1 | 1 | 0 | 1 | 0 | 0 |
| *Cladosporium perangustum* | 0 | 1 | 0 | 0 | 0 | 0 | 0 | 0 |
| *Cladosporium tenuissimum* | 0 | 0 | 0 | 0 | 0 | 0 | 1 | 1 |
|  |  |  |  |  |  |  |  |  |
| **DMC 2** |  |  |  |  |  |  |  |  |
| **Species** |  |  |  |  |  |  |  |  |
| *Aspergillus puulaauensis* | 0 | 0 | 0 | 0 | 1 | 1 | 0 | 0 |
| *Mycosphaerella tassiana* | 0 | 0 | 0 | 0 | 1 | 1 | 0 | 0 |
|  |  |  |  |  |  |  |  |  |
| **Genus** |  |  |  |  |  |  |  |  |
| *Mycosphaerella* | 0 | 0 | 0 | 0 | 0 | 1 | 0 | 0 |
|  |  |  |  |  |  |  |  |  |
| **DMC 3** |  |  |  |  |  |  |  |  |
| **Species** |  |  |  |  |  |  |  |  |
| *Cladosporium tenuissimum* | 0 | 0 | 0 | 0 | 0 | 0 | 1 | 1 |
| *Rhizopus oryzae* | 0 | 0 | 1 | 1 | 0 | 0 | 0 | 0 |
|  |  |  |  |  |  |  |  |  |
| **DMC 4** |  |  |  |  |  |  |  |  |
|  |  |  |  |  |  |  |  |  |
| **DMC 5** |  |  |  |  |  |  |  |  |
| **Species** |  |  |  |  |  |  |  |  |
| *Aspergillus costiformis* | 0 | 0 | 0 | 0 | 0 | 1 | 0 | 0 |
| *Cladosporium tenuissimum* | 0 | 0 | 0 | 0 | 0 | 0 | 1 | 1 |
| *Pseudallescheria boydii* | 0 | 0 | 0 | 0 | 1 | 1 | 1 | 1 |
|  |  |  |  |  |  |  |  |  |
| **Genus** |  |  |  |  |  |  |  |  |
| *Pseudallescheria* | 0 | 0 | 0 | 0 | 1 | 1 | 1 | 1 |
|  |  |  |  |  |  |  |  |  |
| **DMC 6** |  |  |  |  |  |  |  |  |
|  |  |  |  |  |  |  |  |  |
| **DMC 7** |  |  |  |  |  |  |  |  |
| **Species** |  |  |  |  |  |  |  |  |
| *Arthrinium malaysianum* | 0 | 0 | 0 | 0 | 0 | 0 | 1 | 1 |
| *Mycosphaerella tassiana* | 0 | 0 | 0 | 0 | 1 | 1 | 0 | 0 |
|  |  |  |  |  |  |  |  |  |
| **Genus** |  |  |  |  |  |  |  |  |
| *Mycosphaerella* | 0 | 0 | 0 | 0 | 0 | 1 | 0 | 0 |
|  |  |  |  |  |  |  |  |  |
| **DMC 8** |  |  |  |  |  |  |  |  |
| **Species** |  |  |  |  |  |  |  |  |
| *Aspergillus aculeatus* | 0 | 0 | 0 | 0 | 0 | 1 | 0 | 0 |
| *Aspergillus brunneoviolaceus* | 0 | 0 | 1 | 1 | 0 | 0 | 0 | 0 |
| *Cladosporium cladosporioides* | 0 | 0 | 1 | 1 | 0 | 1 | 0 | 0 |
| *Cladosporium perangustum* | 0 | 1 | 0 | 0 | 0 | 0 | 0 | 0 |
| *Cladosporium tenuissimum* | 0 | 0 | 0 | 0 | 0 | 0 | 1 | 1 |
|  |  |  |  |  |  |  |  |  |
| **DMC 9** |  |  |  |  |  |  |  |  |
| **Species** |  |  |  |  |  |  |  |  |
| *Cladosporium tenuissimum* | 0 | 0 | 0 | 0 | 0 | 0 | 1 | 1 |
| *Rhizopus oryzae* | 0 | 0 | 1 | 1 | 0 | 0 | 0 | 0 |
|  |  |  |  |  |  |  |  |  |
| **DMC 10** |  |  |  |  |  |  |  |  |
|  |  |  |  |  |  |  |  |  |
| **DMC 11** |  |  |  |  |  |  |  |  |
|  |  |  |  |  |  |  |  |  |
| **DMC 12** |  |  |  |  |  |  |  |  |
| **Species** |  |  |  |  |  |  |  |  |
| *Arthrinium malaysianum* | 0 | 0 | 0 | 0 | 0 | 0 | 1 | 1 |
| *Aspergillus puniceus* | 0 | 0 | 1 | 1 | 0 | 0 | 0 | 0 |
| *Mycosphaerella tassiana* | 0 | 0 | 0 | 0 | 1 | 1 | 0 | 0 |
|  |  |  |  |  |  |  |  |  |
| **Genus** |  |  |  |  |  |  |  |  |
| *Mycosphaerella* | 0 | 0 | 0 | 0 | 0 | 1 | 0 | 0 |
|  |  |  |  |  |  |  |  |  |
| **DMC 13** |  |  |  |  |  |  |  |  |
| **Species** |  |  |  |  |  |  |  |  |
| *Sarocladium dejongiae* | 0 | 0 | 0 | 0 | 1 | 1 | 1 | 1 |
|  |  |  |  |  |  |  |  |  |
| **DMC 14** |  |  |  |  |  |  |  |  |
| **Species** |  |  |  |  |  |  |  |  |
| *Fusarium asiaticum* | 0 | 0 | 0 | 0 | 0 | 1 | 0 | 0 |
| *Mycosphaerella tassiana* | 0 | 0 | 0 | 0 | 1 | 1 | 0 | 0 |
|  |  |  |  |  |  |  |  |  |
| **Genus** |  |  |  |  |  |  |  |  |
| *Mycosphaerella* | 0 | 0 | 0 | 0 | 0 | 1 | 0 | 0 |
|  |  |  |  |  |  |  |  |  |
| **DMC 15** |  |  |  |  |  |  |  |  |
| **Species** |  |  |  |  |  |  |  |  |
| *Mycosphaerella tassiana* | 0 | 0 | 0 | 0 | 1 | 1 | 0 | 0 |
|  |  |  |  |  |  |  |  |  |
| **Genus** |  |  |  |  |  |  |  |  |
| *Mycosphaerella* | 0 | 0 | 0 | 0 | 0 | 1 | 0 | 0 |
|  |  |  |  |  |  |  |  |  |
| **DMC 16** |  |  |  |  |  |  |  |  |
| **Species** |  |  |  |  |  |  |  |  |
| *Cladosporium tenuissimum* | 0 | 0 | 0 | 0 | 0 | 0 | 1 | 1 |
| *Fusarium asiaticum* | 0 | 0 | 0 | 0 | 0 | 1 | 0 | 0 |
| *Penicillium thomii* | 0 | 0 | 0 | 0 | 0 | 1 | 0 | 0 |
|  |  |  |  |  |  |  |  |  |
| **DMC 17** |  |  |  |  |  |  |  |  |
| **Species** |  |  |  |  |  |  |  |  |
| *Cladosporium tenuissimum* | 0 | 0 | 0 | 0 | 0 | 0 | 1 | 1 |
| *Penicillium thomii* | 0 | 0 | 0 | 0 | 0 | 1 | 0 | 0 |
| *Rhizopus oryzae* | 0 | 0 | 1 | 1 | 0 | 0 | 0 | 0 |
|  |  |  |  |  |  |  |  |  |
| **DMC 18** |  |  |  |  |  |  |  |  |
|  |  |  |  |  |  |  |  |  |
| **DMC 19** |  |  |  |  |  |  |  |  |
| **Species** |  |  |  |  |  |  |  |  |
| *Fusarium asiaticum* | 0 | 0 | 0 | 0 | 0 | 1 | 0 | 0 |
|  |  |  |  |  |  |  |  |  |
| **DMC 20** |  |  |  |  |  |  |  |  |
| **Species** |  |  |  |  |  |  |  |  |
| *Cladosporium tenuissimum* | 0 | 0 | 0 | 0 | 0 | 0 | 1 | 1 |
|  |  |  |  |  |  |  |  |  |
| **DMC 21** |  |  |  |  |  |  |  |  |
|  |  |  |  |  |  |  |  |  |
| **DMC 22** |  |  |  |  |  |  |  |  |
| **Species** |  |  |  |  |  |  |  |  |
| *Aspergillus puulaauensis* | 0 | 0 | 0 | 0 | 1 | 1 | 0 | 0 |
| *Rhizopus oryzae* | 0 | 0 | 1 | 1 | 0 | 0 | 0 | 0 |
|  |  |  |  |  |  |  |  |  |
| **DMC 23** |  |  |  |  |  |  |  |  |
|  |  |  |  |  |  |  |  |  |
| **DMC 24** |  |  |  |  |  |  |  |  |
| **Species** |  |  |  |  |  |  |  |  |
| *Sarocladium dejongiae* | 0 | 0 | 0 | 0 | 1 | 1 | 1 | 1 |
|  |  |  |  |  |  |  |  |  |
| **DMC 25** |  |  |  |  |  |  |  |  |
|  |  |  |  |  |  |  |  |  |
| **DMC 26** |  |  |  |  |  |  |  |  |
| **Species** |  |  |  |  |  |  |  |  |
| *Aspergillus puniceus* | 0 | 0 | 1 | 1 | 0 | 0 | 0 | 0 |
|  |  |  |  |  |  |  |  |  |
| **DMC 27** |  |  |  |  |  |  |  |  |
| **Species** |  |  |  |  |  |  |  |  |
| *Arthrinium malaysianum* | 0 | 0 | 0 | 0 | 0 | 0 | 1 | 1 |
| *Aspergillus puulaauensis* | 0 | 0 | 0 | 0 | 1 | 1 | 0 | 0 |
| *Syncephalastrum monosporum* | 1 | 1 | 1 | 1 | 0 | 0 | 0 | 0 |
|  |  |  |  |  |  |  |  |  |
| **DMC 28** |  |  |  |  |  |  |  |  |
| **Species** |  |  |  |  |  |  |  |  |
| *Aspergillus puulaauensis* | 0 | 0 | 0 | 0 | 1 | 1 | 0 | 0 |
|  |  |  |  |  |  |  |  |  |
| **DMC 29** |  |  |  |  |  |  |  |  |
|  |  |  |  |  |  |  |  |  |
| **DMC 30** |  |  |  |  |  |  |  |  |
|  |  |  |  |  |  |  |  |  |
| **DMC 31** |  |  |  |  |  |  |  |  |
|  |  |  |  |  |  |  |  |  |
| **DMC 32** |  |  |  |  |  |  |  |  |
| **Species** |  |  |  |  |  |  |  |  |
| *Mycosphaerella tassiana* | 0 | 0 | 0 | 0 | 1 | 1 | 0 | 0 |
|  |  |  |  |  |  |  |  |  |
| **Genus** |  |  |  |  |  |  |  |  |
| *Mycosphaerella* | 0 | 0 | 0 | 0 | 0 | 1 | 0 | 0 |
|  |  |  |  |  |  |  |  |  |
| **DMC 33** |  |  |  |  |  |  |  |  |
| **Species** |  |  |  |  |  |  |  |  |
| *Aspergillus costiformis* | 0 | 0 | 0 | 0 | 0 | 1 | 0 | 0 |
| *Cladosporium tenuissimum* | 0 | 0 | 0 | 0 | 0 | 0 | 1 | 1 |
|  |  |  |  |  |  |  |  |  |
| **DMC 34** |  |  |  |  |  |  |  |  |
| **Species** |  |  |  |  |  |  |  |  |
| *Cladosporium tenuissimum* | 0 | 0 | 0 | 0 | 0 | 0 | 1 | 1 |
| *Sarocladium dejongiae* | 0 | 0 | 0 | 0 | 1 | 1 | 1 | 1 |
|  |  |  |  |  |  |  |  |  |
| **DMC 35** |  |  |  |  |  |  |  |  |
| **Species** |  |  |  |  |  |  |  |  |
| *Fusarium asiaticum* | 0 | 0 | 0 | 0 | 0 | 1 | 0 | 0 |
| *Mycosphaerella tassiana* | 0 | 0 | 0 | 0 | 1 | 1 | 0 | 0 |
| *Sarocladium dejongiae* | 0 | 0 | 0 | 0 | 1 | 1 | 1 | 1 |
|  |  |  |  |  |  |  |  |  |
| **Genus** |  |  |  |  |  |  |  |  |
| *Mycosphaerella* | 0 | 0 | 0 | 0 | 0 | 1 | 0 | 0 |
|  |  |  |  |  |  |  |  |  |
| **DMC 36** |  |  |  |  |  |  |  |  |
| **Species** |  |  |  |  |  |  |  |  |
| *Aspergillus puniceus* | 0 | 0 | 1 | 1 | 0 | 0 | 0 | 0 |
| *Cladosporium tenuissimum* | 0 | 0 | 0 | 0 | 0 | 0 | 1 | 1 |
|  |  |  |  |  |  |  |  |  |
| **DMC 37** |  |  |  |  |  |  |  |  |
| **Species** |  |  |  |  |  |  |  |  |
| *Aspergillus puniceus* | 0 | 0 | 1 | 1 | 0 | 0 | 0 | 0 |
| *Cladosporium tenuissimum* | 0 | 0 | 0 | 0 | 0 | 0 | 1 | 1 |

Table 14: Overview of correctly identified species using Illumina sequences and the consensus approach for classification. Each cell indicates whether the species was correctly identified at the species level (S), the genus level (G) or not identified (0) at any of these levels for that combination of database, software and method.

|  | **IHEM** | | | | **UNITE** | | | |
| --- | --- | --- | --- | --- | --- | --- | --- | --- |
|  | **BLAST** | | **MOTHUR** | | **BLAST** | | **MOTHUR** | |
| **Species - DMC** | **Strict** | **Loose** | **Wang** | **knn** | **Strict** | **Loose** | **Wang** | **knn** |
|  |  |  |  |  |  |  |  |  |
| **DMC 1** |  |  |  |  |  |  |  |  |
| *Aspergillus fumigatus* | G | S | S | S | G | G | G | G |
| *Aspergillus versicolor* | G | S | G | G | G | G | G | G |
| *Cladosporium sphaerospermum* | S | S | S | S | S | S | S | S |
| *Penicillium chrysogenum* | G | S | G | G | G | S | G | G |
| *Stachybotrys chartarum* | G | S | S | S | 0 | 0 | S | S |
|  |  |  |  |  |  |  |  |  |
| **DMC 2** |  |  |  |  |  |  |  |  |
| *Aspergillus versicolor* | G | S | G | G | 0 | 0 | G | G |
| *Aureobasidium pullulans* | G | S | G | G | G | G | 0 | 0 |
| *Cladosporium herbarum* | G | S | G | G | 0 | 0 | 0 | 0 |
| *Penicillium aurantiogriseum* | G | S | G | G | S | S | S | S |
| *Stachybotrys chartarum* | G | S | S | S | 0 | 0 | S | S |
|  |  |  |  |  |  |  |  |  |
| **DMC 3** |  |  |  |  |  |  |  |  |
| *Aspergillus ochraceus* | G | S | G | G | G | S | G | G |
| *Chaetomium globosum* | S | S | S | S | G | S | G | G |
| *Cladosporium cladosporioides* | G | S | G | G | G | G | G | G |
| *Rhizopus arrhizus* | S | S | G | G | S | S | S | S |
| *Saccharomyces cerevisiae* | S | S | S | S | G | G | G | G |
|  |  |  |  |  |  |  |  |  |
| **DMC 4** |  |  |  |  |  |  |  |  |
| *Aspergillus fumigatus* | 0 | S | S | S | G | G | G | G |
| *Chaetomium globosum* | S | S | S | S | G | S | G | G |
| *Cladosporium sphaerospermum* | S | S | S | S | S | S | S | S |
| *Memnoniella echinata* | S | S | S | S | 0 | 0 | 0 | 0 |
| *Penicillium chrysogenum* | G | S | G | G | G | S | G | G |
|  |  |  |  |  |  |  |  |  |
| **DMC 5** |  |  |  |  |  |  |  |  |
| *Aspergillus chevalieri* | G | S | G | G | G | G | G | G |
| *Cephalotrichum stemonitis* | G | S | G | G | G | G | G | G |
| *Cladosporium cladosporioides* | G | S | G | G | G | G | G | G |
| *Scedosporium apiospermum* | S | S | S | S | 0 | 0 | 0 | 0 |
| *Syncephalastrum racemosum* | 0 | 0 | 0 | 0 | 0 | 0 | 0 | 0 |
|  |  |  |  |  |  |  |  |  |
| **DMC 6** |  |  |  |  |  |  |  |  |
| *Aspergillus subalbidus* | G | S | S | S | G | G | G | G |
| *Exophiala jeanselmei* | S | S | S | S | 0 | 0 | G | G |
| *Saccharomyces cerevisiae* | S | S | S | S | S | S | G | G |
| *Sporobolomyces roseus* | S | S | S | S | S | S | S | S |
| *Torula herbarum* | 0 | 0 | 0 | 0 | 0 | 0 | G | G |
|  |  |  |  |  |  |  |  |  |
| **DMC 7** |  |  |  |  |  |  |  |  |
| *Apiospora montagnei* | S | S | S | S | 0 | 0 | G | G |
| *Cladosporium herbarum* | G | S | G | G | 0 | 0 | 0 | 0 |
| *Exophiala jeanselmei* | S | S | S | S | 0 | 0 | G | G |
| *Paecilomyces variotii* | S | S | S | S | 0 | S | S | S |
| *Scopulariopsis brevicaulis* | S | S | S | S | S | S | 0 | 0 |
|  |  |  |  |  |  |  |  |  |
| **DMC 8** |  |  |  |  |  |  |  |  |
| *Aspergillus floridensis* | G | G | G | G | G | G | G | G |
| *Beauveria bassiana* | S | S | S | S | 0 | S | 0 | 0 |
| *Cephalotrichum stemonitis* | G | S | G | G | G | G | G | G |
| *Cladosporium sphaerospermum* | S | S | S | S | S | S | S | S |
| *Syncephalastrum racemosum* | 0 | 0 | 0 | 0 | 0 | 0 | 0 | 0 |
|  |  |  |  |  |  |  |  |  |
| **DMC 9** |  |  |  |  |  |  |  |  |
| *Aspergillus restrictus* | S | S | S | S | S | S | S | S |
| *Cladosporium cladosporioides* | G | S | G | G | G | G | G | G |
| *Exophiala jeanselmei* | S | S | S | S | 0 | 0 | G | G |
| *Rhizopus arrhizus* | S | S | G | G | S | S | S | S |
| *Sporothrix schenckii* | S | S | S | S | G | G | G | G |
|  |  |  |  |  |  |  |  |  |
| **DMC 10** |  |  |  |  |  |  |  |  |
| *Alternaria alternata* | G | S | G | G | G | G | G | G |
| *Cladosporium sphaerospermum* | S | S | S | S | S | S | S | S |
| *Exophiala jeanselmei* | S | S | S | S | 0 | 0 | G | G |
| *Purpureocillium lilacinum* | G | S | S | S | 0 | 0 | G | G |
| *Rhodotorula mucilaginosa* | S | S | S | S | S | S | G | G |
|  |  |  |  |  |  |  |  |  |
| **DMC 11** |  |  |  |  |  |  |  |  |
| *Aspergillus restrictus* | S | S | S | S | S | S | S | S |
| *Cephalotrichum stemonitis* | G | S | G | G | G | G | G | G |
| *Cladosporium sphaerospermum* | S | S | S | S | S | S | S | S |
| *Lichtheimia corymbifera* | S | S | S | S | S | S | S | S |
| *Sporothrix schenckii* | S | S | S | S | G | G | G | G |
|  |  |  |  |  |  |  |  |  |
| **DMC 12** |  |  |  |  |  |  |  |  |
| *Apiospora montagnei* | S | S | S | S | 0 | 0 | G | G |
| *Aspergillus ustus* | G | S | G | G | G | S | G | G |
| *Cladosporium herbarum* | G | S | G | G | 0 | 0 | 0 | 0 |
| *Penicillium brevicompactum* | S | S | S | S | G | S | G | G |
| *Sporobolomyces roseus* | S | S | S | S | S | S | S | S |
|  |  |  |  |  |  |  |  |  |
| **DMC 13** |  |  |  |  |  |  |  |  |
| *Aspergillus ochraceus* | G | S | G | G | G | S | G | G |
| *Cryptococcus neoformans* | G | S | S | S | S | S | S | S |
| *Memnoniella echinata* | S | S | S | S | 0 | 0 | 0 | 0 |
| *Penicillium brevicompactum* | S | S | S | S | G | S | G | G |
| *Sarocladium strictum* | S | S | S | S | G | G | G | G |
|  |  |  |  |  |  |  |  |  |
| **DMC 14** |  |  |  |  |  |  |  |  |
| *Cephalotrichum stemonitis* | G | S | G | G | G | G | G | G |
| *Cladosporium herbarum* | G | S | G | G | 0 | 0 | 0 | 0 |
| *Fusarium culmorum* | G | S | S | S | 0 | G | 0 | 0 |
| *Penicillium digitatum* | S | S | S | S | 0 | 0 | G | G |
| *Rhodotorula mucilaginosa* | S | S | S | S | S | S | G | G |
|  |  |  |  |  |  |  |  |  |
| **DMC 15** |  |  |  |  |  |  |  |  |
| *Aureobasidium pullulans* | G | S | G | G | S | S | 0 | 0 |
| *Cladosporium herbarum* | G | S | G | G | 0 | 0 | 0 | 0 |
| *Penicillium digitatum* | S | S | S | S | 0 | 0 | G | G |
| *Purpureocillium lilacinum* | G | S | S | S | 0 | 0 | G | G |
| *Rhodotorula mucilaginosa* | S | S | S | S | S | S | G | G |
|  |  |  |  |  |  |  |  |  |
| **DMC 16** |  |  |  |  |  |  |  |  |
| *Cladosporium cladosporioides* | G | S | G | G | G | G | G | G |
| *Fusarium culmorum* | G | S | S | S | 0 | G | 0 | 0 |
| *Geotrichum candidum* | S | S | S | S | 0 | 0 | 0 | 0 |
| *Penicillium glabrum* | G | S | S | S | G | G | G | G |
| *Stachybotrys chartarum* | G | S | S | S | 0 | 0 | S | S |
|  |  |  |  |  |  |  |  |  |
| **DMC 17** |  |  |  |  |  |  |  |  |
| *Cladosporium cladosporioides* | G | S | G | G | G | G | G | G |
| *Epicoccum nigrum* | S | S | S | S | 0 | S | 0 | 0 |
| *Paecilomyces variotii* | S | S | S | S | 0 | S | S | S |
| *Penicillium glabrum* | G | S | S | S | G | G | G | G |
| *Rhizopus arrhizus* | S | S | G | G | S | S | S | S |
|  |  |  |  |  |  |  |  |  |
| **DMC 18** |  |  |  |  |  |  |  |  |
| *Cryptococcus neoformans* | G | S | S | S | S | S | S | S |
| *Epicoccum nigrum* | S | S | S | S | 0 | S | 0 | 0 |
| *Paecilomyces variotii* | S | S | S | S | 0 | S | S | S |
| *Penicillium citreonigrum* | G | S | S | S | G | S | G | G |
| *Penicillium corylophilum* | G | S | S | S | G | S | S | S |
|  |  |  |  |  |  |  |  |  |
| **DMC 19** |  |  |  |  |  |  |  |  |
| *Fusarium culmorum* | G | S | S | S | 0 | G | 0 | 0 |
| *Gliomastix murorum* | S | S | S | S | 0 | 0 | 0 | 0 |
| *Lichtheimia corymbifera* | S | S | S | S | S | S | S | S |
| *Penicillium chrysogenum* | G | S | G | G | G | S | G | G |
| *Penicillium corylophilum* | G | S | S | S | G | S | S | S |
|  |  |  |  |  |  |  |  |  |
| **DMC 20** |  |  |  |  |  |  |  |  |
| *Cladosporium cladosporioides* | G | S | G | G | G | G | G | G |
| *Penicillium chrysogenum* | G | S | G | G | G | S | G | G |
| *Phoma herbarum* | S | S | 0 | 0 | S | S | 0 | 0 |
| *Purpureocillium lilacinum* | G | S | S | S | 0 | 0 | G | G |
| *Saccharomyces cerevisiae* | S | S | S | S | S | S | G | G |
|  |  |  |  |  |  |  |  |  |
| **DMC 21** |  |  |  |  |  |  |  |  |
| *Alternaria alternata* | G | S | G | G | G | G | G | G |
| *Aspergillus fumigatus* | 0 | S | S | S | G | G | G | G |
| *Penicillium halotolerans* | G | S | G | G | G | G | G | G |
| *Trichothecium roseum* | S | S | S | S | S | S | S | S |
| *Tritirachium oryzae* | S | S | S | S | S | S | S | S |
|  |  |  |  |  |  |  |  |  |
| **DMC 22** |  |  |  |  |  |  |  |  |
| *Aspergillus versicolor* | G | S | G | G | 0 | 0 | G | G |
| *Cryptococcus neoformans* | G | S | S | S | S | S | S | S |
| *Penicillium digitatum* | S | S | S | S | 0 | 0 | G | G |
| *Rhizopus arrhizus* | S | S | G | G | S | S | S | S |
| *Scopulariopsis brevicaulis* | S | S | S | S | S | S | 0 | 0 |
|  |  |  |  |  |  |  |  |  |
| **DMC 23** |  |  |  |  |  |  |  |  |
| *Aspergillus fumigatus* | 0 | S | S | S | G | G | G | G |
| *Penicillium digitatum* | S | S | S | S | 0 | 0 | G | G |
| *Rhodotorula mucilaginosa* | S | S | S | S | S | S | G | G |
| *Scopulariopsis brevicaulis* | S | S | S | S | S | S | 0 | 0 |
| *Stachybotrys chartarum* | G | S | S | S | 0 | 0 | S | S |
|  |  |  |  |  |  |  |  |  |
| **DMC 24** |  |  |  |  |  |  |  |  |
| *Cladosporium sphaerospermum* | S | S | S | S | S | S | S | S |
| *Lichtheimia corymbifera* | S | S | S | S | S | S | S | S |
| *Penicillium brevicompactum* | S | S | S | S | G | S | G | G |
| *Sarocladium strictum* | S | S | S | S | G | G | G | G |
| *Stachybotrys chartarum* | G | S | S | S | 0 | 0 | S | S |
|  |  |  |  |  |  |  |  |  |
| **DMC 25** |  |  |  |  |  |  |  |  |
| *Cladosporium sphaerospermum* | S | S | S | S | S | S | S | S |
| *Cryptococcus neoformans* | G | S | S | S | S | S | S | S |
| *Lichtheimia corymbifera* | S | S | S | S | S | S | S | S |
| *Penicillium brevicompactum* | S | S | S | S | G | S | G | G |
| *Stachybotrys chartarum* | G | S | S | S | 0 | 0 | S | S |
|  |  |  |  |  |  |  |  |  |
| **DMC 26** |  |  |  |  |  |  |  |  |
| *Aspergillus ustus* | G | S | G | G | G | S | G | G |
| *Cladosporium sphaerospermum* | S | S | S | S | S | S | S | S |
| *Cryptococcus neoformans* | G | S | S | S | S | S | S | S |
| *Penicillium brevicompactum* | S | S | S | S | G | S | G | G |
| *Phoma herbarum* | S | S | 0 | 0 | S | S | 0 | 0 |
|  |  |  |  |  |  |  |  |  |
| **DMC 27** |  |  |  |  |  |  |  |  |
| *Apiospora montagnei* | S | S | S | S | 0 | 0 | G | G |
| *Aspergillus versicolor* | G | S | G | G | 0 | 0 | G | G |
| *Penicillium chrysogenum* | G | S | G | G | G | S | G | G |
| *Syncephalastrum racemosum* | 0 | 0 | G | G | 0 | 0 | G | G |
| *Tritirachium oryzae* | S | S | S | S | S | S | S | S |
|  |  |  |  |  |  |  |  |  |
| **DMC 28** |  |  |  |  |  |  |  |  |
| *Aspergillus versicolor* | G | S | G | G | 0 | 0 | G | G |
| *Cunninghamella echinulata* | 0 | 0 | S | S | 0 | 0 | S | S |
| *Penicillium halotolerans* | G | S | G | G | G | G | G | G |
| *Torula herbarum* | 0 | 0 | 0 | 0 | G | G | G | G |
| *Trichothecium roseum* | S | S | S | S | S | S | S | S |
|  |  |  |  |  |  |  |  |  |
| **DMC 29** |  |  |  |  |  |  |  |  |
| *Aspergillus fumigatus* | 0 | S | S | S | G | G | G | G |
| *Penicillium chrysogenum* | G | S | G | G | G | S | G | G |
| *Rhodotorula mucilaginosa* | S | S | S | S | S | S | G | G |
| *Thamnidium elegans* | S | S | S | S | S | S | S | S |
| *Trichothecium roseum* | S | S | S | S | S | S | S | S |
|  |  |  |  |  |  |  |  |  |
| **DMC 30** |  |  |  |  |  |  |  |  |
| *Beauveria bassiana* | S | S | S | S | 0 | S | 0 | 0 |
| *Cunninghamella echinulata* | 0 | 0 | S | S | 0 | 0 | S | S |
| *Penicillium chrysogenum* | G | S | G | G | G | S | G | G |
| *Sporothrix schenckii* | S | S | S | S | G | G | G | G |
| *Tritirachium oryzae* | S | S | S | S | S | S | S | S |
|  |  |  |  |  |  |  |  |  |
| **DMC 31** |  |  |  |  |  |  |  |  |
| *Aspergillus fumigatus* | 0 | S | S | S | G | G | G | G |
| *Penicillium aurantiogriseum* | G | S | G | G | S | S | S | S |
| *Purpureocillium lilacinum* | G | S | S | S | 0 | 0 | G | G |
| *Trichothecium roseum* | S | S | S | S | S | S | S | S |
| *Tritirachium oryzae* | S | S | S | S | S | S | S | S |
|  |  |  |  |  |  |  |  |  |
| **DMC 32** |  |  |  |  |  |  |  |  |
| *Cladosporium herbarum* | G | S | G | G | 0 | 0 | 0 | 0 |
| *Memnoniella echinata* | S | S | S | S | 0 | 0 | 0 | 0 |
| *Penicillium aurantiogriseum* | G | S | G | G | S | S | S | S |
| *Sporobolomyces roseus* | S | S | S | S | S | S | S | S |
| *Thamnidium elegans* | S | S | S | S | S | S | S | S |
|  |  |  |  |  |  |  |  |  |
| **DMC 33** |  |  |  |  |  |  |  |  |
| *Alternaria alternata* | G | S | G | G | G | G | G | G |
| *Aspergillus chevalieri* | G | S | G | G | G | G | G | G |
| *Cladosporium cladosporioides* | G | S | G | G | G | G | G | G |
| *Geotrichum candidum* | S | S | S | S | 0 | 0 | 0 | 0 |
| *Penicillium aurantiogriseum* | G | S | G | G | S | S | S | S |
|  |  |  |  |  |  |  |  |  |
| **DMC 34** |  |  |  |  |  |  |  |  |
| *Cladosporium cladosporioides* | G | S | G | G | G | G | G | G |
| *Cunninghamella echinulata* | 0 | 0 | S | S | 0 | 0 | S | S |
| *Penicillium chrysogenum* | G | S | G | G | G | S | G | G |
| *Penicillium citreonigrum* | G | S | S | S | G | S | G | G |
| *Sarocladium strictum* | S | S | S | S | G | G | G | G |
|  |  |  |  |  |  |  |  |  |
| **DMC 35** |  |  |  |  |  |  |  |  |
| *Alternaria alternata* | G | S | G | G | G | G | G | G |
| *Cladosporium herbarum* | G | S | G | G | 0 | 0 | 0 | 0 |
| *Fusarium culmorum* | G | S | S | S | 0 | G | 0 | 0 |
| *Penicillium aurantiogriseum* | G | S | G | G | S | S | S | S |
| *Sarocladium strictum* | S | S | S | S | G | G | G | G |
|  |  |  |  |  |  |  |  |  |
| **DMC 36** |  |  |  |  |  |  |  |  |
| *Aspergillus ustus* | G | S | G | G | G | S | G | G |
| *Cladosporium cladosporioides* | G | S | G | G | G | G | G | G |
| *Penicillium halotolerans* | G | S | G | G | G | G | G | G |
| *Saccharomyces cerevisiae* | S | S | S | S | G | G | G | G |
| *Stachybotrys chartarum* | G | S | S | S | 0 | 0 | S | S |
|  |  |  |  |  |  |  |  |  |
| **DMC 37** |  |  |  |  |  |  |  |  |
| *Aspergillus ustus* | G | S | G | G | G | S | G | G |
| *Cladosporium cladosporioides* | G | S | G | G | G | G | G | G |
| *Penicillium halotolerans* | G | S | G | G | G | G | G | G |
| *Saccharomyces cerevisiae* | S | S | S | S | G | G | G | G |
| *Stachybotrys chartarum* | G | S | S | S | 0 | 0 | S | S |

Table 15: Overview of false positives identified using Illumina sequences and the consensus approach for classification. Each cell indicates whether the species was identified (1) or not (0) for that combination of database, software and method.

|  | **IHEM** | | | | **UNITE** | | | |
| --- | --- | --- | --- | --- | --- | --- | --- | --- |
|  | **BLAST** | | **MOTHUR** | | **BLAST** | | **MOTHUR** | |
| **Species - DMC** | **Strict** | **Loose** | **Wang** | **knn** | **Strict** | **Loose** | **Wang** | **knn** |
|  |  |  |  |  |  |  |  |  |
| **DMC 1** |  |  |  |  |  |  |  |  |
|  |  |  |  |  |  |  |  |  |
| **DMC 2** |  |  |  |  |  |  |  |  |
| **Species** |  |  |  |  |  |  |  |  |
| *Mycosphaerella tassiana* | 0 | 0 | 0 | 0 | 1 | 1 | 0 | 0 |
|  |  |  |  |  |  |  |  |  |
| **Genus** |  |  |  |  |  |  |  |  |
| *Mycosphaerella* | 0 | 0 | 0 | 0 | 0 | 1 | 0 | 0 |
|  |  |  |  |  |  |  |  |  |
| **DMC 3** |  |  |  |  |  |  |  |  |
| **Species** |  |  |  |  |  |  |  |  |
| *Rhizopus oryzae* | 0 | 0 | 1 | 1 | 0 | 0 | 0 | 0 |
|  |  |  |  |  |  |  |  |  |
| **DMC 4** |  |  |  |  |  |  |  |  |
|  |  |  |  |  |  |  |  |  |
| **DMC 5** |  |  |  |  |  |  |  |  |
| **Species** |  |  |  |  |  |  |  |  |
| *Pseudallescheria boydii* | 0 | 0 | 0 | 0 | 1 | 1 | 1 | 1 |
|  |  |  |  |  |  |  |  |  |
| **Genus** |  |  |  |  |  |  |  |  |
| *Pseudallescheria* | 0 | 0 | 0 | 0 | 1 | 1 | 1 | 1 |
|  |  |  |  |  |  |  |  |  |
| **DMC 6** |  |  |  |  |  |  |  |  |
|  |  |  |  |  |  |  |  |  |
| **DMC 7** |  |  |  |  |  |  |  |  |
| **Species** |  |  |  |  |  |  |  |  |
| *Arthrinium malaysianum* | 0 | 0 | 0 | 0 | 0 | 0 | 1 | 1 |
| *Mycosphaerella tassiana* | 0 | 0 | 0 | 0 | 1 | 1 | 0 | 0 |
|  |  |  |  |  |  |  |  |  |
| **Genus** |  |  |  |  |  |  |  |  |
| *Mycosphaerella* | 0 | 0 | 0 | 0 | 0 | 1 | 0 | 0 |
|  |  |  |  |  |  |  |  |  |
| **DMC 8** |  |  |  |  |  |  |  |  |
| **Species** |  |  |  |  |  |  |  |  |
| *Aspergillus brunneoviolaceus* | 0 | 0 | 1 | 1 | 0 | 0 | 0 | 0 |
|  |  |  |  |  |  |  |  |  |
| **DMC 9** |  |  |  |  |  |  |  |  |
| **Species** |  |  |  |  |  |  |  |  |
| *Rhizopus oryzae* | 0 | 0 | 1 | 1 | 0 | 0 | 0 | 0 |
|  |  |  |  |  |  |  |  |  |
| **DMC 10** |  |  |  |  |  |  |  |  |
|  |  |  |  |  |  |  |  |  |
| **DMC 11** |  |  |  |  |  |  |  |  |
|  |  |  |  |  |  |  |  |  |
| **DMC 12** |  |  |  |  |  |  |  |  |
| **Species** |  |  |  |  |  |  |  |  |
| *Arthrinium malaysianum* | 0 | 0 | 0 | 0 | 0 | 0 | 1 | 1 |
| *Mycosphaerella tassiana* | 0 | 0 | 0 | 0 | 1 | 1 | 0 | 0 |
|  |  |  |  |  |  |  |  |  |
| **Genus** |  |  |  |  |  |  |  |  |
| *Mycosphaerella* | 0 | 0 | 0 | 0 | 0 | 1 | 0 | 0 |
|  |  |  |  |  |  |  |  |  |
| **DMC 13** |  |  |  |  |  |  |  |  |
| **Species** |  |  |  |  |  |  |  |  |
| *Sarocladium dejongiae* | 0 | 0 | 0 | 0 | 1 | 1 | 1 | 1 |
|  |  |  |  |  |  |  |  |  |
| **DMC 14** |  |  |  |  |  |  |  |  |
| **Species** |  |  |  |  |  |  |  |  |
| *Mycosphaerella tassiana* | 0 | 0 | 0 | 0 | 1 | 1 | 0 | 0 |
|  |  |  |  |  |  |  |  |  |
| **Genus** |  |  |  |  |  |  |  |  |
| *Mycosphaerella* | 0 | 0 | 0 | 0 | 0 | 1 | 0 | 0 |
|  |  |  |  |  |  |  |  |  |
| **DMC 15** |  |  |  |  |  |  |  |  |
| **Species** |  |  |  |  |  |  |  |  |
| *Mycosphaerella tassiana* | 0 | 0 | 0 | 0 | 1 | 1 | 0 | 0 |
|  |  |  |  |  |  |  |  |  |
| **Genus** |  |  |  |  |  |  |  |  |
| *Mycosphaerella* | 0 | 0 | 0 | 0 | 0 | 1 | 0 | 0 |
|  |  |  |  |  |  |  |  |  |
| **DMC 16** |  |  |  |  |  |  |  |  |
|  |  |  |  |  |  |  |  |  |
| **DMC 17** |  |  |  |  |  |  |  |  |
| **Species** |  |  |  |  |  |  |  |  |
| *Rhizopus oryzae* | 0 | 0 | 1 | 1 | 0 | 0 | 0 | 0 |
|  |  |  |  |  |  |  |  |  |
| **DMC 18** |  |  |  |  |  |  |  |  |
|  |  |  |  |  |  |  |  |  |
| **DMC 19** |  |  |  |  |  |  |  |  |
|  |  |  |  |  |  |  |  |  |
| **DMC 20** |  |  |  |  |  |  |  |  |
|  |  |  |  |  |  |  |  |  |
| **DMC 21** |  |  |  |  |  |  |  |  |
|  |  |  |  |  |  |  |  |  |
| **DMC 22** |  |  |  |  |  |  |  |  |
| **Species** |  |  |  |  |  |  |  |  |
| *Rhizopus oryzae* | 0 | 0 | 1 | 1 | 0 | 0 | 0 | 0 |
|  |  |  |  |  |  |  |  |  |
| **DMC 23** |  |  |  |  |  |  |  |  |
|  |  |  |  |  |  |  |  |  |
| **DMC 24** |  |  |  |  |  |  |  |  |
| **Species** |  |  |  |  |  |  |  |  |
| *Sarocladium dejongiae* | 0 | 0 | 0 | 0 | 1 | 1 | 1 | 1 |
|  |  |  |  |  |  |  |  |  |
| **DMC 25** |  |  |  |  |  |  |  |  |
|  |  |  |  |  |  |  |  |  |
| **DMC 26** |  |  |  |  |  |  |  |  |
|  |  |  |  |  |  |  |  |  |
| **DMC 27** |  |  |  |  |  |  |  |  |
| **Species** |  |  |  |  |  |  |  |  |
| *Arthrinium malaysianum* | 0 | 0 | 0 | 0 | 0 | 0 | 1 | 1 |
| *Syncephalastrum monosporum* | 0 | 0 | 1 | 1 | 0 | 0 | 0 | 0 |
|  |  |  |  |  |  |  |  |  |
| **DMC 28** |  |  |  |  |  |  |  |  |
|  |  |  |  |  |  |  |  |  |
| **DMC 29** |  |  |  |  |  |  |  |  |
|  |  |  |  |  |  |  |  |  |
| **DMC 30** |  |  |  |  |  |  |  |  |
|  |  |  |  |  |  |  |  |  |
| **DMC 31** |  |  |  |  |  |  |  |  |
|  |  |  |  |  |  |  |  |  |
| **DMC 32** |  |  |  |  |  |  |  |  |
| **Species** |  |  |  |  |  |  |  |  |
| *Mycosphaerella tassiana* | 0 | 0 | 0 | 0 | 1 | 1 | 0 | 0 |
|  |  |  |  |  |  |  |  |  |
| **Genus** |  |  |  |  |  |  |  |  |
| *Mycosphaerella* | 0 | 0 | 0 | 0 | 0 | 1 | 0 | 0 |
|  |  |  |  |  |  |  |  |  |
| **DMC 33** |  |  |  |  |  |  |  |  |
|  |  |  |  |  |  |  |  |  |
| **DMC 34** |  |  |  |  |  |  |  |  |
| **Species** |  |  |  |  |  |  |  |  |
| *Sarocladium dejongiae* | 0 | 0 | 0 | 0 | 1 | 1 | 1 | 1 |
|  |  |  |  |  |  |  |  |  |
| **DMC 35** |  |  |  |  |  |  |  |  |
| **Species** |  |  |  |  |  |  |  |  |
| *Mycosphaerella tassiana* | 0 | 0 | 0 | 0 | 1 | 1 | 0 | 0 |
| *Sarocladium dejongiae* | 0 | 0 | 0 | 0 | 1 | 1 | 1 | 1 |
|  |  |  |  |  |  |  |  |  |
| **Genus** |  |  |  |  |  |  |  |  |
| *Mycosphaerella* | 0 | 0 | 0 | 0 | 0 | 1 | 0 | 0 |
|  |  |  |  |  |  |  |  |  |
| **DMC 36** |  |  |  |  |  |  |  |  |
|  |  |  |  |  |  |  |  |  |
| **DMC 37** |  |  |  |  |  |  |  |  |

Table 16: comparison of the ITS1 Blast results of the IHEM database to the original composition of the DMCs. The blast results in which multiple species were retrieved with equal e-value and percentage, are manually classified to a subgeneric level (section, series or species complex). NID: no identification.

|  |  |  |
| --- | --- | --- |
| **DMC species composition** | **Subgeneric classification** | **Top equivalent BLAST hits** |
| 1 |  |  |
| *Aspergillus versicolor* | Aspergillus sect. Nidulantes (series Versicolores) | A. jensenii + A. creber + A. sect. Nidulantes (versicolor-clade) |
| *Aspergillus fumigatus* | Aspergillus sect. Fumigati | A. fischeri + A. aureolus + A. fumigatus |
| *Cladosporium sphaerospermum* | Cladosporium sphaerospermum |  |
| *Stachybotrys chartarum* | Stachybotrys chartarum species complex | S. chlorohalonata + S. chartarum |
| *Penicillium chrysogenum* | Penicillium sect. Chrysogena (series Chrysogena) | P. chrysogenum + P. rubens |
| 2 |  |  |
| Aspergillus versicolor | Aspergillus sect. Nidulantes (series Versicolores) | A. jensenii + A. creber + versicolor-clade |
| Stachybotrys chartarum | Stachybotrys chartarum species complex | S. chlorohalonata + chartarum |
| Aureobasidium pullulans | Aureobasidium sp. | A. melanogenum + A. sp. + A. pullulans |
| Cladosporium herbarum | Cladosporium herbarum species complex | C. sinuosum + allicinum + herbarum |
| Penicillium aurantiogriseum | Penicillium sect. Fasciculata (series Viridicata) | P. freii + aurantiogriseum |
| 3 |  |  |
| Cladosporium cladosporioides | Cladosporium cladosporioides species complex | C. tenuissimum + inversicolor + cladosporioides complex |
| Aspergillus ochraceus | Aspergillus ochraceus |  |
| Chaetomium globosum | Chaetomium globosum |  |
| Rhizopus arrhizus | Rhizopus arrhizus |  |
| Saccharomyces cerevisiae | Saccharomyces cerevisiae |  |
| 4 |  |  |
| Cladosporium sphaerospermum | Cladosporium sphaerospermum |  |
| Penicillium chrysogenum | Penicillium sect. Chrysogena (series Chrysogena) | P. chrysogenum + P. rubens |
| Aspergillus fumigatus | Aspergillus fumigatus |  |
| Chaetomium globosum | Chaetomium globosum |  |
| Memnoniella echinata | Memnoniella echinata |  |
| 5 |  |  |
| Cladosporium cladosporioides | Cladosporium cladosporioides species complex | C. tenuissimum + inversicolor + cladosporioides complex |
| Aspergillus chevalieri | Aspergillus sect. Aspergillus (series Chevalierorum) | A. montevidensis + chevalieri |
| Cephalotrichum stemonitis | Cephalotrichum stemonitis species complex | C. stemonitis + C. nanum |
| Scedosporium apiospermum | Scedosporium apiospermum |  |
| Syncephalastrum racemosum | NID |  |
| 6 |  |  |
| Torula herbarum | NID | not in database |
| Aspergillus subalbidus | Aspergillus subalbidus |  |
| Saccharomyces cerevisiae | Saccharomyces cerevisiae |  |
| Exophiala jeanselmei | Exophiala jeanselmei |  |
| Sporobolomyces roseus | Sporobolomyces roseus |  |
| 7 |  |  |
| Cladosporium herbarum | Cladosporium herbarum species complex | C. sinuosum + allicinum + herbarum |
| Paecilomyces variotii | Paecilomyces variotii |  |
| Scopulariopsis brevicaulis | Scopulariopsis brevicaulis |  |
| Apiospora montagnei (= Arthrinium arundinis) | Arthrinium arundinis |  |
| Exophiala jeanselmei | Exophiala jeanselmei |  |
| 8 |  |  |
| Cladosporium sphaerospermum | Cladosporium sphaerospermum |  |
| Aspergillus floridensis | Aspergillus sect. Nigri (series Japonici) | A. japonicus + uvarum |
| Beauveria bassiana | Beauveria bassiana |  |
| Cephalotrichum stemonitis | Cephalotrichum stemonitis species complex | C. stemonitis + nanum |
| Syncephalastrum racemosum | Fungi sp. | Syncephalastrum monosporum (83,186) |
| 9 |  |  |
| Cladosporium cladosporioides | Cladosporium cladosporioides species complex | C. phyllophilum + inversicolor + pseudocladosporioides |
| Aspergillus restrictus | Aspergillus restrictus |  |
| Rhizopus arrhizus | Rhizopus arrhizus |  |
| Exophiala jeanselmei | Exophiala jeanselmei |  |
| Sporothrix schenckii | Sporothrix schenckii |  |
| 10 |  |  |
| Cladosporium sphaerospermum | Cladosporium sphaerospermum |  |
| Purpureocillium lilacinum | Purpureocillium sp. | P. lilacinum + P. sp. |
| Rhodotorula mucilaginosa | Rhodotorula mucilaginosa |  |
| Alternaria alternata | Alternaria sect. Alternata | A. alternata + arborescens |
| Exophiala jeanselmei | Exophiala jeanselmei |  |
| 11 |  |  |
| Cladosporium sphaerospermum | Cladosporium sphaerospermum |  |
| Aspergillus restrictus | Aspergillus restrictus |  |
| Cephalotrichum stemonitis | Cephalotrichum stemonitis species complex | C. stemonitis + nanum |
| Lichtheimia corymbifera | Lichtheimia corymbifera |  |
| Sporothrix schenckii | Sporothrix schenckii |  |
| 12 |  |  |
| Cladosporium herbarum | Cladosporium herbarum species complex | C. allicinum + sinuosum + herbarum |
| Aspergillus ustus | Aspergillus ustus |  |
| Penicillium brevicompactum | Penicillium brevicompactum |  |
| Apiospora montagnei (= Arthrinium arundinis) | Arthrinium arundinis |  |
| Sporobolomyces roseus | Sporobolomyces roseus |  |
| 13 |  |  |
| Sarocladium strictum | Sarocladium strictum |  |
| Aspergillus ochraceus | Aspergillus ochraceus |  |
| Penicillium brevicompactum | Penicillium brevicompactum |  |
| Memnoniella echinata | Memnoniella echinata |  |
| Cryptococcus neoformans | Cryptococcus neoformans | C. neoformans + var. neoformans + var. grubii |
| 14 |  |  |
| Cladosporium herbarum | Cladosporium herbarum species complex | C. allicinum + sinuosum + herbarum |
| Fusarium culmorum | Fusarium sambucinum species complex | Fusarium cerealis + culmorum + sambucinum species complex |
| Penicillium digitatum | Penicillium digitatum |  |
| Cephalotrichum stemonitis | Cephalotrichum stemonitis species complex | C. stemonitis + nanum |
| Rhodotorula mucilaginosa | Rhodotorula mucilaginosa |  |
| 15 |  |  |
| Cladosporium herbarum | Cladosporium herbarum species complex | C. allicinum + sinuosum + herbarum |
| Purpureocillium lilacinum | Purpureocillium sp. |  |
| Penicillium digitatum | Penicillium digitatum |  |
| Aureobasidium pullulans | Aureobasidium sp. | A. pullulans + sp. |
| Rhodotorula mucilaginosa | Rhodotorula mucilaginosa |  |
| 16 |  |  |
| Cladosporium cladosporioides | Cladosporium cladosporioides species complex | C. tenuissimum + inversicolor + cladosporioides complex |
| Fusarium culmorum | Fusarium sambucinum species complex | Fusarium cerealis + culmorum + sambucinum species complex |
| Penicillium glabrum | Penicillium sect. Aspergilloides (series Glabra) | P. sect. Aspergilloides (glabrum-clade) + glabrum |
| Stachybotrys chartarum | Stachybotrys chartarum species complex | S. chlorohalonata + chartarum |
| Geotrichum candidum | Geotrichum candidum |  |
| 17 |  |  |
| Cladosporium cladosporioides | Cladosporium cladosporioides species complex | C. tenuissimum + inversicolor + cladosporioides complex |
| Paecilomyces variotii | Paecilomyces variotti |  |
| Penicillium glabrum | Penicillium sect. Aspergilloides (series Glabra) | P. sect. Aspergilloides (glabrum-clade) + glabrum |
| Epicoccum nigrum | Epicoccum nigrum |  |
| Rhizopus arrhizus | Rhizopus arrhizus |  |
| 18 |  |  |
| Penicillium citreonigrum | Penicillium sect. Exilicaulis (series Citreonigra) | P. citreonigrum + citreosulfuratum |
| Paecilomyces variotii | Paecilomyces variotti |  |
| Penicillium corylophilum | Penicillium sect. Exilicaulis (series Corylophila) | P. corylophilum + rubefaciens |
| Epicoccum nigrum | Epicoccum nigrum |  |
| Cryptococcus neoformans | Cryptococcus neoformans |  |
| 19 |  |  |
| Gliomastix murorum | Gliomastix murorum |  |
| Fusarium culmorum | Fusarium sambucinum species complex | Fusarium cerealis + culmorum + sambucinum species complex |
| Penicillium corylophilum | Penicillium corylophilum |  |
| Penicillium chrysogenum | Penicillium sect. Chrysogena (series Chrysogena) | P. chrysogenum + rubens |
| Lichtheimia corymbifera | Lichtheimia corymbifera |  |
| 20 |  |  |
| Cladosporium cladosporioides | Cladosporium cladosporioides species complex | C. tenuissimum + inversicolor + cladosporioides complex |
| Purpureocillium lilacinum | Purpureocillium sp. | Purpureocillium lilacinum + sp. |
| Penicillium chrysogenum | Penicillium sect. Chrysogena (series Chrysogena) | P. chrysogenum + rubens |
| Phoma herbarum | Phoma herbarum |  |
| Saccharomyces cerevisiae | Saccharomyces cerevisiae |  |
| 21 |  |  |
| Alternaria alternata | Alternaria sect. Alternata | A. alternata + arborescens |
| Aspergillus fumigatus | Aspergillus fumigatus |  |
| Penicillium halotolerans | Penicillium halotolerans |  |
| Trichothecium roseum | Trichothecium roseum |  |
| Tritirachium oryzae | Tritirachium oryzae |  |
| 22 |  |  |
| Cryptococcus neoformans | Cryptococcus neoformans |  |
| Aspergillus versicolor | Aspergillus sect. Nidulantes (series Versicolores) | A. jensenii + creber + versicolor-clade |
| Penicillium digitatum | Penicillium digitatum |  |
| Scopulariopsis brevicaulis | Scopulariopsis brevicaulis |  |
| Rhizopus arrhizus | Rhizopus arrhizus |  |
| 23 |  |  |
| Rhodotorula mucilaginosa | Rhodotorula mucilaginosa |  |
| Aspergillus fumigatus | Aspergillus fumigatus |  |
| Penicillium digitatum | Penicillium digitatum |  |
| Stachybotrys chartarum | Stachybotrys chartarum species complex | S. chlorohalonata + chartarum |
| Scopulariopsis brevicaulis | Scopulariopsis brevicaulis |  |
| 24 |  |  |
| Cladosporium sphaerospermum | Cladosporium sphaerospermum |  |
| Sarocladium strictum | Sarocladium strictum |  |
| Penicillium brevicompactum | Penicillium brevicompactum |  |
| Stachybotrys chartarum | Stachybotrys chartarum species complex | S. chlorohalonata + chartarum |
| Lichtheimia corymbifera | Lichtheimia corymbifera |  |
| 25 |  |  |
| Cladosporium sphaerospermum | Cladosporium sphaerospermum |  |
| Cryptococcus neoformans | Cryptococcus neoformans |  |
| Penicillium brevicompactum | Penicillium brevicompactum |  |
| Stachybotrys chartarum | Stachybotrys chartarum species complex | S. chlorohalonata + chartarum |
| Lichtheimia corymbifera | Lichtheimia corymbifera |  |
| 26 |  |  |
| Cladosporium sphaerospermum | Cladosporium sphaerospermum |  |
| Aspergillus ustus | Aspergillus ustus |  |
| Penicillium brevicompactum | Penicillium brevicompactum |  |
| Phoma herbarum | Phoma herbarum |  |
| Cryptococcus neoformans | Cryptococcus neoformans |  |
| 27 |  |  |
| Syncephalastrum racemosum | NID |  |
| Aspergillus versicolor | Aspergillus sect. Nidulantes (series Versicolores) | A. jensenii + creber + versicolor-clade |
| Penicillium chrysogenum | Penicillium sect. Chrysogena (series Chrysogena) | P. chrysogenum + rubens |
| Tritirachium oryzae | Tritirachium oryzae |  |
| Apiospora montagnei (= Arthrinium arundinis) | Arthrinium arundinis |  |
| 28 |  |  |
| Torula herbarum | NID | not in database |
| Aspergillus versicolor | Aspergillus sect. Nidulantes (series Versicolores) | A. jensenii + creber + versicolor-clade |
| Penicillium halotolerans | Penicillium sect. Chrysogena | P. dipodomyis + nalgiovense + kewense + halotolerans |
| Cunninghamella echinulata | Cunninghamella echinulata |  |
| Trichothecium roseum | Trichothecium roseum |  |
| 29 |  |  |
| Rhodotorula mucilaginosa | Rhodotorula mucilaginosa |  |
| Aspergillus fumigatus | Aspergillus fumigatus |  |
| Penicillium chrysogenum | Penicillium sect. Chrysogena (series Chrysogena) | P. chrysogenum + rubens |
| Trichothecium roseum | Trichothecium roseum |  |
| Thamnidium elegans | Thamnidium elegans |  |
| 30 |  |  |
| Sporothrix schenckii | Sporothrix schenckii |  |
| Tritirachium oryzae | Tritirachium oryzae |  |
| Penicillium chrysogenum | Penicillium sect. Chrysogena (series Chrysogena) | P. chrysogenum + rubens |
| Cunninghamella echinulata | Cunninghamella echinulata |  |
| Beauveria bassiana | Beauveria bassiana |  |
| 31 |  |  |
| Tritirachium oryzae | Tritirachium oryzae |  |
| Aspergillus fumigatus | Aspergillus fumigatus |  |
| Penicillium aurantiogriseum | Penicillium sect. Fasciculata (series Viridicata) | P. freii + aurantiogriseum |
| Trichothecium roseum | Trichothecium roseum |  |
| Purpureocillium lilacinum | Purpureocillium sp. | P. lilacinum + sp. |
| 32 |  |  |
| Cladosporium herbarum | Cladosporium herbarum species complex | C. sinuosum + allicinum + herbarum |
| Thamnidium elegans | Thamnidium elegans |  |
| Penicillium aurantiogriseum | Penicillium sect. Fasciculata (series Viridicata) | P. freii + aurantiogriseum |
| Memnoniella echinata | Memnoniella echinata |  |
| Sporobolomyces roseus | Sporobolomyces roseus |  |
| 33 |  |  |
| Cladosporium cladosporioides | Cladosporium cladosporioides species complex | C. tenuissimum + inversicolor + cladosporioides species complex |
| Aspergillus chevalieri | Aspergillus sect. Aspergillus (series Chevalierorum) | A. montevidensis + chevalieri |
| Penicillium aurantiogriseum | Penicillium sect. Fasciculata (series Viridicata) | P. freii + aurantiogriseum |
| Alternaria alternata | Alternaria sect. Alternata | A. alternata + arborescens |
| Geotrichum candidum | Geotrichum candidum |  |
| 34 |  |  |
| Cladosporium cladosporioides | Cladosporium cladosporioides species complex | C. tenuissimum + inversicolor + cladosporioides species complex |
| Penicillium citreonigrum | Penicillium sect. Exilicaulis (series Citreonigra) | P. citreonigrum + citreosulfuratum |
| Penicillium chrysogenum | Penicillium sect. Chrysogena (series Chrysogena) | P. chrysogenum + rubens |
| Sarocladium strictum | Sarocladium strictum |  |
| Cunninghamella echinulata | Cunninghamella echinulata |  |
| 35 |  |  |
| Cladosporium herbarum | Cladosporium herbarum species complex | C. sinuosum + allicinum + herbarum |
| Fusarium culmorum | Fusarium sambucinum species complex | Fusarium cerealis + culmorum + sambucinum species complex |
| Penicillium aurantiogriseum | Penicillium sect. Fasciculata (series Viridicata) | P. freii + P. aurantiogriseum |
| Sarocladium strictum | Sarocladium strictum |  |
| Alternaria alternata | Alternaria sect. Alternata | A. alternata + arborescens |
| 36 |  |  |
| Penicillium halotolerans | Penicillium sect. Chrysogena | P. halotolerans + kewense + nalgiovense + dipodomyis |
| Aspergillus ustus | Aspergillus ustus |  |
| Cladosporium cladosporioides | Cladosporium cladosporioides species complex | C. tenuissimum + inversicolor + cladosporioides species complex |
| Stachybotrys chartarum | Stachybotrys chartarum species complex | S. chlorohalonata + chartarum |
| Saccharomyces cerevisiae | Saccharomyces cerevisiae |  |
| 37 |  |  |
| Penicillium halotolerans | Penicillium sect. Chrysogena | P. dipodomyis + nalgiovense + lanosocoeruleum + chrysogenum + halotolerans |
| Aspergillus ustus | Aspergillus ustus |  |
| Cladosporium cladosporioides | Cladosporium cladosporioides species complex | C. tenuissimum + inversicolor + cladosporioides species complex |
| Stachybotrys chartarum | Stachybotrys chartarum species complex | S. chlorohalonata + chartarum |
| Saccharomyces cerevisiae | Saccharomyces cerevisiae |  |

Table 17: comparison of the ITS2 Blast results of the IHEM database to the original composition of the DMCs. The blast results in which multiple species were retrieved with equal e-value and percentage, are manually classified to a subgeneric level (section, series or species complex). NID: no identification.

| **Original species composition** | **subgeneric classification** | **top hits** |
| --- | --- | --- |
| 1 |  |  |
| *Aspergillus versicolor* | Aspergillus sect. Nidulantes (series Versicolores) | A. jensenii + A. creber + versicolor-clade |
| *Aspergillus fumigatus* | Aspergillus fumigatus |  |
| *Cladosporium sphaerospermum* | Cladosporium sphaerospermum |  |
| *Stachybotrys chartarum* | Stachybotrys chartarum |  |
| *Penicillium chrysogenum* | Penicillium sect. Chrysogena | P. dipodomyis + nalgiovense + lanosocoeruleum + chrysogenum + halotolerans |
| 2 |  |  |
| Aspergillus versicolor | Aspergillus sect. Nidulantes (series Versicolores) | A. puulaauensis + A. creber |
| Stachybotrys chartarum | Stachybotrys chartarum |  |
| Aureobasidium pullulans | Aureobasidium pullulans |  |
| Cladosporium herbarum | Cladosporium herbarum species complex | C. allicinum + sinuosum + ramotenellum |
| Penicillium aurantiogriseum | Penicillium sect. Fasciculata (series Viridicata) | P. freii + aurantiogriseum |
| 3 |  |  |
| Cladosporium cladosporioides | Cladosporium cladosporioides species complex | C. phyllophilum + inversicolor + pseudocladosporioides |
| Aspergillus ochraceus | Aspergillus sect. Circumdati (series Circumdati) | A. ochraceus + A. melleus |
| Chaetomium globosum | Chaetomium globosum |  |
| Rhizopus arrhizus | Rhizopus arrhizus |  |
| Saccharomyces cerevisiae | Saccharomyces cerevisiae |  |
| 4 |  |  |
| Cladosporium sphaerospermum | Cladosporium sphaerospermum |  |
| Penicillium chrysogenum | Penicillium sect. Chrysogena | P. dipodomyis + nalgiovense + lanosocoeruleum + chrysogenum + halotolerans |
| Aspergillus fumigatus | Aspergillus fumigatus |  |
| Chaetomium globosum | Chaetomium globosum |  |
| Memnoniella echinata | Memnoniella echinata |  |
| 5 |  |  |
| Cladosporium cladosporioides | Cladosporium cladosporioides species complex | C. phyllophilum + inversicolor + pseudocladosporioides |
| Aspergillus chevalieri | Aspergillus sect. Aspergillus (series Chevalierorum) | A. montevidensis + chevalieri |
| Cephalotrichum stemonitis | Cephalotrichum stemonitis species complex | C. stemonitis + C. nanum |
| Scedosporium apiospermum | Scedosporium apiospermum |  |
| Syncephalastrum racemosum | NID |  |
| 6 |  |  |
| Torula herbarum | NID | not in database |
| Aspergillus subalbidus | Aspergillus sect. Candidi | A. subalbidus + candidus |
| Saccharomyces cerevisiae | Saccharomyces cerevisiae |  |
| Exophiala jeanselmei | Exophiala jeanselmei |  |
| Sporobolomyces roseus | Sporobolomyces roseus |  |
| 7 |  |  |
| Cladosporium herbarum | Cladosporium herbarum species complex | C. allicinum + sinuosum + ramotenellum |
| Paecilomyces variotii | Paecilomyces variotii |  |
| Scopulariopsis brevicaulis | Scopulariopsis brevicaulis |  |
| Apiospora montagnei (= Arthrinium arundinis) | Arthrinium arundinis |  |
| Exophiala jeanselmei | Exophiala jeanselmei |  |
| 8 |  |  |
| Cladosporium sphaerospermum | Cladosporium sphaerospermum |  |
| Aspergillus floridensis | Aspergillus sect. Nigri (series Japonici) | A. brunneoviolaceus + aculeatinus |
| Beauveria bassiana | Beauveria bassiana |  |
| Cephalotrichum stemonitis | Cephalotrichum stemonitis species complex | C. stemonitis + nanum |
| Syncephalastrum racemosum | NID |  |
| 9 |  |  |
| Cladosporium cladosporioides | Cladosporium cladosporioides species complex | C. phyllophilum + inversicolor + pseudocladosporioides |
| Aspergillus restrictus | Aspergillus restrictus |  |
| Rhizopus arrhizus | Rhizopus arrhizus |  |
| Exophiala jeanselmei | Exophiala jeanselmei |  |
| Sporothrix schenckii | Sporothrix schenckii |  |
| 10 |  |  |
| Cladosporium sphaerospermum | Cladosporium sphaerospermum |  |
| Purpureocillium lilacinum | Purpureocillium lilacinum |  |
| Rhodotorula mucilaginosa | Rhodotorula mucilaginosa |  |
| Alternaria alternata | Alternaria alternata |  |
| Exophiala jeanselmei | Exophiala jeanselmei |  |
| 11 |  |  |
| Cladosporium sphaerospermum | Cladosporium sphaerospermum |  |
| Aspergillus restrictus | Aspergillus restrictus |  |
| Cephalotrichum stemonitis | Cephalotrichum stemonitis species complex | C. stemonitis + nanum |
| Lichtheimia corymbifera | Lichtheimia corymbifera |  |
| Sporothrix schenckii | Sporothrix schenckii |  |
| 12 |  |  |
| Cladosporium herbarum | Cladosporium herbarum species complex | C. allicinum + sinuosum + herbarum |
| Aspergillus ustus | Aspergillus sect. Usti (series Usti) | A. ustus + pseudoustus + puniceus |
| Penicillium brevicompactum | Penicillium brevicompactum |  |
| Apiospora montagnei (= Arthrinium arundinis) | Arthrinium arundinis |  |
| Sporobolomyces roseus | Sporobolomyces roseus |  |
| 13 |  |  |
| Sarocladium strictum | Sarocladium strictum |  |
| Aspergillus ochraceus | A. sect. Circumdati (series Circumdati) | A. ochraceus + melleus |
| Penicillium brevicompactum | Penicillium brevicompactum |  |
| Memnoniella echinata | Memnoniella echinata |  |
| Cryptococcus neoformans | C. neoformans/C. gattii species complex | C. gattii + neoformans |
|  |  |  |
| 14 |  |  |
| Cladosporium herbarum | Cladosporium herbarum species complex | C. allicinum + sinuosum + ramotenellum |
| Fusarium culmorum | Fusarium sambucinum species complex | Fusarium cerealis + culmorum + sambucinum species complex |
| Penicillium digitatum | Penicillium digitatum |  |
| Cephalotrichum stemonitis | Cephalotrichum stemonitis species complex | C. stemonitis + nanum |
| Rhodotorula mucilaginosa | Rhodotorula mucilaginosa |  |
| 15 |  |  |
| Cladosporium herbarum | Cladosporium herbarum species complex | C. allicinum + sinuosum + ramotenellum |
| Purpureocillium lilacinum | Purpureocillium lilacinum |  |
| Penicillium digitatum | Penicillium digitatum |  |
| Aureobasidium pullulans | Aureobasidium sp. | A. pullulans + sp. |
| Rhodotorula mucilaginosa | Rhodotorula mucilaginosa |  |
| 16 |  |  |
| Cladosporium cladosporioides | Cladosporium cladosporioides species complex | Cladosporium cladosporioides species complex |
| Fusarium culmorum | Fusarium sambucinum species complex | Fusarium cerealis + culmorum + sambucinum species complex |
| Penicillium glabrum | Penicillium sect. Aspergilloides (series Glabra) | P. sect. Aspergilloides (glabrum-clade) + glabrum |
| Stachybotrys chartarum | Stachybotrys chartarum |  |
| Geotrichum candidum | Geotrichum candidum |  |
| 17 |  |  |
| Cladosporium cladosporioides | Cladosporium cladosporioides species complex | C. phyllophilum + inversicolor + pseudocladosporioides |
| Paecilomyces variotii | Paecilomyces variotti |  |
| Penicillium glabrum | Penicillium sect. Aspergilloides (series Glabra) | P. sect. Aspergilloides (glabrum-clade) + glabrum |
| Epicoccum nigrum | Epicoccum nigrum |  |
| Rhizopus arrhizus | Rhizopus arrhizus |  |
| 18 |  |  |
| Penicillium citreonigrum | Penicillium citreonigrum |  |
| Paecilomyces variotii | Paecilomyces variotti |  |
| Penicillium corylophilum | Penicillium sect. Exilicaulis (series Corylophila) | P. corylophilum + rubefaciens |
| Epicoccum nigrum | Epicoccum nigrum |  |
| Cryptococcus neoformans | C. neoformans/C. gattii species complex | C. gattii + neoformans |
| 19 |  |  |
| Gliomastix murorum | Gliomastix murorum |  |
| Fusarium culmorum | Fusarium sambucinum species complex | Fusarium cerealis + culmorum + sambucinum species complex |
| Penicillium corylophilum | Penicillium corylophilum |  |
| Penicillium chrysogenum | Penicillium sect. Chrysogena | P. dipodomyis + nalgiovense + lanosocoeruleum + chrysogenum + halotolerans |
| Lichtheimia corymbifera | Lichtheimia corymbifera |  |
|  |  |  |
| 20 |  |  |
| Cladosporium cladosporioides | Cladosporium cladosporioides species complex | C. phyllophilum + pseudocladosporioides + inversicolor |
| Purpureocillium lilacinum | Purpureocillium lilacinum |  |
| Penicillium chrysogenum | Penicillium sect. Chrysogena | P. dipodomyis + nalgiovense + lanosocoeruleum + chrysogenum + halotolerans |
| Phoma herbarum | Phoma herbarum |  |
| Saccharomyces cerevisiae | Saccharomyces cerevisiae |  |
| 21 |  |  |
| Alternaria alternata | Alternaria alternata |  |
| Aspergillus fumigatus | Aspergillus fumigatus |  |
| Penicillium halotolerans | Penicillium sect. Chrysogena | P. dipodomyis + nalgiovense + lanosocoeruleum + chrysogenum + halotolerans |
| Trichothecium roseum | Trichothecium roseum |  |
| Tritirachium oryzae | Tritirachium oryzae |  |
| 22 |  |  |
| Cryptococcus neoformans | C. neoformans/C. gattii species complex | C. gattii + neoformans |
| Aspergillus versicolor | Aspergillus sect. Nidulantes (series Versicolores) | A. creber + puulaauensis |
| Penicillium digitatum | Penicillium digitatum |  |
| Scopulariopsis brevicaulis | Scopulariopsis brevicaulis |  |
| Rhizopus arrhizus | Rhizopus arrhizus |  |
| 23 |  |  |
| Rhodotorula mucilaginosa | Rhodotorula mucilaginosa |  |
| Aspergillus fumigatus | Aspergillus fumigatus |  |
| Penicillium digitatum | Penicillium digitatum |  |
| Stachybotrys chartarum | Stachybotrus chartarum |  |
| Scopulariopsis brevicaulis | Scopulariopsis brevicaulis |  |
| 24 |  |  |
| Cladosporium sphaerospermum | Cladosporium sphaerospermum |  |
| Sarocladium strictum | Sarocladium strictum |  |
| Penicillium brevicompactum | Penicillium brevicompactum |  |
| Stachybotrys chartarum | Stachybotrys chartarum |  |
| Lichtheimia corymbifera | Lichtheimia corymbifera |  |
| 25 |  |  |
| Cladosporium sphaerospermum | Cladosporium sphaerospermum |  |
| Cryptococcus neoformans | C. neoformans/C. gattii species complex | C. gattii + neoformans |
| Penicillium brevicompactum | Penicillium brevicompactum |  |
| Stachybotrys chartarum | Stachybotrys chartarum |  |
| Lichtheimia corymbifera | Lichtheimia corymbifera |  |
| 26 |  |  |
| Cladosporium sphaerospermum | Cladosporium sphaerospermum |  |
| Aspergillus ustus | Aspergillus sect. Usti (series Usti) | A. ustus + pseudoustus + puniceus |
| Penicillium brevicompactum | Penicillium brevicompactum |  |
| Phoma herbarum | Phoma herbarum |  |
| Cryptococcus neoformans | C. neoformans/C. gattii species complex | C. gattii + neoformans |
| 27 |  |  |
| Syncephalastrum racemosum | NID |  |
| Aspergillus versicolor | Aspergillus sect. Nidulantes (series Versicolores) | A. jensenii + creber + versicolor-clade |
| Penicillium chrysogenum | Penicillium sect. Chrysogena | P. dipodomyis + nalgiovense + lanosocoeruleum + chrysogenum + halotolerans |
| Tritirachium oryzae | Tritirachium oryzae |  |
| Apiospora montagnei (= Arthrinium arundinis) | Arthrinium arundinis |  |
| 28 |  |  |
| Torula herbarum | NID | not in database |
| Aspergillus versicolor | Aspergillus sect. Nidulantes (series Versicolores) | A. creber + puulaauensis |
| Penicillium halotolerans | Penicillium sect. Chrysogena | P. dipodomyis + nalgiovense + lanosocoeruleum + chrysogenum + halotolerans |
| Cunninghamella echinulata | Cunninghamella echinulata |  |
| Trichothecium roseum | Trichothecium roseum |  |
| 29 |  |  |
| Rhodotorula mucilaginosa | Rhodotorula mucilaginosa |  |
| Aspergillus fumigatus | Aspergillus fumigatus |  |
| Penicillium chrysogenum | Penicillium sect. Chrysogena | P. dipodomyis + nalgiovense + lanosocoeruleum + chrysogenum + halotolerans |
| Trichothecium roseum | Trichothecium roseum |  |
| Thamnidium4 elegans | Thamnidium elegans |  |
| 30 |  |  |
| Sporothrix schenckii | Sporothrix schenckii |  |
| Tritirachium oryzae | Tritirachium oryzae |  |
| Penicillium chrysogenum | Penicillium sect. Chrysogena | P. dipodomyis + nalgiovense + lanosocoeruleum + chrysogenum + halotolerans |
| Cunninghamella echinulata | Cunninghamella echinulata |  |
| Beauveria bassiana | Beauveria bassiana |  |
| 31 |  |  |
| Tritirachium oryzae | Tritirachium oryzae |  |
| Aspergillus fumigatus | Aspergillus fumigatus |  |
| Penicillium aurantiogriseum | Penicillium sect. Fasciculata (series Viridicata) | P. freii + aurantiogriseum |
| Trichothecium roseum | Trichothecium roseum |  |
| Purpureocillium lilacinum | Purpureocillium lilacinum |  |
| 32 |  |  |
| Cladosporium herbarum | Cladosporium herbarum species complex |  |
| Thamnidium elegans | Thamnidium elegans |  |
| Penicillium aurantiogriseum | Penicillium sect. Fasciculata (series Viridicata) | P. freii + aurantiogriseum |
| Memnoniella echinata | Memnoniella echinata |  |
| Sporobolomyces roseus | Sporobolomyces roseus |  |
| 33 |  |  |
| Cladosporium cladosporioides | Cladosporium cladosporioides species complex | C. phyllophilum + inversicolor + pseudocladosporioides |
| Aspergillus chevalieri | Aspergillus sect. Aspergillus (series Chevalierorum) | A. montevidensis + chevalieri |
| Penicillium aurantiogriseum | Penicillium sect. Fasciculata (series Viridicata) | P. freii + P. aurantiogriseum |
| Alternaria alternata | Alternaria alternata |  |
| Geotrichum candidum | Geotrichum candidum |  |
| 34 |  |  |
| Cladosporium cladosporioides | Cladosporium cladosporioides species complex | C. phyllophilum + pseudocladosporioides + inversicolor |
| Penicillium citreonigrum | Penicillium citreonigrum |  |
| Penicillium chrysogenum | Penicillium sect. Chrysogena | P. dipodomyis + nalgiovense + lanosocoeruleum + chrysogenum + halotolerans |
| Sarocladium strictum | Sarocladium strictum |  |
| Cunninghamella echinulata | Cunninghamella echinulata |  |
| 35 |  |  |
| Cladosporium herbarum | Cladosporium herbarum species complex | C. ramotenellum + allicinum + sinuosum |
| Fusarium culmorum | Fusarium sambucinum species complex | Fusarium cerealis + culmorum + sambucinum species complex |
| Penicillium aurantiogriseum | Penicillium sect. Fasciculata (series Viridicata) | P. freii + P. aurantiogriseum |
| Sarocladium strictum | Sarocladium strictum |  |
| Alternaria alternata | Alternaria alternata |  |
| 36 |  |  |
| Penicillium halotolerans | Penicillium sect. Chrysogena | P. dipodomyis + nalgiovense + lanosocoeruleum + chrysogenum + halotolerans |
| Aspergillus ustus | Aspergillus sect. Usti (series Usti) | A. ustus + pseudoustus + puniceus |
| Cladosporium cladosporioides | Cladosporium cladosporioides species complex | C. tenuissimum + inversicolor + cladosporioides species complex |
| Stachybotrys chartarum | Stachybotrys chartarum |  |
| Saccharomyces cerevisiae | Saccharomyces cerevisiae |  |
| 37 |  |  |
| Penicillium halotolerans | Penicillium sect. Chrysogena | P. dipodomyis + nalgiovense + lanosocoeruleum + chrysogenum + halotolerans |
| Aspergillus ustus | Aspergillus sect. Usti (series Usti) | A. ustus + pseudoustus + puniceus |
| Cladosporium cladosporioides | Cladosporium cladosporioides species complex | C. phyllophilum + pseudocladosporioides + inversicolor |
| Stachybotrys chartarum | Stachybotrys chartarum |  |
| Saccharomyces cerevisiae | Saccharomyces cerevisiae |  |

Table 18: Read abundance deviation of correctly identified species using Illumina sequences and ITS1 at the species level. For each combination of database, software and method four numbers are given: the first number indicates the total number of DMCs the species is present in; the second number indicates the number of times the abundance was correct within a 5% margin; the third and fourth numbers indicate the number of times the abundance was overestimated or underestimated by at least 5%, respectively.

|  | **IHEM** | | | | **UNITE** | | | |
| --- | --- | --- | --- | --- | --- | --- | --- | --- |
|  | **BLAST** | | **MOTHUR** | | **BLAST** | | **MOTHUR** | |
| **Species** | **Strict** | **Loose** | **Wang** | **knn** | **Strict** | **Loose** | **Wang** | **knn** |
|  |  |  |  |  |  |  |  |  |
| *Alternaria alternata* | 4-0-0-0 | 4-3-0-1 | 4-0-0-0 | 4-0-0-0 | 4-0-0-0 | 4-0-0-0 | 4-0-0-0 | 4-0-0-0 |
| *Apiospora montagnei* | 3-0-0-3 | 3-0-0-3 | 3-0-0-3 | 3-0-0-3 | 3-0-0-0 | 3-0-0-0 | 3-0-0-0 | 3-0-0-0 |
| *Aspergillus chevalieri* | 2-0-0-0 | 2-1-0-1 | 2-0-0-0 | 2-0-0-0 | 2-0-0-0 | 2-0-0-0 | 2-0-0-0 | 2-0-0-0 |
| *Aspergillus floridensis* | 1-0-0-0 | 1-0-0-0 | 1-0-0-0 | 1-0-0-0 | 1-0-0-0 | 1-0-0-0 | 1-0-0-0 | 1-0-0-0 |
| *Aspergillus fumigatus* | 6-1-5-0 | 6-2-4-0 | 6-1-5-0 | 6-1-5-0 | 6-0-0-0 | 6-0-0-0 | 6-0-0-0 | 6-0-0-0 |
| *Aspergillus ochraceus* | 2-0-2-0 | 2-0-2-0 | 2-0-0-0 | 2-0-0-0 | 2-0-2-0 | 2-0-2-0 | 2-0-0-0 | 2-0-0-0 |
| *Aspergillus restrictus* | 2-0-2-0 | 2-1-1-0 | 2-1-1-0 | 2-1-1-0 | 2-0-2-0 | 2-0-2-0 | 2-0-2-0 | 2-0-2-0 |
| *Aspergillus subalbidus* | 1-1-0-0 | 1-1-0-0 | 1-0-1-0 | 1-0-1-0 | 1-0-0-0 | 1-0-0-0 | 1-0-0-0 | 1-0-0-0 |
| *Aspergillus ustus* | 4-0-4-0 | 4-3-1-0 | 4-0-0-0 | 4-0-0-0 | 4-2-2-0 | 4-2-2-0 | 4-0-0-0 | 4-0-0-0 |
| *Aspergillus versicolor* | 5-0-0-0 | 5-2-2-1 | 5-0-0-0 | 5-0-0-0 | 5-0-0-0 | 5-0-0-0 | 5-0-0-0 | 5-0-0-0 |
| *Aureobasidium pullulans* | 2-0-0-0 | 2-1-1-0 | 2-0-0-0 | 2-0-0-0 | 2-0-0-1 | 2-0-0-1 | 2-0-0-0 | 2-0-0-0 |
| *Beauveria bassiana* | 2-0-2-0 | 2-1-1-0 | 2-1-1-0 | 2-1-1-0 | 2-0-2-0 | 2-1-1-0 | 2-0-0-0 | 2-0-0-0 |
| *Cephalotrichum stemonitis* | 4-0-0-0 | 4-0-0-4 | 4-0-0-0 | 4-0-0-0 | 4-0-0-0 | 4-0-0-0 | 4-0-0-0 | 4-0-0-0 |
| *Chaetomium globosum* | 2-2-0-0 | 2-1-0-1 | 2-1-1-0 | 2-1-1-0 | 2-0-0-0 | 2-1-0-1 | 2-0-0-0 | 2-0-0-0 |
| *Cladosporium cladosporioides* | 10-0-0-0 | 10-1-9-0 | 10-0-0-0 | 10-0-0-0 | 10-0-0-0 | 10-0-0-0 | 10-0-0-0 | 10-0-0-0 |
| *Cladosporium herbarum* | 7-0-0-0 | 7-4-3-0 | 7-2-5-0 | 7-2-5-0 | 7-0-0-0 | 7-0-0-0 | 7-0-0-0 | 7-0-0-0 |
| *Cladosporium sphaerospermum* | 8-2-6-0 | 8-3-5-0 | 8-1-7-0 | 8-1-7-0 | 8-2-6-0 | 8-2-6-0 | 8-0-8-0 | 8-0-8-0 |
| *Cryptococcus neoformans* var. grubii | 5-1-4-0 | 5-3-2-0 | 5-2-3-0 | 5-2-3-0 | 5-1-4-0 | 5-2-3-0 | 5-0-5-0 | 5-0-5-0 |
| *Cunninghamella echinulata* | 3-0-0-3 | 3-0-0-3 | 3-0-0-3 | 3-0-0-3 | 3-0-0-0 | 3-0-0-0 | 3-0-0-3 | 3-0-0-3 |
| *Epicoccum nigrum* | 2-0-2-0 | 2-1-0-1 | 2-2-0-0 | 2-2-0-0 | 2-0-0-0 | 2-1-0-1 | 2-0-0-0 | 2-0-0-0 |
| *Exophiala jeanselmei* | 4-2-0-2 | 4-0-0-4 | 4-0-0-4 | 4-0-0-4 | 4-0-0-0 | 4-0-0-0 | 4-0-0-0 | 4-0-0-0 |
| *Fusarium culmorum* | 4-0-0-0 | 4-0-1-3 | 4-1-1-2 | 4-1-1-2 | 4-0-0-3 | 4-0-0-3 | 4-0-0-0 | 4-0-0-0 |
| *Geotrichum candidum* | 2-0-2-0 | 2-2-0-0 | 2-0-2-0 | 2-0-2-0 | 2-0-0-0 | 2-0-0-0 | 2-0-0-0 | 2-0-0-0 |
| *Gliomastix murorum* | 1-0-1-0 | 1-0-0-1 | 1-0-0-1 | 1-0-0-1 | 1-0-0-1 | 1-0-0-1 | 1-0-0-1 | 1-0-0-1 |
| *Lichtheimia corymbifera* | 4-0-1-3 | 4-0-0-4 | 4-0-0-4 | 4-0-0-4 | 4-0-1-3 | 4-1-0-3 | 4-1-0-3 | 4-1-0-3 |
| *Memnoniella echinata* | 3-3-0-0 | 3-2-0-1 | 3-2-0-1 | 3-2-0-1 | 3-2-0-1 | 3-2-0-1 | 3-0-2-1 | 3-0-2-1 |
| *Paecilomyces variotii* | 3-0-3-0 | 3-2-0-1 | 3-3-0-0 | 3-3-0-0 | 3-1-2-0 | 3-1-1-1 | 3-2-1-0 | 3-2-1-0 |
| *Penicillium aurantiogriseum* | 5-0-0-0 | 5-4-0-1 | 5-0-0-0 | 5-0-0-0 | 5-4-1-0 | 5-3-1-1 | 5-4-1-0 | 5-4-1-0 |
| *Penicillium brevicompactum* | 5-2-0-3 | 5-2-0-3 | 5-4-0-1 | 5-4-0-1 | 5-2-0-3 | 5-2-0-3 | 5-0-0-0 | 5-0-0-0 |
| *Penicillium chrysogenum* | 8-0-0-0 | 8-0-0-8 | 8-0-0-0 | 8-0-0-0 | 8-0-0-8 | 8-0-0-8 | 8-0-0-0 | 8-0-0-0 |
| *Penicillium citreonigrum* | 2-0-0-0 | 2-0-1-1 | 2-0-1-1 | 2-0-1-1 | 2-0-0-0 | 2-0-1-1 | 2-0-0-0 | 2-0-0-0 |
| *Penicillium corylophilum* | 2-0-0-0 | 2-0-2-0 | 2-0-2-0 | 2-0-2-0 | 2-0-0-0 | 2-0-2-0 | 2-0-2-0 | 2-0-2-0 |
| *Penicillium digitatum* | 4-1-3-0 | 4-3-1-0 | 4-2-2-0 | 4-2-2-0 | 4-0-0-0 | 4-0-0-0 | 4-0-0-0 | 4-0-0-0 |
| *Penicillium glabrum* | 2-0-0-0 | 2-2-0-0 | 2-0-2-0 | 2-0-2-0 | 2-0-0-0 | 2-0-0-0 | 2-0-0-0 | 2-0-0-0 |
| *Penicillium halotolerans* | 4-0-4-0 | 4-2-2-0 | 4-0-0-0 | 4-0-0-0 | 4-0-0-0 | 4-0-0-0 | 4-0-0-0 | 4-0-0-0 |
| *Phoma herbarum* | 2-0-2-0 | 2-1-1-0 | 2-0-0-0 | 2-0-0-0 | 2-0-2-0 | 2-1-1-0 | 2-0-0-0 | 2-0-0-0 |
| *Purpureocillium lilacinum* | 4-0-0-0 | 4-1-0-3 | 4-1-1-2 | 4-1-1-2 | 4-1-0-3 | 4-1-0-3 | 4-2-2-0 | 4-2-2-0 |
| *Rhizopus arrhizus* | 4-2-1-1 | 4-1-0-3 | 4-0-0-0 | 4-0-0-0 | 4-3-0-1 | 4-2-0-2 | 4-2-1-1 | 4-2-1-1 |
| *Rhodotorula mucilaginosa* | 5-0-5-0 | 5-1-4-0 | 5-1-4-0 | 5-1-4-0 | 5-0-5-0 | 5-0-5-0 | 5-0-0-0 | 5-0-0-0 |
| *Saccharomyces cerevisiae* | 5-4-1-0 | 5-3-0-2 | 5-1-4-0 | 5-1-4-0 | 5-4-0-1 | 5-4-0-1 | 5-0-5-0 | 5-0-5-0 |
| *Sarocladium strictum* | 4-1-2-1 | 4-2-0-2 | 4-3-1-0 | 4-3-1-0 | 4-0-0-0 | 4-0-0-0 | 4-0-0-0 | 4-0-0-0 |
| *Scedosporium apiospermum* | 1-0-1-0 | 1-0-1-0 | 1-0-1-0 | 1-0-1-0 | 1-0-0-0 | 1-0-0-0 | 1-0-0-0 | 1-0-0-0 |
| *Scopulariopsis brevicaulis* | 3-1-2-0 | 3-2-1-0 | 3-2-1-0 | 3-2-1-0 | 3-0-3-0 | 3-0-3-0 | 3-0-3-0 | 3-0-3-0 |
| *Sporobolomyces roseus* | 3-0-3-0 | 3-0-3-0 | 3-0-3-0 | 3-0-3-0 | 3-0-3-0 | 3-0-3-0 | 3-0-3-0 | 3-0-3-0 |
| *Sporothrix schenckii* | 3-0-3-0 | 3-1-2-0 | 3-0-3-0 | 3-0-3-0 | 3-0-0-0 | 3-0-0-0 | 3-0-0-0 | 3-0-0-0 |
| *Stachybotrys chartarum* | 8-0-0-0 | 8-2-0-6 | 8-5-2-1 | 8-5-2-1 | 8-0-0-0 | 8-0-0-0 | 8-7-0-1 | 8-7-0-1 |
| *Syncephalastrum racemosum* | 3-0-0-0 | 3-0-0-0 | 3-0-0-0 | 3-0-0-0 | 3-1-0-2 | 3-1-0-2 | 3-0-0-0 | 3-0-0-0 |
| *Thamnidium elegans* | 2-0-0-2 | 2-0-0-2 | 2-0-0-2 | 2-0-0-2 | 2-0-0-2 | 2-0-0-2 | 2-0-1-1 | 2-0-1-1 |
| *Torula herbarum* | 2-0-0-0 | 2-0-0-0 | 2-0-0-0 | 2-0-0-0 | 2-0-0-0 | 2-0-0-0 | 2-0-0-0 | 2-0-0-0 |
| *Trichothecium roseum* | 4-3-0-1 | 4-1-0-3 | 4-2-1-1 | 4-2-1-1 | 4-2-0-2 | 4-2-0-2 | 4-2-2-0 | 4-2-2-0 |
| *Tritirachium oryzae* | 4-0-4-0 | 4-1-3-0 | 4-0-4-0 | 4-0-4-0 | 4-1-3-0 | 4-1-3-0 | 4-0-4-0 | 4-0-4-0 |

Table 19: Read abundance deviation of correctly identified species using Illumina sequences and ITS1 at the genus level. For each combination of database, software and method four numbers are given: the first number indicates the total number of DMCs the species is present in; the second number indicates the number of times the abundance was correct within a 5% margin; the third and fourth numbers indicate the number of times the abundance was overestimated or underestimated by at least 5%, respectively.

|  | **IHEM** | | | | **UNITE** | | | |
| --- | --- | --- | --- | --- | --- | --- | --- | --- |
|  | **BLAST** | | **MOTHUR** | | **BLAST** | | **MOTHUR** | |
| **Species** | **Strict** | **Loose** | **Wang** | **knn** | **Strict** | **Loose** | **Wang** | **knn** |
|  |  |  |  |  |  |  |  |  |
| *Alternaria* | 4-3-0-1 | 4-3-0-1 | 4-4-0-0 | 4-4-0-0 | 4-3-0-1 | 4-3-0-1 | 4-4-0-0 | 4-4-0-0 |
| *Apiospora* | 3-0-0-3 | 3-0-0-3 | 3-0-0-3 | 3-0-0-3 | 3-0-0-0 | 3-0-0-0 | 3-0-0-3 | 3-0-0-3 |
| *Aspergillus* | 22-10-10-2 | 22-10-10-2 | 22-9-11-2 | 22-9-11-2 | 22-6-11-1 | 22-6-11-1 | 22-10-11-1 | 22-10-11-1 |
| *Aureobasidium* | 2-1-1-0 | 2-1-1-0 | 2-1-1-0 | 2-1-1-0 | 2-0-2-0 | 2-0-2-0 | 2-0-0-0 | 2-0-0-0 |
| *Beauveria* | 2-1-1-0 | 2-1-1-0 | 2-1-1-0 | 2-1-1-0 | 2-1-1-0 | 2-1-1-0 | 2-0-0-0 | 2-0-0-0 |
| *Cephalotrichum* | 4-0-0-4 | 4-0-0-4 | 4-0-0-4 | 4-0-0-4 | 4-0-0-4 | 4-0-0-4 | 4-0-0-4 | 4-0-0-4 |
| *Chaetomium* | 2-1-0-1 | 2-1-0-1 | 2-1-0-1 | 2-1-0-1 | 2-1-0-1 | 2-1-0-1 | 2-1-0-1 | 2-1-0-1 |
| *Cladosporium* | 25-8-17-0 | 25-8-17-0 | 25-7-18-0 | 25-7-18-0 | 25-3-15-2 | 25-3-15-2 | 25-3-15-2 | 25-3-15-2 |
| *Cryptococcus* | 5-3-2-0 | 5-3-2-0 | 5-3-2-0 | 5-3-2-0 | 5-2-3-0 | 5-2-3-0 | 5-3-2-0 | 5-3-2-0 |
| *Cunninghamella* | 3-0-0-3 | 3-0-0-3 | 3-0-0-3 | 3-0-0-3 | 3-0-0-3 | 3-0-0-3 | 3-0-0-3 | 3-0-0-3 |
| *Epicoccum* | 2-0-0-2 | 2-0-0-2 | 2-0-0-2 | 2-0-0-2 | 2-0-0-2 | 2-0-0-2 | 2-0-0-0 | 2-0-0-0 |
| *Exophiala* | 4-0-0-4 | 4-0-0-4 | 4-0-0-4 | 4-0-0-4 | 4-0-0-0 | 4-0-0-0 | 4-0-0-4 | 4-0-0-4 |
| *Fusarium* | 4-1-0-3 | 4-1-0-3 | 4-1-0-3 | 4-1-0-3 | 4-1-0-3 | 4-1-0-3 | 4-0-0-0 | 4-0-0-0 |
| *Geotrichum* | 2-2-0-0 | 2-2-0-0 | 2-1-1-0 | 2-1-1-0 | 2-0-0-0 | 2-0-0-0 | 2-0-0-0 | 2-0-0-0 |
| *Gliomastix* | 1-0-0-1 | 1-0-0-1 | 1-0-0-1 | 1-0-0-1 | 1-0-0-1 | 1-0-0-1 | 1-0-0-1 | 1-0-0-1 |
| *Lichtheimia* | 4-0-0-4 | 4-0-0-4 | 4-0-0-4 | 4-0-0-4 | 4-0-0-4 | 4-0-0-4 | 4-0-0-4 | 4-0-0-4 |
| *Memnoniella* | 3-2-0-1 | 3-2-0-1 | 3-2-0-1 | 3-2-0-1 | 3-2-0-1 | 3-2-0-1 | 3-2-0-1 | 3-2-0-1 |
| *Paecilomyces* | 3-2-0-1 | 3-2-0-1 | 3-2-0-1 | 3-2-0-1 | 3-1-1-1 | 3-1-1-1 | 3-3-0-0 | 3-3-0-0 |
| *Penicillium* | 29-14-5-10 | 29-14-5-10 | 29-15-5-9 | 29-15-5-9 | 29-10-5-10 | 29-10-5-10 | 29-12-8-9 | 29-12-8-9 |
| *Phoma* | 2-1-1-0 | 2-1-1-0 | 2-0-0-0 | 2-0-0-0 | 2-1-1-0 | 2-1-1-0 | 2-0-0-0 | 2-0-0-0 |
| *Purpureocillium* | 4-1-0-3 | 4-1-0-3 | 4-2-0-2 | 4-2-0-2 | 4-1-0-3 | 4-1-0-3 | 4-2-0-2 | 4-2-0-2 |
| *Rhizopus* | 4-1-0-3 | 4-1-0-3 | 4-1-0-3 | 4-1-0-3 | 4-2-0-2 | 4-2-0-2 | 4-1-0-3 | 4-1-0-3 |
| *Rhodotorula* | 5-1-4-0 | 5-1-4-0 | 5-1-4-0 | 5-1-4-0 | 5-0-5-0 | 5-0-5-0 | 5-0-5-0 | 5-0-5-0 |
| *Saccharomyces* | 5-3-0-2 | 5-3-0-2 | 5-2-1-2 | 5-2-1-2 | 5-4-0-1 | 5-4-0-1 | 5-2-1-2 | 5-2-1-2 |
| *Sarocladium* | 4-2-0-2 | 4-2-0-2 | 4-2-0-2 | 4-2-0-2 | 4-2-0-2 | 4-2-0-2 | 4-3-0-1 | 4-3-0-1 |
| *Scedosporium* | 1-0-1-0 | 1-0-1-0 | 1-0-1-0 | 1-0-1-0 | 1-0-0-0 | 1-0-0-0 | 1-0-0-0 | 1-0-0-0 |
| *Scopulariopsis* | 3-2-1-0 | 3-2-1-0 | 3-2-1-0 | 3-2-1-0 | 3-0-3-0 | 3-0-3-0 | 3-2-1-0 | 3-2-1-0 |
| *Sporobolomyces* | 3-0-3-0 | 3-0-3-0 | 3-0-3-0 | 3-0-3-0 | 3-0-3-0 | 3-0-3-0 | 3-0-3-0 | 3-0-3-0 |
| *Sporothrix* | 3-1-2-0 | 3-1-2-0 | 3-1-2-0 | 3-1-2-0 | 3-1-2-0 | 3-1-2-0 | 3-1-2-0 | 3-1-2-0 |
| *Stachybotrys* | 8-2-0-6 | 8-2-0-6 | 8-2-0-6 | 8-2-0-6 | 8-0-0-0 | 8-0-0-0 | 8-3-0-5 | 8-3-0-5 |
| *Syncephalastrum* | 3-0-0-0 | 3-0-0-0 | 3-0-0-3 | 3-0-0-3 | 3-1-0-2 | 3-1-0-2 | 3-0-0-3 | 3-0-0-3 |
| *Thamnidium* | 2-0-0-2 | 2-0-0-2 | 2-0-0-2 | 2-0-0-2 | 2-0-0-2 | 2-0-0-2 | 2-0-0-2 | 2-0-0-2 |
| *Torula* | 2-0-0-0 | 2-0-0-0 | 2-0-0-0 | 2-0-0-0 | 2-0-0-1 | 2-0-0-1 | 2-1-0-1 | 2-1-0-1 |
| *Trichothecium* | 4-1-0-3 | 4-1-0-3 | 4-2-0-2 | 4-2-0-2 | 4-2-0-2 | 4-2-0-2 | 4-1-0-3 | 4-1-0-3 |
| *Tritirachium* | 4-1-3-0 | 4-1-3-0 | 4-1-3-0 | 4-1-3-0 | 4-1-3-0 | 4-1-3-0 | 4-1-3-0 | 4-1-3-0 |

Table 20: Read abundance deviation of correctly identified species using Illumina sequences and ITS2 at the species level. For each combination of database, software and method four numbers are given: the first number indicates the total number of DMCs the species is present in; the second number indicates the number of times the abundance was correct within a 5% margin; the third and fourth numbers indicate the number of times the abundance was overestimated or underestimated by at least 5%, respectively.

|  | **IHEM** | | | | **UNITE** | | | |
| --- | --- | --- | --- | --- | --- | --- | --- | --- |
|  | **BLAST** | | **MOTHUR** | | **BLAST** | | **MOTHUR** | |
| **Species** | **Strict** | **Loose** | **Wang** | **knn** | **Strict** | **Loose** | **Wang** | **knn** |
|  |  |  |  |  |  |  |  |  |
| *Alternaria alternata* | 4-1-3-0 | 4-4-0-0 | 4-2-2-0 | 4-2-2-0 | 4-0-0-0 | 4-3-1-0 | 4-1-3-0 | 4-1-3-0 |
| *Apiospora montagnei* | 3-0-0-3 | 3-0-0-3 | 3-0-0-3 | 3-0-0-3 | 3-0-0-0 | 3-0-0-0 | 3-0-0-0 | 3-0-0-0 |
| *Aspergillus chevalieri* | 2-0-0-0 | 2-2-0-0 | 2-0-0-0 | 2-0-0-0 | 2-0-0-0 | 2-0-0-0 | 2-0-0-0 | 2-0-0-0 |
| *Aspergillus floridensis* | 1-0-0-0 | 1-0-1-0 | 1-0-0-0 | 1-0-0-0 | 1-0-0-0 | 1-0-0-0 | 1-0-0-0 | 1-0-0-0 |
| *Aspergillus fumigatus* | 6-0-0-0 | 6-2-4-0 | 6-1-5-0 | 6-1-5-0 | 6-1-5-0 | 6-2-4-0 | 6-0-0-0 | 6-0-0-0 |
| *Aspergillus ochraceus* | 2-0-0-0 | 2-1-1-0 | 2-0-0-0 | 2-0-0-0 | 2-0-0-0 | 2-0-2-0 | 2-0-2-0 | 2-0-2-0 |
| *Aspergillus restrictus* | 2-1-1-0 | 2-2-0-0 | 2-1-1-0 | 2-1-1-0 | 2-1-1-0 | 2-1-1-0 | 2-1-1-0 | 2-1-1-0 |
| *Aspergillus subalbidus* | 1-0-0-0 | 1-1-0-0 | 1-1-0-0 | 1-1-0-0 | 1-0-0-0 | 1-0-1-0 | 1-0-0-0 | 1-0-0-0 |
| *Aspergillus ustus* | 4-0-0-0 | 4-2-0-2 | 4-0-0-0 | 4-0-0-0 | 4-0-0-0 | 4-4-0-0 | 4-0-0-0 | 4-0-0-0 |
| *Aspergillus versicolor* | 5-0-0-0 | 5-2-1-2 | 5-0-0-0 | 5-0-0-0 | 5-0-0-0 | 5-0-0-0 | 5-0-0-0 | 5-0-0-0 |
| *Aureobasidium pullulans* | 2-1-1-0 | 2-1-1-0 | 2-1-1-0 | 2-1-1-0 | 2-1-1-0 | 2-1-1-0 | 2-0-2-0 | 2-0-2-0 |
| *Beauveria bassiana* | 2-0-2-0 | 2-0-2-0 | 2-0-2-0 | 2-0-2-0 | 2-0-0-0 | 2-0-2-0 | 2-0-2-0 | 2-0-2-0 |
| *Cephalotrichum stemonitis* | 4-0-0-4 | 4-0-0-4 | 4-0-0-0 | 4-0-0-0 | 4-1-0-3 | 4-0-0-4 | 4-0-0-4 | 4-0-0-4 |
| *Chaetomium globosum* | 2-0-2-0 | 2-1-0-1 | 2-2-0-0 | 2-2-0-0 | 2-0-0-0 | 2-1-1-0 | 2-0-0-0 | 2-0-0-0 |
| *Cladosporium cladosporioides* | 10-0-0-0 | 10-0-10-0 | 10-0-10-0 | 10-0-10-0 | 10-0-0-0 | 10-0-10-0 | 10-0-0-0 | 10-0-0-0 |
| *Cladosporium herbarum* | 7-0-0-0 | 7-1-6-0 | 7-0-0-0 | 7-0-0-0 | 7-0-0-0 | 7-0-0-0 | 7-0-0-0 | 7-0-0-0 |
| *Cladosporium sphaerospermum* | 8-0-8-0 | 8-3-5-0 | 8-3-5-0 | 8-3-5-0 | 8-1-7-0 | 8-1-7-0 | 8-0-8-0 | 8-0-8-0 |
| *Cryptococcus neoformans* var. grubii | 5-0-0-0 | 5-0-5-0 | 5-0-5-0 | 5-0-5-0 | 5-0-5-0 | 5-0-5-0 | 5-0-5-0 | 5-0-5-0 |
| *Cunninghamella echinulata* | 3-0-0-0 | 3-0-0-0 | 3-0-0-3 | 3-0-0-3 | 3-0-0-0 | 3-0-0-0 | 3-0-0-3 | 3-0-0-3 |
| *Epicoccum nigrum* | 2-0-2-0 | 2-1-0-1 | 2-1-0-1 | 2-1-0-1 | 2-0-0-0 | 2-1-0-1 | 2-0-0-0 | 2-0-0-0 |
| *Exophiala jeanselmei* | 4-0-0-4 | 4-0-0-4 | 4-0-0-4 | 4-0-0-4 | 4-0-0-0 | 4-0-0-0 | 4-0-0-0 | 4-0-0-0 |
| *Fusarium culmorum* | 4-0-0-0 | 4-0-1-3 | 4-1-2-1 | 4-1-2-1 | 4-0-0-0 | 4-0-0-0 | 4-0-0-0 | 4-0-0-0 |
| *Geotrichum candidum* | 2-0-2-0 | 2-1-1-0 | 2-0-2-0 | 2-0-2-0 | 2-0-2-0 | 2-1-1-0 | 2-1-1-0 | 2-1-1-0 |
| *Gliomastix murorum* | 1-0-0-1 | 1-0-0-1 | 1-0-0-1 | 1-0-0-1 | 1-0-0-0 | 1-0-0-0 | 1-0-0-0 | 1-0-0-0 |
| *Lichtheimia corymbifera* | 4-2-1-1 | 4-2-0-2 | 4-3-0-1 | 4-3-0-1 | 4-1-1-2 | 4-2-0-2 | 4-1-1-2 | 4-1-1-2 |
| *Memnoniella echinata* | 3-2-1-0 | 3-0-0-3 | 3-2-0-1 | 3-2-0-1 | 3-0-0-0 | 3-0-0-0 | 3-0-0-0 | 3-0-0-0 |
| *Paecilomyces variotii* | 3-1-2-0 | 3-1-0-2 | 3-0-1-2 | 3-0-1-2 | 3-0-0-0 | 3-1-0-2 | 3-2-1-0 | 3-2-1-0 |
| *Penicillium aurantiogriseum* | 5-0-0-0 | 5-3-0-2 | 5-0-0-0 | 5-0-0-0 | 5-1-2-2 | 5-3-0-2 | 5-2-3-0 | 5-2-3-0 |
| *Penicillium brevicompactum* | 5-3-1-1 | 5-1-0-4 | 5-1-0-4 | 5-1-0-4 | 5-0-0-0 | 5-1-0-4 | 5-2-0-3 | 5-2-0-3 |
| *Penicillium chrysogenum* | 8-0-0-0 | 8-0-0-8 | 8-0-0-0 | 8-0-0-0 | 8-0-0-0 | 8-0-0-8 | 8-0-0-0 | 8-0-0-0 |
| *Penicillium citreonigrum* | 2-1-1-0 | 2-1-0-1 | 2-1-0-1 | 2-1-0-1 | 2-1-1-0 | 2-1-0-1 | 2-2-0-0 | 2-2-0-0 |
| *Penicillium corylophilum* | 2-0-2-0 | 2-1-1-0 | 2-1-1-0 | 2-1-1-0 | 2-0-2-0 | 2-1-1-0 | 2-1-1-0 | 2-1-1-0 |
| *Penicillium digitatum* | 4-1-3-0 | 4-2-2-0 | 4-1-3-0 | 4-1-3-0 | 4-0-0-0 | 4-0-0-0 | 4-0-0-0 | 4-0-0-0 |
| *Penicillium glabrum* | 2-0-0-0 | 2-2-0-0 | 2-2-0-0 | 2-2-0-0 | 2-0-0-0 | 2-0-0-0 | 2-0-0-0 | 2-0-0-0 |
| *Penicillium halotolerans* | 4-0-0-0 | 4-2-2-0 | 4-0-0-0 | 4-0-0-0 | 4-0-0-0 | 4-2-2-0 | 4-0-0-0 | 4-0-0-0 |
| *Phoma herbarum* | 2-0-2-0 | 2-1-1-0 | 2-0-0-0 | 2-0-0-0 | 2-0-2-0 | 2-0-2-0 | 2-0-0-0 | 2-0-0-0 |
| *Purpureocillium lilacinum* | 4-0-0-0 | 4-3-0-1 | 4-4-0-0 | 4-4-0-0 | 4-0-0-0 | 4-0-0-0 | 4-0-0-0 | 4-0-0-0 |
| *Rhizopus arrhizus* | 4-2-2-0 | 4-2-0-2 | 4-0-0-0 | 4-0-0-0 | 4-0-3-1 | 4-2-0-2 | 4-3-1-0 | 4-3-1-0 |
| *Rhodotorula mucilaginosa* | 5-0-5-0 | 5-0-5-0 | 5-0-5-0 | 5-0-5-0 | 5-0-5-0 | 5-0-5-0 | 5-0-5-0 | 5-0-5-0 |
| *Saccharomyces cerevisiae* | 5-0-5-0 | 5-1-4-0 | 5-0-5-0 | 5-0-5-0 | 5-0-0-2 | 5-0-0-2 | 5-0-0-0 | 5-0-0-0 |
| *Sarocladium strictum* | 4-1-3-0 | 4-2-0-2 | 4-3-1-0 | 4-3-1-0 | 4-0-0-0 | 4-0-0-0 | 4-0-0-0 | 4-0-0-0 |
| *Scedosporium apiospermum* | 1-0-1-0 | 1-0-1-0 | 1-0-1-0 | 1-0-1-0 | 1-0-0-0 | 1-0-0-0 | 1-0-0-0 | 1-0-0-0 |
| *Scopulariopsis brevicaulis* | 3-0-3-0 | 3-2-1-0 | 3-2-1-0 | 3-2-1-0 | 3-0-3-0 | 3-0-3-0 | 3-0-0-0 | 3-0-0-0 |
| *Sporobolomyces roseus* | 3-0-3-0 | 3-0-3-0 | 3-0-3-0 | 3-0-3-0 | 3-0-3-0 | 3-0-3-0 | 3-0-3-0 | 3-0-3-0 |
| *Sporothrix schenckii* | 3-3-0-0 | 3-2-0-1 | 3-2-0-1 | 3-2-0-1 | 3-2-1-0 | 3-2-0-1 | 3-2-0-1 | 3-2-0-1 |
| *Stachybotrys chartarum* | 8-3-3-2 | 8-1-0-7 | 8-1-1-6 | 8-1-1-6 | 8-3-2-3 | 8-1-0-7 | 8-3-1-4 | 8-3-1-4 |
| *Syncephalastrum racemosum* | 3-0-0-0 | 3-0-0-0 | 3-0-0-0 | 3-0-0-0 | 3-0-0-0 | 3-0-0-0 | 3-0-0-0 | 3-0-0-0 |
| *Thamnidium elegans* | 2-0-0-2 | 2-0-0-2 | 2-0-0-2 | 2-0-0-2 | 2-0-0-2 | 2-0-0-2 | 2-0-0-2 | 2-0-0-2 |
| *Torula herbarum* | 2-0-0-0 | 2-0-0-0 | 2-0-0-0 | 2-0-0-0 | 2-0-0-0 | 2-0-0-0 | 2-0-0-0 | 2-0-0-0 |
| *Trichothecium roseum* | 4-2-1-1 | 4-1-0-3 | 4-0-1-3 | 4-0-1-3 | 4-0-1-3 | 4-1-0-3 | 4-2-1-1 | 4-2-1-1 |
| *Tritirachium oryzae* | 4-0-4-0 | 4-0-4-0 | 4-0-4-0 | 4-0-4-0 | 4-0-4-0 | 4-0-4-0 | 4-0-4-0 | 4-0-4-0 |

Table 21: Read abundance deviation of correctly identified species using Illumina sequences and ITS2 at the genus level. For each combination of database, software and method four numbers are given: the first number indicates the total number of DMCs the species is present in; the second number indicates the number of times the abundance was correct within a 5% margin; the third and fourth numbers indicate the number of times the abundance was overestimated or underestimated by at least 5%, respectively.

|  | **IHEM** | | | | **UNITE** | | | |
| --- | --- | --- | --- | --- | --- | --- | --- | --- |
|  | **BLAST** | | **MOTHUR** | | **BLAST** | | **MOTHUR** | |
| **Species** | **Strict** | **Loose** | **Wang** | **knn** | **Strict** | **Loose** | **Wang** | **knn** |
|  |  |  |  |  |  |  |  |  |
| *Alternaria* | 4-3-1-0 | 4-4-0-0 | 4-4-0-0 | 4-4-0-0 | 4-2-2-0 | 4-3-1-0 | 4-3-1-0 | 4-3-1-0 |
| *Apiospora* | 3-0-0-3 | 3-0-0-3 | 3-0-0-3 | 3-0-0-3 | 3-0-0-0 | 3-0-0-0 | 3-1-0-2 | 3-1-0-2 |
| *Aspergillus* | 22-10-3-4 | 22-11-7-4 | 22-9-8-5 | 22-9-8-5 | 22-10-8-4 | 22-10-8-4 | 22-10-8-4 | 22-10-8-4 |
| *Aureobasidium* | 2-1-1-0 | 2-1-1-0 | 2-1-1-0 | 2-1-1-0 | 2-0-2-0 | 2-1-1-0 | 2-1-1-0 | 2-1-1-0 |
| *Beauveria* | 2-0-2-0 | 2-0-2-0 | 2-0-2-0 | 2-0-2-0 | 2-0-0-0 | 2-0-2-0 | 2-0-2-0 | 2-0-2-0 |
| *Cephalotrichum* | 4-0-0-4 | 4-0-0-4 | 4-0-0-4 | 4-0-0-4 | 4-0-0-4 | 4-0-0-4 | 4-0-0-4 | 4-0-0-4 |
| *Chaetomium* | 2-0-1-1 | 2-1-0-1 | 2-1-0-1 | 2-1-0-1 | 2-0-1-1 | 2-0-1-1 | 2-0-1-1 | 2-0-1-1 |
| *Cladosporium* | 25-3-22-0 | 25-5-20-0 | 25-5-20-0 | 25-5-20-0 | 25-2-16-0 | 25-2-16-0 | 25-3-15-0 | 25-3-15-0 |
| *Cryptococcus* | 5-0-5-0 | 5-0-5-0 | 5-0-5-0 | 5-0-5-0 | 5-0-5-0 | 5-0-5-0 | 5-0-5-0 | 5-0-5-0 |
| *Cunninghamella* | 3-0-0-0 | 3-0-0-0 | 3-0-0-3 | 3-0-0-3 | 3-0-0-0 | 3-0-0-0 | 3-0-0-3 | 3-0-0-3 |
| *Epicoccum* | 2-0-0-2 | 2-0-0-2 | 2-0-0-2 | 2-0-0-2 | 2-0-0-0 | 2-0-0-2 | 2-0-0-0 | 2-0-0-0 |
| *Exophiala* | 4-0-0-4 | 4-0-0-4 | 4-0-0-4 | 4-0-0-4 | 4-0-0-0 | 4-0-0-0 | 4-0-1-3 | 4-0-1-3 |
| *Fusarium* | 4-1-0-3 | 4-1-0-3 | 4-1-0-3 | 4-1-0-3 | 4-0-0-0 | 4-1-1-2 | 4-2-1-1 | 4-2-1-1 |
| *Geotrichum* | 2-1-1-0 | 2-1-1-0 | 2-1-1-0 | 2-1-1-0 | 2-0-0-0 | 2-0-2-0 | 2-1-1-0 | 2-1-1-0 |
| *Gliomastix* | 1-0-0-1 | 1-0-0-1 | 1-0-0-1 | 1-0-0-1 | 1-0-0-0 | 1-0-0-0 | 1-0-0-0 | 1-0-0-0 |
| *Lichtheimia* | 4-1-0-3 | 4-1-0-3 | 4-1-0-3 | 4-1-0-3 | 4-2-0-2 | 4-1-0-3 | 4-1-0-3 | 4-1-0-3 |
| *Memnoniella* | 3-1-0-2 | 3-0-0-3 | 3-0-0-3 | 3-0-0-3 | 3-0-0-0 | 3-0-0-0 | 3-0-0-0 | 3-0-0-0 |
| *Paecilomyces* | 3-1-0-2 | 3-1-0-2 | 3-1-0-2 | 3-1-0-2 | 3-0-0-0 | 3-1-0-2 | 3-0-1-2 | 3-0-1-2 |
| *Penicillium* | 29-12-6-11 | 29-11-6-12 | 29-11-6-12 | 29-11-6-12 | 29-7-7-11 | 29-9-4-12 | 29-8-10-11 | 29-8-10-11 |
| *Phoma* | 2-1-1-0 | 2-1-1-0 | 2-0-0-0 | 2-0-0-0 | 2-0-2-0 | 2-0-2-0 | 2-0-0-0 | 2-0-0-0 |
| *Purpureocillium* | 4-2-1-1 | 4-3-0-1 | 4-3-0-1 | 4-3-0-1 | 4-0-0-0 | 4-0-0-0 | 4-4-0-0 | 4-4-0-0 |
| *Rhizopus* | 4-2-0-2 | 4-2-0-2 | 4-2-0-2 | 4-2-0-2 | 4-2-0-2 | 4-2-0-2 | 4-2-0-2 | 4-2-0-2 |
| *Rhodotorula* | 5-0-5-0 | 5-0-5-0 | 5-0-5-0 | 5-0-5-0 | 5-0-5-0 | 5-0-5-0 | 5-0-5-0 | 5-0-5-0 |
| *Saccharomyces* | 5-1-4-0 | 5-1-4-0 | 5-1-4-0 | 5-1-4-0 | 5-1-4-0 | 5-1-4-0 | 5-1-4-0 | 5-1-4-0 |
| *Sarocladium* | 4-2-0-2 | 4-2-0-2 | 4-2-0-2 | 4-2-0-2 | 4-2-1-1 | 4-2-0-2 | 4-3-0-1 | 4-3-0-1 |
| *Scedosporium* | 1-0-1-0 | 1-0-1-0 | 1-0-1-0 | 1-0-1-0 | 1-0-0-0 | 1-0-0-0 | 1-0-0-0 | 1-0-0-0 |
| *Scopulariopsis* | 3-1-2-0 | 3-2-1-0 | 3-2-1-0 | 3-2-1-0 | 3-0-3-0 | 3-0-3-0 | 3-0-0-0 | 3-0-0-0 |
| *Sporobolomyces* | 3-0-3-0 | 3-0-3-0 | 3-0-3-0 | 3-0-3-0 | 3-0-3-0 | 3-0-3-0 | 3-0-3-0 | 3-0-3-0 |
| *Sporothrix* | 3-2-0-1 | 3-2-0-1 | 3-2-0-1 | 3-2-0-1 | 3-1-1-1 | 3-2-0-1 | 3-2-0-1 | 3-2-0-1 |
| *Stachybotrys* | 8-1-0-7 | 8-0-0-8 | 8-0-0-8 | 8-0-0-8 | 8-2-0-6 | 8-0-0-8 | 8-1-0-7 | 8-1-0-7 |
| *Syncephalastrum* | 3-0-0-1 | 3-0-0-1 | 3-0-0-1 | 3-0-0-1 | 3-0-0-0 | 3-0-0-0 | 3-0-0-1 | 3-0-0-1 |
| *Thamnidium* | 2-0-0-2 | 2-0-0-2 | 2-0-0-2 | 2-0-0-2 | 2-0-0-2 | 2-0-0-2 | 2-0-0-2 | 2-0-0-2 |
| *Torula* | 2-0-0-0 | 2-0-0-0 | 2-0-0-0 | 2-0-0-0 | 2-1-0-1 | 2-1-0-1 | 2-1-0-1 | 2-1-0-1 |
| *Trichothecium* | 4-2-0-2 | 4-1-0-3 | 4-1-0-3 | 4-1-0-3 | 4-1-0-3 | 4-1-0-3 | 4-1-0-3 | 4-1-0-3 |
| *Tritirachium* | 4-0-4-0 | 4-0-4-0 | 4-0-4-0 | 4-0-4-0 | 4-0-4-0 | 4-0-4-0 | 4-0-4-0 | 4-0-4-0 |

Table 22: Overview of the number of operational taxonomic units that were counted as unclassified for each combination of database, software, setting, taxonomic level, and ITS region.

|  |  |  |  | **ITS1** | **ITS2** |
| --- | --- | --- | --- | --- | --- |
| **IHEM** | **BLAST** | **strict** | **species** | 117 | 85 |
|  |  |  | **genus** | 0 | 11 |
|  |  | **loose** | **species** | 0 | 0 |
|  |  |  | **genus** | 0 | 0 |
|  | **mothur** | **Wang** | **species** | 121 | 63 |
|  |  |  | **genus** | 37 | 15 |
|  |  | **knn** | **species** | 0 | 0 |
|  |  |  | **genus** | 0 | 0 |
| **UNITE** | **BLAST** | **strict** | **species** | 79 | 95 |
|  |  |  | **genus** | 32 | 47 |
|  |  | **loose** | **species** | 55 | 33 |
|  |  |  | **genus** | 32 | 25 |
|  | **mothur** | **Wang** | **species** | 176 | 124 |
|  |  |  | **genus** | 68 | 33 |
|  |  | **knn** | **species** | 115 | 108 |
|  |  |  | **genus** | 74 | 80 |

# Figures


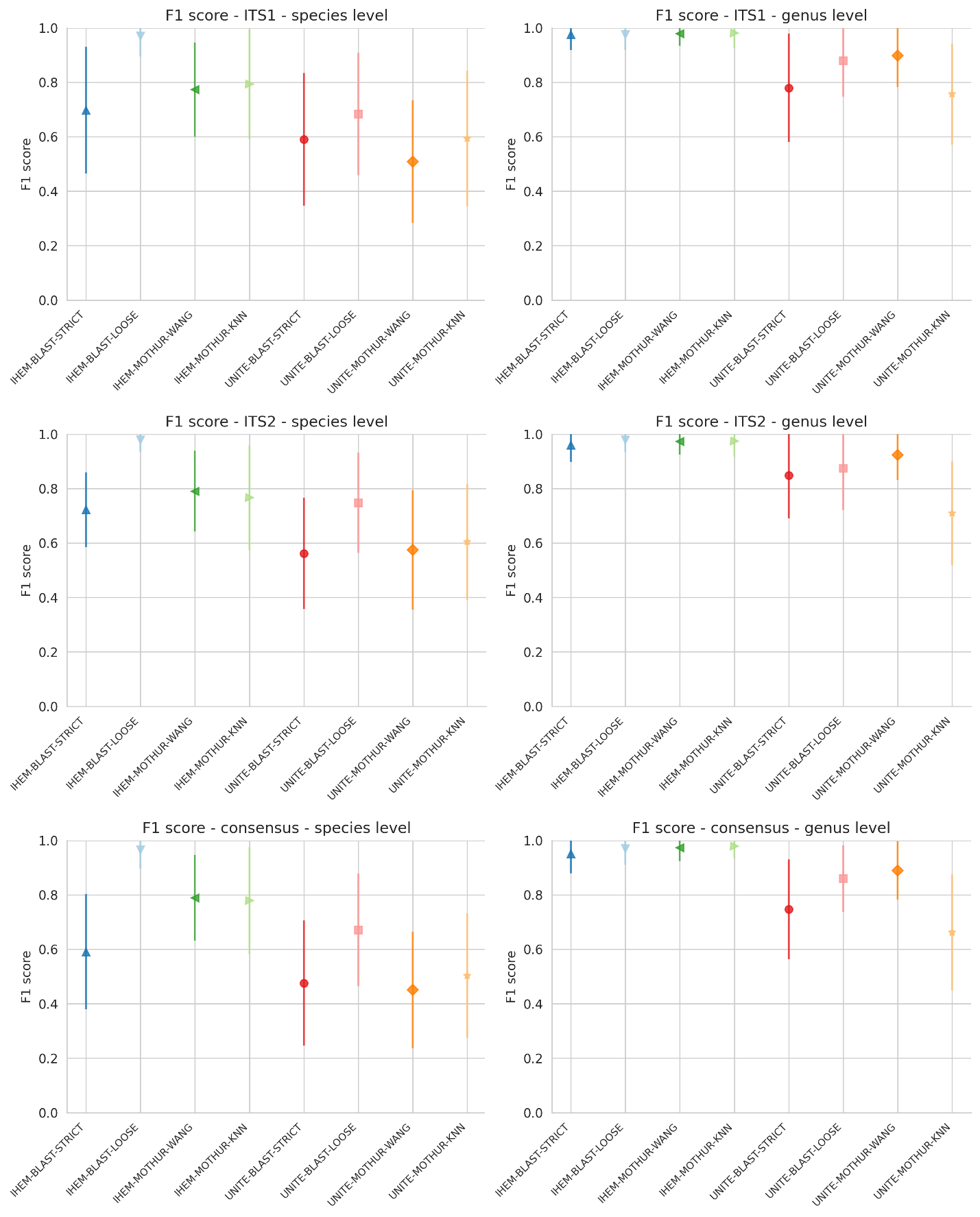
Figure 1: F1 scores per ITS region and taxonomic level used for classification of Sanger sequences, calculated for all considered combinations of database, software and setting. Average values (symbols) and standard deviation (error bars) for the F1 scores are shown, calculated over all DMCs.


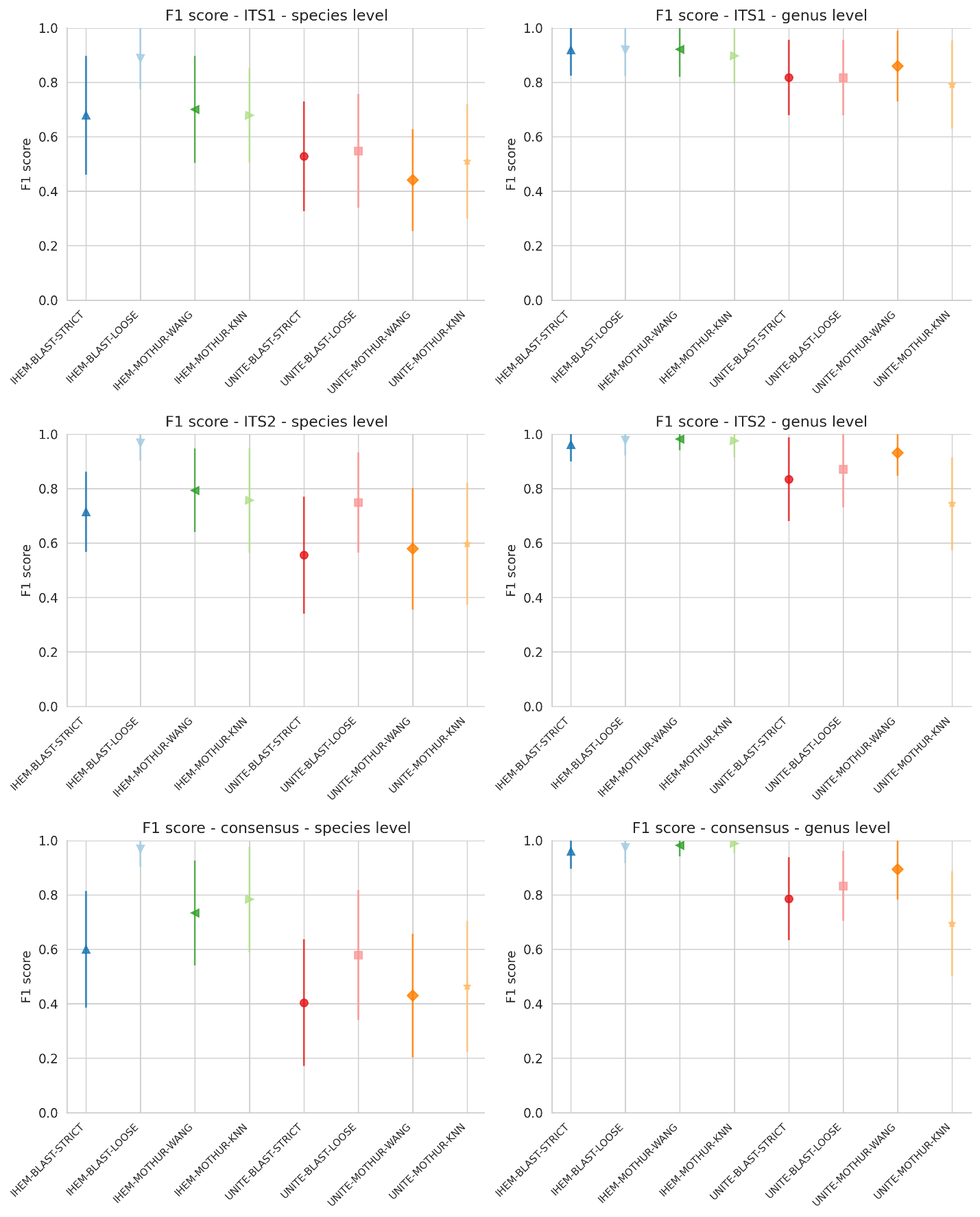


Figure 2: F1 scores per ITS region and taxonomic level used for classification of MiSeq sequences, calculated for all considered combinations of database, software and setting. Average values (symbols) and standard deviation (error bars) for the F1 scores are shown, calculated over all DMCs.


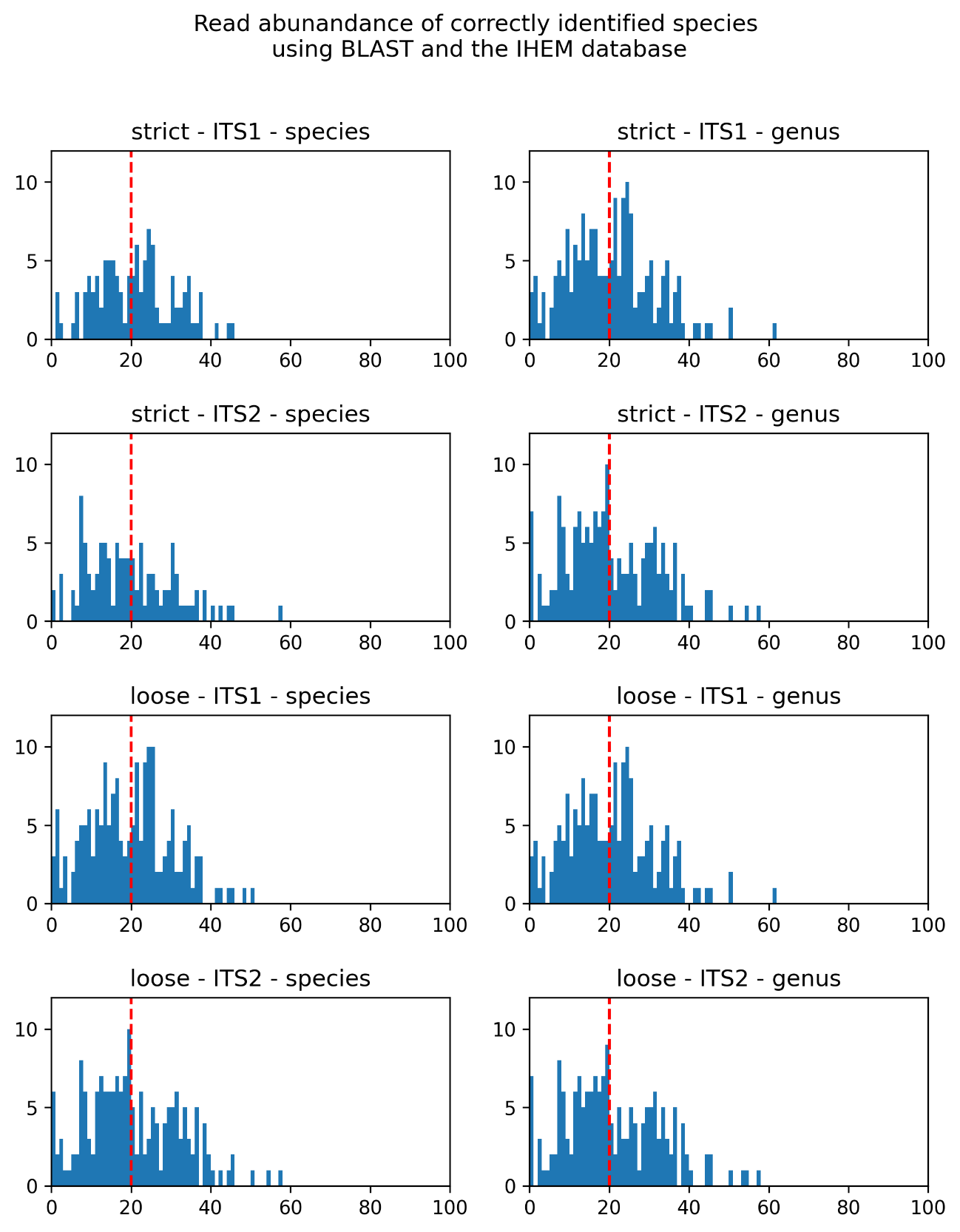


Figure 3: Read abundance of correctly identified species over all samples using BLAST and the IHEM database. Each subgraph shows the histogram for the indicated combination of software setting, ITS region, and taxonomic level. The abscissa denotes the abundance (in %) while the ordinate denotes the number of correct classifications with that abundance. The red dotted line shows the theoretical abundance at which each species should be present.


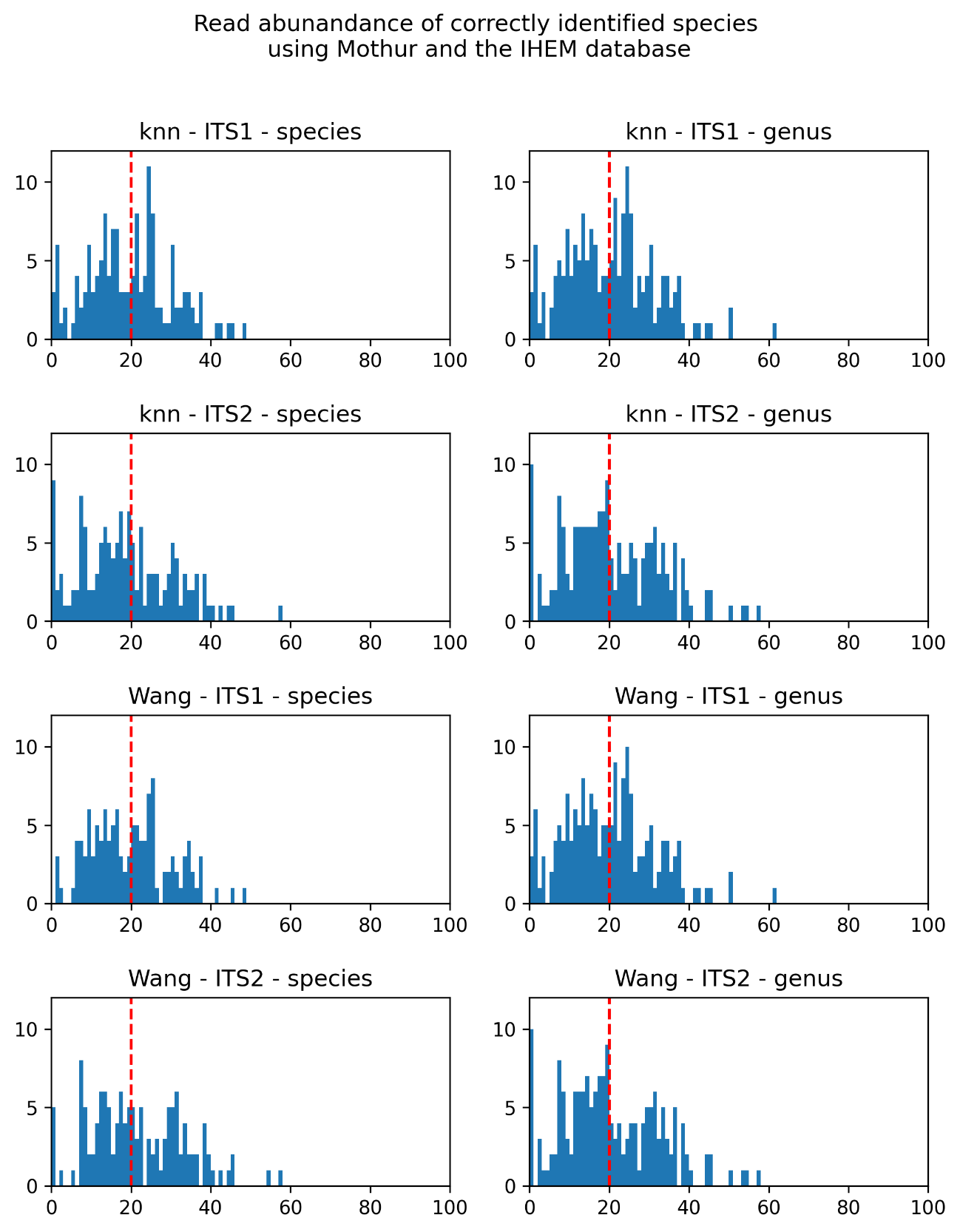


Figure 4: Read abundance of correctly identified species over all samples using Mothur and the IHEM database. Each subgraph shows the histogram for the given combination of software setting, ITS region, and taxonomic level. The abscissa denotes the abundance (in %) while the ordinate denotes the number of correct classifications with that abundance. The red dotted line shows the theoretical abundance at which each species should be present.


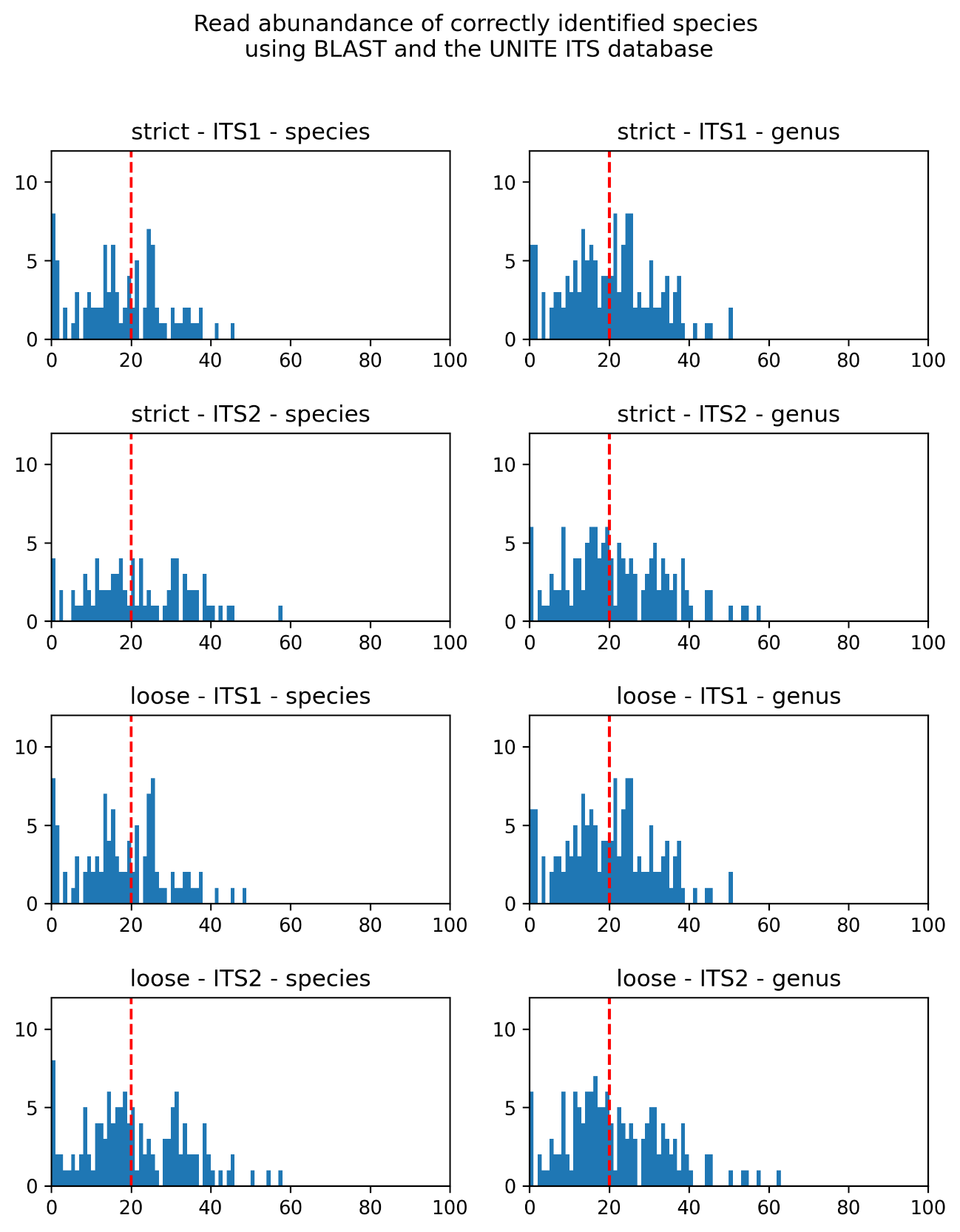


Figure 5: Read abundance of correctly identified species over all samples using BLAST and the UNITE ITS database. Each subgraph shows the histogram for the given combination of software setting, ITS region, and taxonomic level. The abscissa denotes the abundance (in %) while the ordinate denotes the number of correct classifications with that abundance. The red dotted line shows the theoretical abundance at which each species should be present.


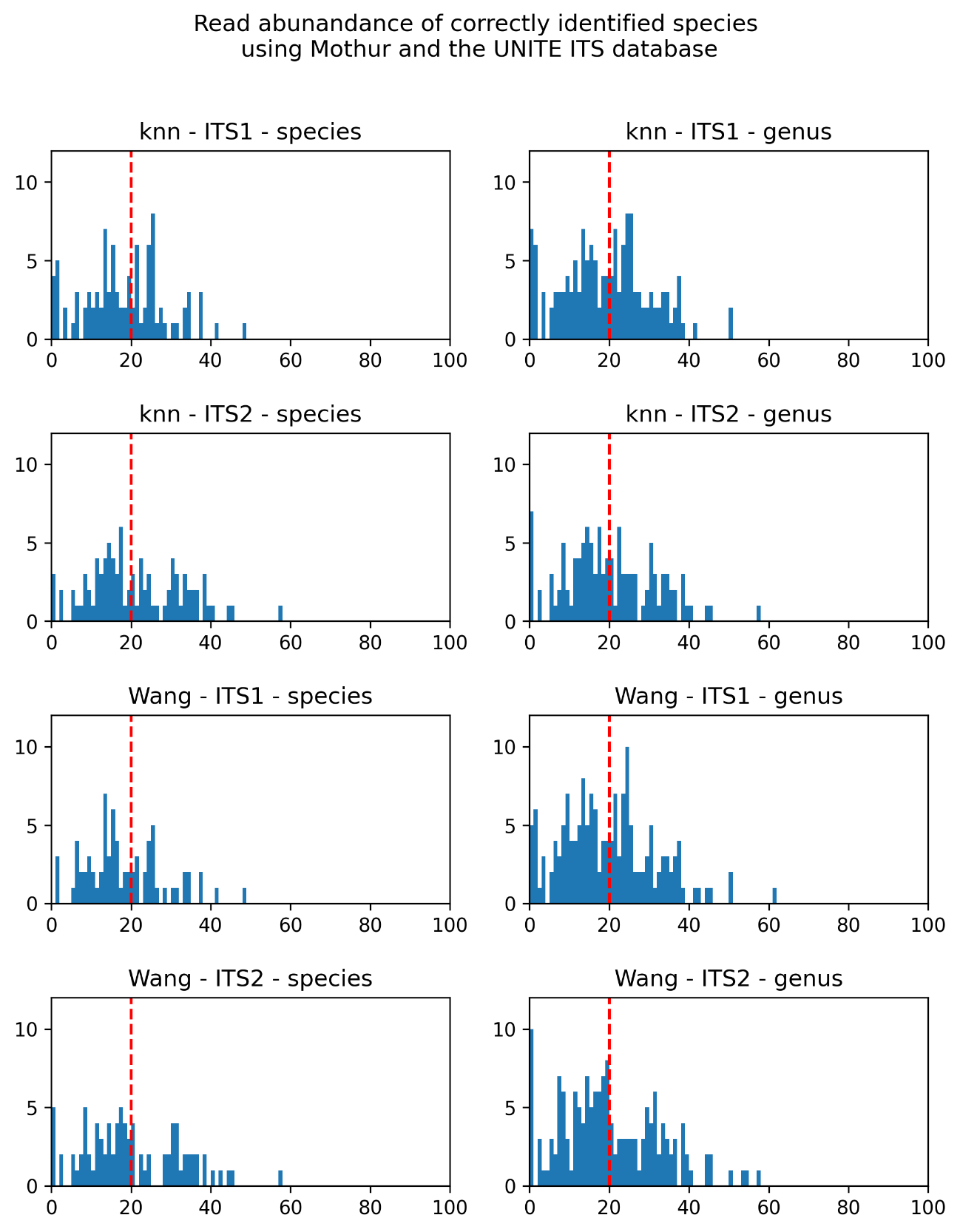


Figure 6: Read abundance of correctly identified species over all samples using Mothur and the UNITE ITS database. Each subgraph shows the histogram for the given combination of software setting, ITS region, and taxonomic level. The abscissa denotes the abundance (in %) while the ordinate denotes the number of correct classifications with that abundance. The red dotted line shows the theoretical abundance at which each species should be present.
